# A year of genomic surveillance reveals how the SARS-CoV-2 pandemic unfolded in Africa

**DOI:** 10.1126/science.abj4336

**Published:** 2021-09-09

**Authors:** Eduan Wilkinson, Marta Giovanetti, Houriiyah Tegally, James E. San, Richard Lessells, Diego Cuadros, Darren P. Martin, David A. Rasmussen, Abdel-Rahman N. Zekri, Abdoul K. Sangare, Abdoul-Salam Ouedraogo, Abdul K. Sesay, Abechi Priscilla, Adedotun-Sulaiman Kemi, Adewunmi M. Olubusuyi, Adeyemi O. O. Oluwapelumi, Adnène Hammami, Adrienne A. Amuri, Ahmad Sayed, Ahmed E. O. Ouma, Aida Elargoubi, Nnennaya A. Ajayi, Ajogbasile F. Victoria, Akano Kazeem, Akpede George, Alexander J. Trotter, Ali A. Yahaya, Alpha K. Keita, Amadou Diallo, Amadou Kone, Amal Souissi, Amel Chtourou, Ana V. Gutierrez, Andrew J. Page, Anika Vinze, Arash Iranzadeh, Arnold Lambisia, Arshad Ismail, Audu Rosemary, Augustina Sylverken, Ayoade Femi, Azeddine Ibrahimi, Baba Marycelin, Bamidele S. Oderinde, Bankole Bolajoko, Beatrice Dhaala, Belinda L. Herring, Berthe-Marie Njanpop-Lafourcade, Bronwyn Kleinhans, Bronwyn McInnis, Bryan Tegomoh, Cara Brook, Catherine B. Pratt, Cathrine Scheepers, Chantal G. Akoua-Koffi, Charles N. Agoti, Christophe Peyrefitte, Claudia Daubenberger, Collins M. Morang’a, D. James Nokes, Daniel G. Amoako, Daniel L. Bugembe, Danny Park, David Baker, Deelan Doolabh, Deogratius Ssemwanga, Derek Tshiabuila, Diarra Bassirou, Dominic S. Y. Amuzu, Dominique Goedhals, Donwilliams O. Omuoyo, Dorcas Maruapula, Ebenezer Foster-Nyarko, Eddy K. Lusamaki, Edgar Simulundu, Edidah M. Ong’era, Edith N. Ngabana, Edwin Shumba, Elmostafa El Fahime, Emmanuel Lokilo, Enatha Mukantwari, Eromon Philomena, Essia Belarbi, Etienne Simon-Loriere, Etilé A. Anoh, Fabian Leendertz, Faida Ajili, Fakayode O. Enoch, Fares Wasfi, Fatma Abdelmoula, Fausta S. Mosha, Faustinos T. Takawira, Fawzi Derrar, Feriel Bouzid, Folarin Onikepe, Fowotade Adeola, Francisca M. Muyembe, Frank Tanser, Fred A. Dratibi, Gabriel K. Mbunsu, Gaetan Thilliez, Gemma L. Kay, George Githinji, Gert van Zyl, Gordon A. Awandare, Grit Schubert, Gugu P. Maphalala, Hafaliana C. Ranaivoson, Hajar Lemriss, Happi Anise, Haruka Abe, Hela H. Karray, Hellen Nansumba, Hesham A. Elgahzaly, Hlanai Gumbo, Ibtihel Smeti, Ikhlas B. Ayed, Ikponmwosa Odia, Ilhem Boutiba Ben Boubaker, Imed Gaaloul, Inbal Gazy, Innocent Mudau, Isaac Ssewanyana, Iyaloo Konstantinus, Jean B. Lekana-Douk, Jean-Claude C. Makangara, Jean-Jacques M. Tamfum, Jean-Michel Heraud, Jeffrey G. Shaffer, Jennifer Giandhari, Jingjing Li, Jiro Yasuda, Joana Q. Mends, Jocelyn Kiconco, John M. Morobe, John O. Gyapong, Johnson C. Okolie, John T. Kayiwa, Johnathan A. Edwards, Jones Gyamfi, Jouali Farah, Joweria Nakaseegu, Joyce M. Ngoi, Joyce Namulondo, Julia C. Andeko, Julius J. Lutwama, Justin O’Grady, Katherine Siddle, Kayode T. Adeyemi, Kefentse A. Tumedi, Khadija M. Said, Kim Hae-Young, Kwabena O. Duedu, Lahcen Belyamani, Lamia Fki-Berrajah, Lavanya Singh, Leonardo de O. Martins, Lynn Tyers, Magalutcheemee Ramuth, Maha Mastouri, Mahjoub Aouni, Mahmoud el Hefnawi, Maitshwarelo I. Matsheka, Malebogo Kebabonye, Mamadou Diop, Manel Turki, Marietou Paye, Martin M. Nyaga, Mathabo Mareka, Matoke-Muhia Damaris, Maureen W. Mburu, Maximillian Mpina, Mba Nwando, Michael Owusu, Michael R. Wiley, Mirabeau T. Youtchou, Mitoha O. Ayekaba, Mohamed Abouelhoda, Mohamed G. Seadawy, Mohamed K. Khalifa, Mooko Sekhele, Mouna Ouadghiri, Moussa M. Diagne, Mulenga Mwenda, Mushal Allam, My V. T. Phan, Nabil Abid, Nadia Touil, Nadine Rujeni, Najla Kharrat, Nalia Ismael, Ndongo Dia, Nedio Mabunda, Nei-yuan Hsiao, Nelson B. Silochi, Ngoy Nsenga, Nicksy Gumede, Nicola Mulder, Nnaemeka Ndodo, Norosoa H Razanajatovo, Nosamiefan Iguosadolo, Oguzie Judith, Ojide C. Kingsley, Okogbenin Sylvanus, Okokhere Peter, Oladiji Femi, Olawoye Idowu, Olumade Testimony, Omoruyi E. Chukwuma, Onwe E. Ogah, Chika K. Onwuamah, Oshomah Cyril, Ousmane Faye, Oyewale Tomori, Pascale Ondoa, Patrice Combe, Patrick Semanda, Paul E. Oluniyi, Paulo Arnaldo, Peter K. Quashie, Philippe Dussart, Phillip A. Bester, Placide K. Mbala, Reuben Ayivor-Djanie, Richard Njouom, Richard O. Phillips, Richmond Gorman, Robert A. Kingsley, Rosina A. A. Carr, Saâd El Kabbaj, Saba Gargouri, Saber Masmoudi, Safietou Sankhe, Salako B. Lawal, Samar Kassim, Sameh Trabelsi, Samar Metha, Sami Kammoun, Sanaâ Lemriss, Sara H. A. Agwa, Sébastien Calvignac-Spencer, Stephen F. Schaffner, Seydou Doumbia, Sheila M. Mandanda, Sherihane Aryeetey, Shymaa S. Ahmed, Siham Elhamoumi, Soafy Andriamandimby, Sobajo Tope, Sonia Lekana-Douki, Sophie Prosolek, Soumeya Ouangraoua, Steve A. Mundeke, Steven Rudder, Sumir Panji, Sureshnee Pillay, Susan Engelbrecht, Susan Nabadda, Sylvie Behillil, Sylvie L. Budiaki, Sylvie van der Werf, Tapfumanei Mashe, Tarik Aanniz, Thabo Mohale, Thanh Le-Viet, Tobias Schindler, Ugochukwu J. Anyaneji, Ugwu Chinedu, Upasana Ramphal, Uwanibe Jessica, Uwem George, Vagner Fonseca, Vincent Enouf, Vivianne Gorova, Wael H. Roshdy, William K. Ampofo, Wolfgang Preiser, Wonderful T. Choga, Yaw Bediako, Yeshnee Naidoo, Yvan Butera, Zaydah R. de Laurent, Amadou A. Sall, Ahmed Rebai, Anne von Gottberg, Bourema Kouriba, Carolyn Williamson, Daniel J. Bridges, Ihekweazu Chikwe, Jinal N. Bhiman, Madisa Mine, Matthew Cotten, Sikhulile Moyo, Simani Gaseitsiwe, Ngonda Saasa, Pardis C. Sabeti, Pontiano Kaleebu, Yenew K. Tebeje, Sofonias K. Tessema, Christian Happi, John Nkengasong, Tulio de Oliveira

**Affiliations:** 1 KwaZulu-Natal Research Innovation and Sequencing Platform (KRISP), Nelson R Mandela School of Medicine, University of KwaZulu-Natal, Durban, South Africa.; 2 Centre for Epidemic Response and Innovation (CERI), School of Data Science and Computational Thinking, Stellenbosch University, Stellenbosch, South Africa.; 3 Laboratorio de Flavivirus, Fundacao Oswaldo Cruz, Rio de Janeiro, Brazil.; 4 Laboratório de Genética Celular e Molecular, Universidade Federal de Minas Gerais, Belo Horizonte, Minas Gerais, Brazil.; 5 Department of Geography and GIS, University of Cincinnati, Cincinnati, OH, USA.; 6 Institute of Infectious Diseases and Molecular Medicine, Department of Integrative Biomedical Sciences, Computational Biology Division, University of Cape Town, Cape Town, South Africa.; 7 Division of Medical Virology, Wellcome Centre for Infectious Diseases in Africa, Institute of Infectious Disease and Molecular Medicine, University of Cape Town, Cape Town, South Africa.; 8 Department of Entomology and Plant Pathology, North Carolina State University, Raleigh, NC, USA.; 9 Bioinformatics Research Center, North Carolina State University, Raleigh, NC, USA.; 10 Cancer Biology Department, Virology and Immunology Unit, National Cancer Institute, Cairo University, Cairo 11796, Egypt.; 11 Centre d’Infectiologie Charles Mérieux-Mali (CICM-Mali), Bamako, Mali.; 12 Bacteriology and Virology Department Souro Sanou University Hospital, Bobo-Dioulasso, Burkina Faso.; 13 MRCG at LSHTM Genomics Lab, Fajara, Gambia.; 14 African Centre of Excellence for Genomics of Infectious Diseases (ACEGID), Redeemer’s University, Ede, Osun State, Nigeria.; 15 Department of Virology, College of Medicine, University of Ibadan, Ibadan, Nigeria.; 16 Department of Medical Microbiology and Parasitology, Faculty of Basic Clinical Sciences, College of Health Sciences, University of Ilorin, Ilorin, Kwara State, Nigeria.; 17 CHU Habib Bourguiba, Laboratory of Microbiology, Faculty of Medicine of sFax, University of sFax, sFax, Tunisia.; 18 Pathogen Sequencing Lab, Institut National de Recherche Biomédicale (INRB), Kinshasa, Democratic Republic of the Congo.; 19 Université de Kinshasa (UNIKIN), Kinshasa, Democratic Republic of the Congo.; 20 Genomics Research Program, Children’s Cancer Hospital, Cairo, Egypt.; 21 Institute of Pathogen Genomics, Africa Centres for Disease Control and Prevention (Africa CDC), Addis Ababa, Ethiopia.; 22 Laboratory of Transmissible Diseases and Biological Active Substances (LR99ES27), Faculty of Pharmacy of Monastir, Monastir, Tunisia.; 23 Laboratory of Microbiology, University Hospital of Monastir, Monastir, Tunisia.; 24 Internal Medicine Department, Alex Ekwueme Federal University Teaching Hospital, Abakaliki, Nigeria.; 25 Irrua Specialist Teaching Hospital, Irrua, Nigeria.; 26 Quadram Institute Bioscience, Norwich, UK.; 27 World Health Organization, Africa Region, Brazzaville Congo.; 28 Centre de Recherche et de Formation en Infectiologie de Guinée (CERFIG), Université de Conakry, Conakry, Guinea.; 29 TransVIHMI, Montpellier University/IRD/INSERM, Montpellier, France.; 30 Virology Department, Institut Pasteur de Dakar, Dakar, Senegal.; 31 Mali-University Clinical Research Center (UCRC), Bamako, Mali.; 32 Laboratory of Molecular and Cellular Screening Processes, Centre of Biotechnology of Sfax, University of Sfax, Sfax, Tunisia.; 33 Broad Insitute of Harvard and MIT, Cambridge, MA, USA.; 34 KEMRI-Wellcome Trust Research Programme/KEMRI-CGMR-C, Kilifi, Kenya.; 35 National Institute for Communicable Diseases (NICD) of the National Health Laboratory Service (NHLS), Johannesburg, South Africa.; 36 The Nigerian Institute of Medical Research, Yaba, Lagos, Nigeria; 37 Institute of Virology, Charité – Universitätsmedizin, Berlin, Germany.; 38 Medical Biotechnology Laboratory, Rabat Medical and Pharmacy School, Mohammed V University, Rabat, Morocco.; 39 Department of Immunology, University of Maiduguri Teaching Hospital, P.M.B. 1414, Maiduguri, Nigeria.; 40 MRC/UVRI and LSHTM Uganda Research Unit, Entebbe, Uganda.; 41 Division of Medical Virology, Faculty of Medicine and Health Sciences, Stellenbosch University, Tygerberg, Cape Town, South Africa.; 42 The Biotechnology Center of the University of Yaoundé I, Cameroon and CDC Foundation, Yaounde, Cameroon.; 43 Department of Ecology and Evolution, University of Chicago, Chicago, IL, USA.; 44 Virology Unit, Institut Pasteur de Madagascar, Antananarivo, Madagascar.; 45 University of Nebraska Medical Center (UNMC), Omaha, NE, USA.; 46 Antibody Immunity Research Unit, School of Pathology, University of the Witwatersrand, Johannesburg, South Africa.; 47 CHU de Bouaké, Laboratoire/Unité de Diagnostic des Virus des Fièvres Hémorragiques et Virus Émergents, Bouaké, Côte d’Ivoire.; 48 School of Public Health, Pwani University, Kilifi, Kenya.; 49 Swiss Tropical and Public Health Institute, Basel, Switzerland.; 50 West African Centre for Cell Biology of Infectious Pathogens (WACCBIP), Department of Biochemistry, Cell and Molecular Biology, University of Ghana, Accra, Ghana.; 51 School of Life Sciences and Zeeman Institute for Systems Biology and Infectious Disease Epidemiology Research (SBIDER), University of Warwick, Coventry, UK.; 52 Uganda Virus Research Institute, Entebbe, Uganda.; 53 Division of Virology, National Health Laboratory Service and University of the Free State, Bloemfontein, South Africa.; 54 Botswana Harvard AIDS Institute Partnership and Botswana Harvard HIV Reference Laboratory, Gaborone, Botswana.; 55 University of Zambia, School of Veterinary Medicine, Department of Disease Control, Lusaka, Zambia.; 56 African Society for Laboratory Medicine, Addis Ababa, Ethiopia.; 57 Functional Genomic Platform/National Centre for Scientific and Technical Research (CNRST), Rabat, Morocco.; 58 Rwanda National Reference Laboratory, Kigali, Rwanda.; 59 Robert Koch-Institute, Berlin, Germany.; 60 G5 Evolutionary Genomics of RNA Viruses, Institut Pasteur, Paris, France.; 61 Research Unit of Autoimmune Diseases UR17DN02, Military Hospital of Tunis, University of Tunis El Manar, Tunis, Tunisia.; 62 Department of Public Health, Ministry of Health, Ilorin, Kwara State, Nigeria.; 63 Laboratory of Clinical Virology, Institut Pasteur de Tunis, Tunis, Tunisia.; 64 Faculty of Pharmacy of Monastir, Monastir, Tunisia.; 65 National Microbiology Reference Laboratory, Harare, Zimbabwe.; 66 National Influenza Centre, Viral Respiratory Laboratory, Algiers, Algeria.; 67 Medical Microbiology and Parasitology Department, College of Medicine, University of Ibadan, Ibadan, Nigeria.; 68 Lincoln International Institute for Rural Health, University of Lincoln, Lincoln, UK.; 69 Centre for the AIDS Programme of Research in South Africa (CAPRISA), Durban, South Africa.; 70 Africa Health Research Institute, KwaZulu-Natal, Durban, South Africa.; 71 Department of Biochemistry and Biotechnology, Pwani University, Kilifi, Kenya.; 72 National Health Laboratory Service (NHLS), Tygerberg, Cape Town, South Africa.; 73 Institution and Department, Ministry Of Health, COVID-19 Testing Laboratory, Mbabane, Kingdom of Eswatini.; 74 Laboratory of Health Sciences and Technologies, High Institute of Health Sciences, Hassan 1st University, Settat, Morocco.; 75 Department of Emerging Infectious Diseases, Institute of Tropical Medicine, Nagasaki University, Nagasaki, Japan.; 76 Central Public Health Laboratories (CPHL), Kampala, Uganda.; 77 Faculty of Medicine Ain Shams Research institute (MASRI), Ain Shams University, Cairo, Egypt.; 78 Charles Nicolle Hospital, Laboratory of Microbiology, National Influenza Center, 1006 Tunis, Tunisia.; 79 Laboratory of Transmissible Diseases and Biological Active Substances (LR99ES27), Faculty of Pharmacy of Monastir, University of Monastir, Monastir, Tunisia.; 80 Department of Biochemistry and Molecular Biology, The Institute for Medical Research Israel-Canada, Hadassah Medical School, The Hebrew University of Jerusalem, Jerusalem, Israel.; 81 Namibia Institute of Pathology, Windhoek, Namibia.; 82 Centre Interdisciplinaires de Recherches Medicales de Franceville (CIRMF), Franceville, Gabon.; 83 Department of Biostatistics and Data Science, School of Public Health and Tropical Medicine, Tulane University, New Orleans, LA, USA.; 84 Urban Health Collaborative, Dornsife School of Public Health, Drexel University, Philadelphia, PA, USA.; 85 UHAS COVID-19 Testing and Research Centre, University of Health and Allied Sciences, Ho, Ghana.; 86 Rollins School of Public Health, Emory University, Atlanta, GA, USA.; 87 Anoual Laboratory, Casablanca, Morocco.; 88 Botswana Institute for Technology Research and Innovation, Gaborone, Botswana.; 89 New York University Grossman School of Medicine, New York City, NY, USA.; 90 Centre de Recherches Medicales de Lambarene (CERMEL), Lambarene, Gabon.; 91 Virology/Molecular Biology Department, Central Health Laboratory, Ministry of Health and Wellness, Mauritius.; 92 Center of Scientific Excellence for Influenza Viruses, National Research Centre (NRC), Cairo Egypt.; 93 Ministry of Health and Wellness, Gaborone, Botswana.; 94 Next Generation Sequencing Unit and Division of Virology, Faculty of Health Sciences, University of the Free State, Bloemfontein 9300, South Africa.; 95 National Reference Laboratory Lesotho, Maseru, Lesotho.; 96 Centre for Biotechnology Research and Development, Kenya Medical Research Institute, Nairobi, Kenya.; 97 Laboratorio de Investigaciones de Baney, Baney, Equatorial Guinea.; 98 Ifakara Health Institute, Dar-es-Salaam, Tanzania.; 99 Nigeria Centre for Disease Control, Abuja, Nigeria.; 100 Department of Medical Diagnostics, Kumasi Centre for Collaborative Research in Tropical Medicine, Kwame Nkrumah University of Science and Technology, Kumasi, Ghana.; 101 Department of Medical Laboratory Science, Niger Delta University, Bayelsa State, Nigeria.; 102 Systems and Biomedical Engineering Department, Faculty of Engineering, Cairo University, Cairo 12613, Egypt.; 103 King Faisal Specialist Hospital and Research Center, Riyadh, Kingdom of Saudi Arabia.; 104 Biological Prevention Department, Main Chemical Laboratories, Egypt Army, Cairo, Egypt.; 105 PATH, Lusaka, Zambia.; 106 Department of Biotechnology, High Institute of Biotechnology of Sidi Thabet, University of Manouba, BP-66, 2020 Ariana-Tunis, Tunisia.; 107 Genomic Center for Human Pathologies (GENOPATH), Faculty of Medicine and Pharmacy, Mohammed V University, Rabat, Morocco.; 108 Rwanda National Joint Task Force COVID-19, Rwanda Biomedical Centre, Ministry of Health, Kigali, Rwanda.; 109 School of Health Sciences, College of Medicine and Health Sciences, University of Rwanda, Kigali, Rwanda.; 110 Instituto Nacional de Saude (INS), Maputo, Mozambique.; 111 National Health Laboratory Service (NHLS), Cape Town, South Africa.; 112 Computational Biology Division, Department of Integrative Biomedical Sciences, IDM, CIDRI Africa Wellcome Trust Centre, University of Cape Town, Cape Town, South Africa.; 113 Virology Laboratory, Alex Ekwueme Federal University Teaching Hospital, Abakaliki, Nigeria.; 114 Department of Epidemiology and Community Health, Faculty of Clinical Sciences, College of Health Sciences, University of Ilorin, Ilorin, Kwara State, Nigeria.; 115 Alex Ekwueme Federal University Teaching Hospital, Abakaliki, Nigeria.; 116 Mayotte Hospital Center, Mayotte, France.; 117 Virology Service, Centre Pasteur of Cameroun, Yaounde, Cameroon.; 118 Kumasi Centre for Collaborative Research in Tropical Medicine, Kwame Nkrumah University of Science and Technology, Kumasi, Ghana.; 119 Laboratoire de Recherche et d’Analyses Médicales de la Gendarmerie Royale, Rabat, Morocco.; 120 Clinical and Experimental Pharmacology Lab, LR16SP02, National Center of Pharmacovigilance, University of Tunis El Manar, Tunis, Tunisia.; 121 CHU Hedi Chaker Sfax, Service de Pneumologie, Tunis, Tunisia.; 122 Laboratoire de Recherche et d’Analyses Médicales de la Gendarmerie Royale, Rabat, Morocco.; 123 Central Public Health Laboratories (CPHL), Cairo, Egypt.; 124 Centre MURAZ, Ouagadougou, Burkina Faso.; 125 National Institute of Public Health of Burkina Faso (INSP/BF), Ouagadougou, Burkina Faso.; 126 National Reference Center for Respiratory Viruses, Molecular Genetics of RNA Viruses, UMR 3569 CNRS, University of Paris, Institut Pasteur, Paris, France.; 127 Sub-Saharan African Network For TB/HIV Research Excellence (SANTHE), Durban, South Africa.; 128 Coordenação Geral de Laboratórios de Saúde Pública/Secretaria de Vigilância em Saúde, Ministério da Saúde, Brasília, Distrito Federal, Brazil.; 129 World Health Organization, WHO Lesotho, Maseru, Lesotho.; 130 Med24 Medical Centre, Ruwa, Zimbabwe.; 131 Division of Human Genetics, Department of Pathology, University of Cape Town, Cape Town, South Africa.; 132 Center for Human Genetics, College of Medicine and Health Sciences, University of Rwanda, Kigali, Rwanda.; 133 Laboratory of Human Genetics, GIGA Research Institute, Liège, Belgium.; 134 National Health Laboratory, Gaborone, Botswana.; 135 MRC-University of Glasgow Centre for Virus Research, Glasgow, UK.; 136 Harvard T.H. Chan School of Public Health, Boston, MA, USA.; 137 Department of Global Health, University of Washington, Seattle, WA, USA.; 138 Centre for Human Virology and Genomics, Nigerian Institute of Medical Research, Yaba, Lagos, Nigeria.; 139 School of Pathology, Faculty of Health Science, University of the Witwatersrand, Johannesburg, South Africa.

## Abstract

The progression of the severe acute respiratory syndrome coronavirus 2 (SARS-CoV-2) pandemic in Africa has so far been heterogeneous, and the full impact is not yet well understood. In this study, we describe the genomic epidemiology using a dataset of 8746 genomes from 33 African countries and two overseas territories. We show that the epidemics in most countries were initiated by importations predominantly from Europe, which diminished after the early introduction of international travel restrictions. As the pandemic progressed, ongoing transmission in many countries and increasing mobility led to the emergence and spread within the continent of many variants of concern and interest, such as B.1.351, B.1.525, A.23.1, and C.1.1. Although distorted by low sampling numbers and blind spots, the findings highlight that Africa must not be left behind in the global pandemic response, otherwise it could become a source for new variants.

## SARS-CoV-2 across Africa

The impact of the severe acute respiratory syndrome coronavirus 2 (SARS-CoV-2) pandemic has been hard to track in African countries, largely because of patchy data. Wilkinson *et al*. curated viral genomes collected in 2021 from several countries across the continent. Outbreaks during 2020 in each African country were initiated by imported cases, mostly from Europe. As the pandemic developed, case numbers in African countries were likely many times higher than reported, and subsequent waves of the pandemic appear to have been more severe. Consequently, high-transmission variants have emerged that have spread within the continent, and African countries must be included in global control efforts. —CA

**Figure:**
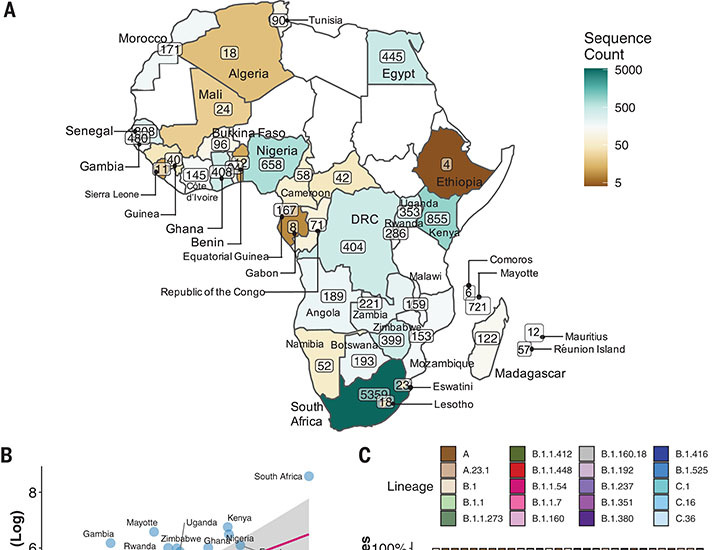

## Teaser

The early phases of the COVID-19 pandemic in Africa were dominated by European imports, followed by the local generation of variants.

Severe acute respiratory syndrome coronavirus 2 (SARS-CoV-2) emerged in late 2019 in Wuhan, China (*1*, *2*). Since then, the virus has spread to all corners of the world, causing almost 150 million cases of COVID-19 and more than 3 million deaths by the end of April 2021. Throughout the pandemic, it has been noted that Africa accounts for a relatively low proportion of reported cases and deaths—by the end of April 2021, there had been ~4.5 million cases and ~120,000 deaths on the continent, corresponding to less than 4% of the global burden. However, emerging data from seroprevalence surveys and autopsy studies in some African countries suggest that the true number of infections and deaths may be severalfold higher than reported (*3*, *4*). In addition, a recent analysis has shown that in many African countries, the second wave of the pandemic was more severe than the first wave (*5*).

The first cases of COVID-19 on the African continent were reported in Nigeria, Egypt, and South Africa between mid-February and early March 2020, and most countries had reported cases by the end of March 2020 (*6*–*8*). These early cases were concentrated among airline travelers returning from regions of the world with high levels of community transmission. Many African countries introduced early public health and social measures, including international travel controls, quarantine for returning travelers, and internal lockdown measures, to limit the spread of the virus and give health services time to prepare (*5*, 9). The initial phase of the epidemic was then heterogeneous, with relatively high case numbers reported in North Africa and southern Africa, and fewer cases reported in other regions.

From the onset of the pandemic, genomic surveillance has been at the forefront of the COVID-19 response in Africa (*10*). Rapid implementation of SARS-CoV-2 sequencing by various laboratories in Africa enabled genomic data to be generated and shared from the early imported cases. In Nigeria, the first genome sequence was released just 3 days after the announcement of the first case (*6*). Similarly, in Uganda, a sequencing program was set up rapidly to facilitate virus tracing, and the collection of samples for sequencing began immediately upon confirmation of the first case (*11*). In South Africa, the Network for Genomic Surveillance in South Africa (NGS-SA) was established in March 2020, and within weeks, genomic analysis was helping to characterize outbreaks and community transmission (*12*).

Genomic surveillance has also been critical for monitoring ongoing SARS-CoV-2 evolution and detection of new SARS-CoV-2 variants in Africa. Intensified sampling by NGS-SA in the Eastern Cape Province of South Africa in November 2020, in response to a rapid resurgence of cases, led to the detection of B.1.351 (501Y.V2) (*13*). This variant was subsequently designated a variant of concern (VOC) by the World Health Organization (WHO), owing to evidence of increased transmissibility (*14*) and resistance to neutralizing antibodies elicited by natural infection and vaccines (*15*–*17*).

In this study, we performed phylogenetic and phylogeographic analyses of SARS-CoV-2 genomic data from 33 African countries and two overseas territories to help characterize the dynamics of the pandemic in Africa. We show that the early introductions were predominantly from Europe, but that as the pandemic progressed, there was increasing spread between African countries. We also describe the emergence and spread of a number of key SARS-CoV-2 variants in Africa and highlight how the spread of B.1.351 (501Y.V2) and other variants contributed to the more severe second wave of the pandemic in many countries.

## SARS-CoV-2 genomic data

**Fig. 1.**
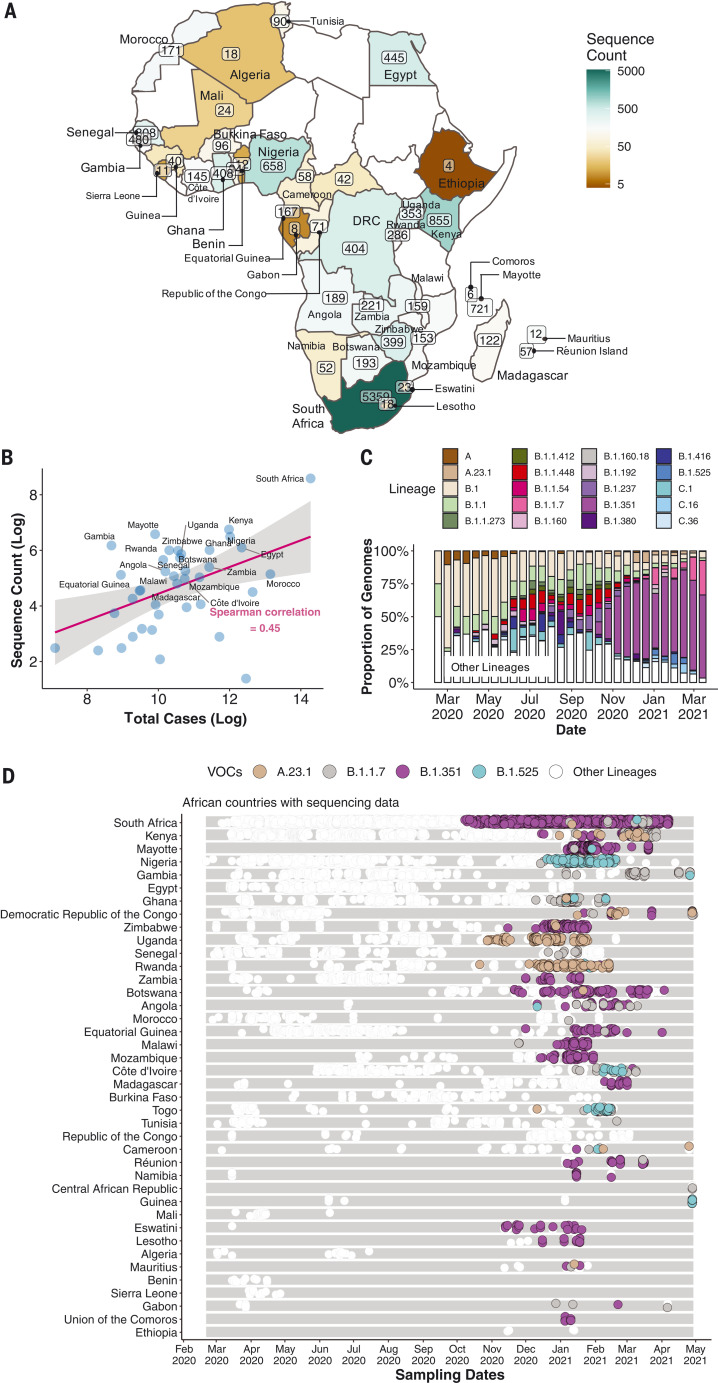
SARS-CoV-2 sequences in Africa. (**A**) Map of the African continent with the number of SARS-CoV-2 sequences reflected in GISAID as of 5 May 2021. (**B**) Regression plot of the number of viral sequences versus the number of reported COVID-19 cases in various African countries as of 5 May 2021. Countries with >500 sequences are labeled. The shaded region indicates the 95% confidence interval. (**C**) Progressive distribution of the top 20 PANGO lineages on the African continent. (**D**) Temporal sampling of SARS-CoV-2 sequences in African countries (ordered by total number of sequences) through time, with VOCs of note highlighted and annotated according to their PANGO lineage assignment.

By 5 May 2021, 14,504 SARS-CoV-2 genomes had been submitted to the GISAID database (*18*) from 38 African countries and two overseas territories (Mayotte and Réunion) (Fig. 1A). Overall, this corresponds to approximately one sequence per ~300 reported cases. Almost half of the sequences were from South Africa (*n* = 5362), consistent with it being responsible for almost half of the reported cases in Africa. Overall, the number of sequences correlates closely with the number of reported cases per country (Fig. 1B). The countries and territories with the highest coverage of sequencing (defined as genomes per reported case) are Kenya (*n* = 856, one sequence per ~203 cases), Mayotte (*n* = 721, one sequence per ~21 cases), and Nigeria (*n* = 660, one sequence per ~250 cases). Although genomic surveillance started early in many countries, few have evidence of consistent sampling across the whole year. Half of all African genomes were deposited in the first 10 weeks of 2021, suggesting intensified surveillance in the second wave after the detection of B.1.351 (501Y.V2) and other variants (Fig. 1, C and D).

## Genetic diversity and lineage dynamics in Africa

**Fig. 2.**
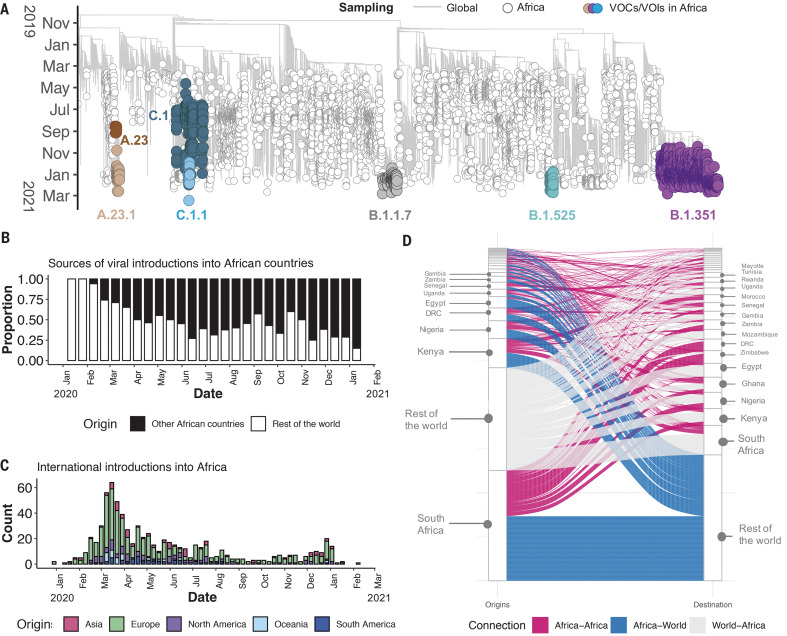
Phylogenetic reconstruction of the SARS-CoV-2 pandemic on the continent of Africa. (**A**) Time-resolved maximum likelihood tree containing 8746 high-quality African SARS-CoV-2 near-full-genome sequences analyzed against a backdrop of global reference sequences. VOIs and VOCs are highlighted on the phylogeny. (**B**) Sources of viral introductions into African countries characterized as external introductions from the rest of the world versus internal introductions from other African countries. (**C**) Total external viral introductions over time into Africa. (**D**) The number of viral imports and exports into and out of various African countries depicted as internal (between African countries, in pink) or external (between African and non-African countries, in blue and gray).

Of the 10,326 genomes retrieved from GISAID by the end of March 2021, 8746 genomes passed quality control and met the minimum metadata requirements. These genomes from Africa were compared in a phylogenetic framework with 11,891 representative genomes from around the world. Ancestral location state reconstruction of the dated phylogeny (hereafter referred to as discrete phylogeographic reconstruction) allowed us to infer the number of viral imports and exports between Africa and the rest of the world, and between individual African countries. African genomes in this study spanned the whole global genetic diversity of SARS-CoV-2, a pattern that largely reflects multiple introductions over time from the rest of the world (Fig. 2A).

In total, we detected at least 757 [95% confidence interval (CI): 728 to 786] viral introductions into African countries between the start of 2020 and February 2021, more than half of which occurred before the end of May 2020. Although the early phase of the pandemic was dominated by importations from outside Africa, predominantly from Europe, there was then a shift in the dynamics, with an increasing number of importations from other African countries as the pandemic progressed (Fig. 2, B and C). A rarefaction analysis in which we systematically subsampled genomes shows that vastly more introductions would have likely been identified with increased sampling in Africa or globally, suggesting that the introductions we identified are really just the “ears of the hippo,” or a small part of a larger problem (fig. S1).

South Africa, Kenya, and Nigeria appear as major sources of importations into other African countries (Fig. 2D), although this is likely to be influenced by these three countries having the greatest number of deposited sequences. Particularly notable is the southern African region, where South Africa is the source for a large proportion (~80%) of the importations to other countries in the region. The North African region demonstrates a different pattern to the rest of the continent, with more viral introductions from Europe and Asia (particularly the Middle East) than from other African countries (fig. S2).

Africa has also contributed to the international spread of the virus, with at least 324 (95% CI: 295 to 353) exportation events from Africa to the rest of the world detected in this dataset. Consistent with the source of importations, most exports were to Europe (41%), Asia (26%), and North America (14%). As with the number of importations, exports were relatively evenly distributed over the 1-year period (fig. S3). However, an increase in the number of exportation events occurred between December 2020 and March 2021, which coincided with the second wave of infections in Africa and with some relaxations of travel restrictions around the world.

The early phase of the pandemic was characterized by the predominance of lineage B.1. This was introduced multiple times to African countries and has been detected in all but one of the countries included in this analysis. After its emergence in South Africa, B.1.351 became the most frequently detected SARS-CoV-2 lineage found in Africa (*n* = 1769, ~20%) (Fig. 1C). It was first sampled on 8 October 2020 in South Africa (*13*) and has since spread to 20 other African countries.

As air travel came to an almost complete halt in March and April 2020, the number(s) of detectable viral imports into Africa decreased and the pandemic entered a phase that was characterized in sub-Saharan Africa by sustained low levels of within-country movements and occasional international viral movements between neighboring countries, presumably via road and rail links between these. Though some border posts between countries were closed during the initial lockdown period (table S1), others remained open to allow trade to continue. Regional trade in southern Africa was only slightly affected by lockdown restrictions and quickly rebounded to prepandemic levels (fig. S4) after the relaxation of restrictions between June 2020 and December 2020.

Although lineage A viruses were imported into several African countries, they only account for 1.3% of genomes sampled in Africa. Despite lineage A viruses initially causing many localized clustered outbreaks, each the result of independent introductions to several countries (e.g., Burkina Faso, Côte d’Ivoire, and Nigeria), they were later largely replaced by lineage B viruses as the pandemic evolved. This is possibly due to the increased transmissibility of lineage B viruses by virtue of the D614G (Asp^614^→Gly) mutation in the spike protein (*19*, *20*). However, there is evidence of an increasing prevalence of lineage A viruses in some African countries (*11*). In particular, A.23.1 emerged in East Africa and appears to be rapidly increasing in prevalence in Uganda and Rwanda (*11*). Furthermore, a highly divergent variant from lineage A was recently identified in Angola from individuals arriving from Tanzania (*21*).

## Emergence and spread of new SARS-CoV-2 variants

**Fig. 3.**
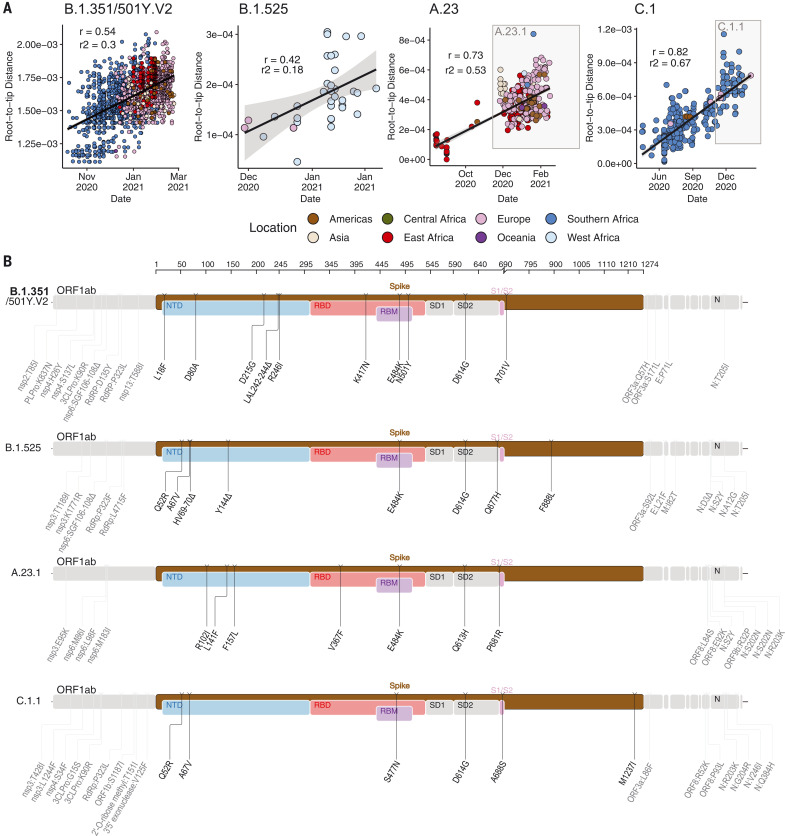
Genetic profile of VOCs and VOIs under investigation. (**A**) Root-to-tip regression plots for four lineages of interest. C.1 and A.23 show continued evolution into VOIs C.1.1 and A.23.1, respectively. *r*, coefficient of correlation; *r*^2^, coefficient of determination. (**B**) Genome maps of four VOCs and VOIs, where the spike region is shown in detail and in color and the rest of the genome is shown in gray. ORF, open reading frame; NTD, N-terminal domain; RBD, receptor binding domain; RBM, receptor binding motif; SD1, subdomain 1; SD2, subdomain 2.

To determine how some of the key SARS-CoV-2 variants are spreading within Africa, we performed phylogeographic analyses on the VOC B.1.351, the variant of interest (VOI) B.1.525, and two additional variants that emerged and that we designated as VOIs for this analysis (A.23.1 and C.1.1). These African VOCs and VOIs have multiple mutations on the spike glycoprotein, and a molecular clock analysis of these four datasets provided strong evidence that these four lineages are evolving in a clock-like manner (Fig. 3, A and B).

**Fig. 4.**
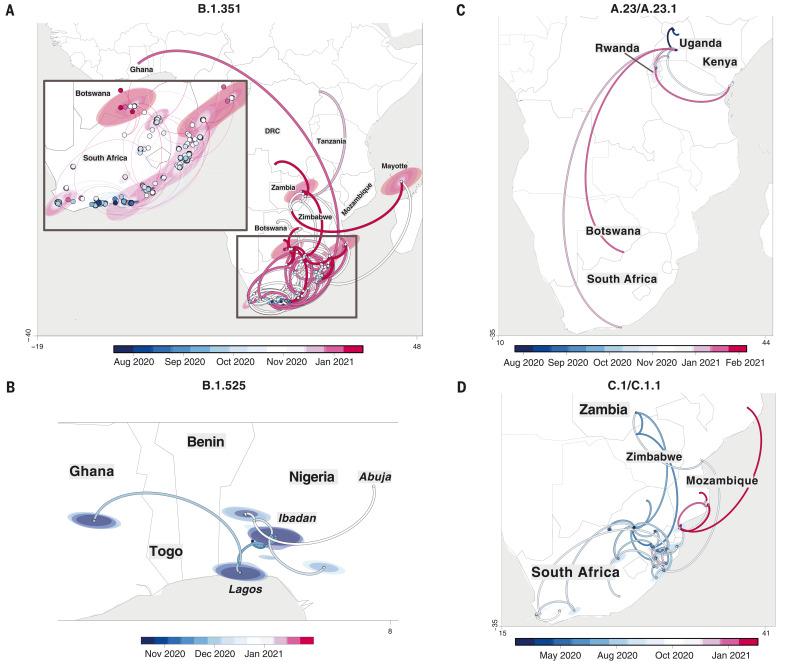
Phylogeographic reconstruction of the spread of four VOCs and VOIs across the African continent. (**A** to **D**) Phylogeographic reconstruction of the spread of four VOCs and VOIs across the African continent using sequences showing strict continuous transmission across geographical regions: B.1.351 (A), B.1.525 (B), A.23/A.23.1 (C), and C.1/C.1.1 (D). Curved lines denote the direction of transmission in the counterclockwise direction. Solid lines show transmission paths as inferred by phylogeographic reconstruction and colored by date, whereas dashed lines show the known travel history of the particular case considered.

B.1.351 was first sampled in South Africa in October 2020, but phylogeographic analysis suggests that it emerged earlier, around August 2020. It is defined by 10 mutations in the spike protein, including K417N (Lys^417^→Asn), E484K (Glu^484^→Lys), and N501Y (Asn^501^→Tyr) in the receptor binding domain (Fig. 3B). After its emergence in the Eastern Cape, it spread extensively within South Africa (Fig. 4A). By November 2020, the variant had spread into neighboring Botswana and Mozambique, and by December 2020, it had reached Zambia and Mayotte. Within the first 3 months of 2021, further exports from South Africa into Botswana, Zimbabwe, Mozambique, and Zambia occurred. By March 2021, B.1.351 had become the dominant lineage within most southern African countries as well as the overseas territories of Mayotte and Réunion (fig. S5). Our phylogeographic reconstruction also demonstrates movement of B.1.351 into East and Central Africa directly from southern Africa. Our discrete phylogeographic analysis of a wider sample of B.1.351 isolates demonstrates the spread of the lineage into West Africa. This patient from West Africa had a known travel history to Europe, so it is possible that the patient acquired the infection while in Europe or in transit and not from other African sources (fig. S6).

B.1.525 is a VOI defined by six substitutions in the spike protein [Q52R (Gln^52^→Arg), A67V (Ala^67^→Val), E484K, D614G, Q677H (Gln^677^→His), and F888L (Phe^888^→Leu)] and two deletions in the N-terminal domain [HV69-70Δ (deletion of His and Val at positions 69 and 70) and Y144Δ (deletion of Tyr at position 144)]. This was first sampled in the United Kingdom in mid-December 2020, but our phylogeographic reconstruction suggests that the variant originated in Nigeria in November 2020 [95% highest posterior density (HPD) 2020-11-01 to 2020-12-03] (Fig. 4B). Since then, it has spread throughout much of Nigeria and neighboring Ghana. Given sparse sampling from other neighboring countries within West and Central Africa (Fig. 1, A and C), the extent of the spread of this VOI in the region is not clear. Beyond Africa, this VOI has spread to Europe and the United States (fig. S6).

We designated A.23.1 and C.1.1 as VOIs for the purposes of this analysis because they present good examples of the continued evolution of the virus within Africa (*11*, *13*). Lineage A.23, characterized by three spike mutations [F157L (Phe^157^→Leu), V367F (Val^367^→Phe), and Q613H (Gln^613^→His)], was first detected in a Ugandan prison in Amuru in July 2020 (95% HPD: 2020-07-15 to 2020-08-02). From there, the lineage was transmitted to Kitgum prison, possibly facilitated by the transfer of prisoners. Subsequently, the A.23 lineage spilled into the general population and spread to Kampala, adding other spike mutations [R102I (Arg^102^→Ile), L141F (Leu^141^→Phe), E484K, and P681R (Pro^681^→Arg)] along with additional mutations in nsp3, nsp6, ORF8, and ORF9, prompting a new lineage classification, A.23.1 (Fig. 3, A and B). Since the emergence of A.23.1 in September 2020 (95% HPD: 2020-09-02 to 2020-09-28), it has spread regionally into neighboring Rwanda and Kenya and has now also reached South Africa and Botswana in the south and Ghana in the west (Fig. 4C). However, our phylogeographic reconstruction of A.23.1 suggests that the introduction into Ghana may have occurred via Europe (fig. S6), whereas the introductions into southern Africa likely occurred directly from East Africa. This is consistent with epidemiological data suggesting that the case detected in South Africa was a contact of an individual who had recently traveled to Kenya.

Lineage C.1 emerged in South Africa in March 2020 (95% HPD: 2020-03-13 to 2020-04-17) during a cluster outbreak before the first wave of the epidemic (*13*). C.1.1 is defined by the spike mutations S477N (Ser^477^→Asn), A688S (Ala^688^→Ser), and M1237I (Met^1237^→Ile) and also contains the Q52R and A67V mutations similar to B.1.525 (Fig. 3B). A continuous trait phylogeographic reconstruction of the movement dynamics of these lineages suggests that C.1 emerged in the city of Johannesburg and spread within South Africa during the first wave (Fig. 4D). Independent exports of C.1 from South Africa led to regional spread to Zambia (June to July 2020) and Mozambique (July to August 2020), and the evolution to C.1.1 seems to have occurred in Mozambique around mid-September 2020 (95% HPD: 2020-09-07 to 2020-10-05). An in-depth analysis of SARS-CoV-2 genotypes from Mozambique suggests that the C.1.1 lineage was the most prevalent in the country until the introduction of B.1.351, which has dominated the epidemic since (fig. S5).

The VOC B.1.1.7, which was first sampled in Kent, England, in September 2020 (*22*), has also increased in prevalence in several African countries (fig. S5). To date, this VOC has been detected in 11 African countries, as well as the Indian Ocean islands of Mauritius and Mayotte (fig. S7). The time-resolved phylogeny suggests that this lineage was introduced into Africa on at least 16 occasions between November 2020 and February 2021, with evidence of local transmission in Nigeria and Ghana.

## Conclusions

Our phylogeographic reconstruction of past viral dissemination patterns suggests a strong epidemiological linkage between Europe and Africa, with 64% of detectable viral imports into Africa originating in Europe and 41% of detectable viral exports from Africa landing in Europe (Fig. 1C). This phylogeographic analysis also suggests a changing pattern of viral diffusion into and within Africa over the course of 2020. In almost all instances, the earliest introductions of SARS-CoV-2 into individual African countries were from countries outside Africa.

High rates of COVID-19 testing and consistent genomic surveillance in the south of the continent have led to the early identification of VOCs such as B.1.351 and VOIs such as C.1.1 (*13*). Since the discovery of these southern African variants, several other SARS-CoV-2 VOIs have emerged in different parts of the world, including elsewhere on the African continent, such as B.1.525 in West Africa and A.23.1 in East Africa. There is strong evidence that both of these VOIs are rising in frequency in the regions where they have been detected, which suggests that they may possess higher fitness than other variants in these regions. Although more-focused research on the biological properties of these VOIs is needed to confirm whether they should be considered VOCs, it would be prudent to assume the worst and focus on limiting their spread. It will be important to investigate how these different variants compete against one another if they occupy the same region.

Our focused phylogenetic analysis of the B.1.351 lineage revealed that in the final months of 2020, this variant spread from South Africa into neighboring countries, reaching as far north as the Democratic Republic of the Congo (DRC) by February 2021. This spread may have been facilitated through rail and road networks that form major transport arteries linking South Africa’s ocean ports to commercial and industrial centres in Botswana, Zimbabwe, Zambia, and the southern parts of the DRC. The rapid, apparently unimpeded spread of B.1.351 into these countries suggests that current land-border controls that are intended to curb the international spread of the virus are ineffective. Perhaps targeted testing of cross-border travelers, genotyping of positive cases, and the focused tracking of frequent cross-border travelers, such as long distance truckers, would more effectively contain the spread of future VOCs and VOIs that emerge within this region.

The dominance of VOIs and VOCs in Africa has important implications for vaccine rollouts on the continent. For one, slow rollout of vaccines in most African countries creates an environment in which the virus can replicate and evolve: This will almost certainly produce additional VOCs, any of which could derail the global fight against COVID-19. Conversely, with the already widespread presence of known variants, difficult decisions about balancing reduced efficacy and availability of vaccines have to be made. This also highlights how crucial it is that trials are done. From a public health perspective, genomic surveillance is only one item in the toolkit of pandemic preparedness. It is important that such work is closely followed by genotype-to-phenotype research to determine the actual relevance of continued evolution of SARS-CoV-2 and other emerging pathogens.

The rollout of vaccines across Africa has been painfully slow (figs. S8 and S9). There have, however, been notable successes that suggest that the situation is not hopeless. The small island nation of the Seychelles had vaccinated 70% of its population by May 2021. Morocco has kept pace with many developed nations and, by mid-March, had vaccinated ~16% of its population. Rwanda, one of Africa’s most resource-constrained countries, had, within 3 weeks of obtaining its first vaccine doses in early March, managed to provide first doses to ~2.5% of its population. For all other African countries, at the time of writing, vaccine coverage (first dose) was <1.0% of the general population.

The effectiveness of molecular surveillance as a tool for monitoring pandemics is largely dependent on continuous and consistent sampling through time, rapid virus genome sequencing, and rapid reporting. When this is achieved, molecular surveillance can ensure the early detection of changing pandemic characteristics. Further, when such changes are discovered, molecular surveillance data can also guide public health responses. In this regard, the molecular surveillance data that are being gathered by most African countries are less useful than they could be. For example, the time lag between when virus samples are taken and when sequences for these samples are deposited in sequence repositories is so great in some cases that the primary utility of genomic surveillance data is lost (fig. S10). This lag is driven by several factors, depending on the laboratory or country in question: (i) lack of reagents owing to disruptions in global supply chains, (ii) lack of equipment and infrastructure within the originating country, (iii) scarcity of technical skills in laboratory methods or bioinformatic support, and (iv) hesitancy by some health officials to release data. More-recent sampling and prompt reporting is crucial to reveal the genetic characteristics of currently circulating viruses in these countries.

The patchiness of African genomic surveillance data is therefore the main weakness of our study. However, there is evidence that the situation is improving, with ~50% of African SARS-CoV-2 genome sequences having been submitted to the GISAID database within the first 10 weeks of 2021. Although the precise factors underlying this surge in sequencing efforts are unclear, an important driver is almost certainly increased global interest in genomic surveillance after the discovery of multiple VOCs and VOIs since December 2020. We cannot reject that the observed increase in exports from Africa may be due to intensified sequencing activity after the detection of variants around the world. It is important to note here that phylogeographic reconstruction of viral spread is highly dependent on sampling where there is the caveat that the exact routes of viral movements between countries cannot be inferred if there is no sampling in connecting countries. Furthermore, our efforts to reconstruct the movement dynamics of SARS-CoV-2 across the continent are almost certainly biased by uneven sampling between different African countries. It is not a coincidence that we identified South Africa, Kenya, and Nigeria, which have sampled and sequenced the most SARS-CoV-2 genomes, as major sources of viral transmissions between sub-Saharan African countries. However, these countries also had the highest number of infections, which may decrease the sampling biases (Fig. 1A).

The reliability of genomic surveillance as a tool to prevent the emergence and spread of dangerous variants is dependent on the intensity with which it is embraced by national public health programs. As with most other parts of the world, the success of genomic surveillance in Africa requires that more samples are tested for COVID-19, higher proportions of positive samples are sequenced within days of sampling, and persistent analyses of these sequences are performed for concerning signals such as (i) the presence of novel nonsynonymous mutations at genomic sites associated with pathogenicity and immunogenicity, (ii) evidence of positive selection at codon sites where nonsynonymous mutations are observed, and (iii) evidence of lineage expansions. Despite limited sampling, Africa has identified many of the VOCs and VOIs that are being transmitted across the world. Detailed characterization of the variants and their impact on vaccine-induced immunity is of extreme importance. If the pandemic is not controlled in Africa, we may see the production of vaccine escape variants that may profoundly affect the population in Africa and across the world.

## Supplementary Material

20210909-1

## Supplementary Materials

### This PDF file includes:

Materials and Methods Figs. S1 to S10 Tables S1 to S3 References (*24*–*38*)

### Other Supplementary Material for this manuscript includes the following:

Table S4

MDAR Reproducibility Checklist

## Notes

View/request a protocol for this paper from *Bio-protocol*.
