# Supplementary material for: A year of genomic surveillance reveals how the SARS-CoV-2 pandemic unfolded in Africa
Source: Science. 2021 Sep 9;374(6566):423–31. doi: 10.1126/science.abj4336 (PMC7613315; doi:10.1126/science.abj4336)
Supplement: 20210909-1 [file science.abj4336.v1.pdf]

Cite as: E. Wilkinson *et al*., *Science*
10.1126/science.abj4336 (2021).

A year of genomic surveillance reveals how the SARS-CoV-2 pandemic unfolded in Africa

Eduan Wilkinson[1,2]†, Marta Giovanetti[3,4]†, Houriiyah Tegally[1]†, James E. San[1]†, Richard Lessells[1], Diego Cuadros[5], Darren P. Martin[6,7], David A. Rasmussen[8,9], Abdel-Rahman N. Zekri[10], Abdoul K. Sangare[11], Abdoul-Salam Ouedraogo[12], Abdul K. Sesay[13], Abechi Priscilla[14], Adedotun-Sulaiman Kemi[14], Adewunmi M. Olubusuyi[15], Adeyemi O. O. Oluwapelumi[16], Adnène Hammami[17], Adrienne A. Amuri[18,19], Ahmad Sayed[20], Ahmed E. O. Ouma[21], Aida Elargoubi[22,23], Ajayi N. Anthony[24], Ajogbasile F. Victoria[14], Akano Kazeem[14], Akpede George[25], Alexander J. Trotter[26], Ali A. Yahaya[27], Alpha K. Keita[28,29], Amadou Diallo[30], Amadou Kone[31], Amal Souissi[32], Amel Chtourou[17], Ana V. Gutierrez[26], Andrew J. Page[26], Anika Vinze[33], Arash Iranzadeh[6,7], Arnold Lambisia[34], Arshad Ismail[35], Audu Rosemary[36], Augustina Sylverken[37], Ayoade Femi[14], Azeddine Ibrahimi[38], Baba Marycelin[39], Bamidele S. Oderinde[39], Bankole Bolajoko[14], Beatrice Dhaala[40], Belinda L. Herring[27], Berthe-Marie Njanpop-Lafourcade[27], Bronwyn Kleinhans[41], Bronwyn McInnis[10], Bryan Tegomoh[42], Cara Brook[43,44], Catherine B. Pratt[45], Cathrine Scheepers[34,46], Chantal G. Akoua-Koffi[47], Charles N. Agoti[34,48], Christophe Peyrefitte[30], Claudia Daubenberger[49], Collins M. Morang'a[50], D. James Nokes[31,51], Daniel G. Amoako[35], Daniel L. Bugembe[40], Danny Park[33], David Baker[26], Deelan Doolabh[7], Deogratius Ssemwanga[40,52], Derek Tshiabuila[1], Diarra Bassirou[30], Dominic S. Y. Amuzu[50], Dominique Goedhals[53], Donwilliams O. Omuoyo[34], Dorcas Maruapula[54], Ebenezer Foster-Nyarko[26], Eddy K. Lusamaki[18,19], Edgar Simulundu[55], Edidah M. Ong'era[34], Edith N. Ngabana[18,19], Edwin Shumba[56], Elmostafa El Fahime[57], Emmanuel Lokilo[18], Enatha Mukantwari[58], Eromon Philomena[14], Essia Belarbi[59], Etienne Simon-Loriere[60], Etilé A. Anoh[47], Fabian Leendertz[59], Faida Ajili[61], Fakayode O. Enoch[62], Fares Wasfi[63], Fatma Abdelmoula[32,64], Fausta S. Mosha[27], Faustinos T. Takawira[65], Fawzi Derrar[66], Feriel Bouzid[32], Folarin Onikepe[14], Fowotade Adeola[67], Francisca M. Muyembe[18,19], Frank Tanser[68,69,70], Fred A. Dratibi[27], Gabriel K. Mbunsu[19], Gaetan Thilliez[26], Gemma L. Kay[26], George Githinji[34,71], Gert van Zyl[41,72], Gordon A. Awandare[50], Grit Schubert[59], Gugu P. Maphalala[73], Hafaliana C. Ranaivoson[44], Hajar Lemriss[74], Happi Anise[14], Haruka Abe[75], Hela H. Karray[17], Hellen Nansumba[76], Hesham A. Elgahzaly[77], Hlanai Gumbo[65], Ibtihel Smeti[32], Ikhlas B. Ayed[32], Ikponmwosa Odia[25], Ilhem Boutiba-Ben Boubaker[78,79], Imed Gaaloul[22], Inbal Gazy[80], Innocent Mudau[7], Isaac Ssewanyana[76], Iyaloo Konstantinus[81], Jean B. Lekana-Douk[82], Jean-Claude C. Makangara[18,19], Jean-Jacques M. Tamfum[18,19], Jean-Michel Heraud[30,44], Jeffrey G. Shaffer[83], Jennifer Giandhari[1], Jingjing Li[84], Jiro Yasuda[75], Joana Q. Mends[85], Jocelyn Kiconco[52], John M. Morobe[34], John O. Gyapong[85], Johnson C. Okolie[14], John T. Kayiwa[40], Johnathan A. Edwards[68,86], Jones Gyamfi[85], Jouali Farah[87], Joweria Nakaseegu[52], Joyce M. Ngoi[50], Joyce Namulondo[52], Julia C. Andeko[82], Julius J. Lutwama[40], Justin O'Grady[26], Katherine Siddle[33], Kayode T. Adeyemi[14], Kefentse A. Tumedi[88], Khadija M. Said[34], Kim Hae-Young[89], Kwabena O. Duedu[90], Lahcen Belyamani[38], Lamia Fki-Berrajah[17], Lavanya Singh[1], Leonardo de O. Martins[16], Lynn Tyers[7], Magalutcheemee Ramuth[91], Maha Mastouri[22,23], Mahjoub Aouni[22], Mahmoud el Hefnawi[92], Maitshwarelo I. Matsheka[88], Malebogo Kebabonye[93], Mamadou Diop[30], Manel Turki[32], Marietou Paye[33], Martin M. Nyaga[94], Mathabo Mareka[95], Matoke-Muhia Damaris[96], Maureen W. Mburu[34], Maximillian Mpina[49,97,98], Mba Nwando[99], Michael Owusu[100], Michael R. Wiley[45], Mirabeau T. Youtchou[101], Mitoha O. Ayekaba[97], Mohamed Abouelhoda[102,103], Mohamed G. Seadawy[104], Mohamed K. Khalifa[20], Mooko Sekhele[95], Mouna Ouadghiri[38], Moussa M. Diagne[30], Mulenga Mwenda[105], Mushal Allam[35], My V. T. Phan[40], Nabil Abid[79,106], Nadia Touil[107], Nadine Rujeni[108,109], Najla Kharrat[32], Nalia Ismael[110], Ndongo Dia[30], Nedio Mabunda[110], Nei-yuan Hsiao[7,111], Nelson B. Silochi[97], Ngoy Nsenga[27], Nicksy Gumede[27], Nicola Mulder[112], Nnaemeka Ndodo[99], Norosoa H Razanajatovo[44], Nosamiefan Iguosadolo[14], Oguzie Judith[14], Ojide C. Kingsley[113], Okogbenin Sylvanus[25], Okokhere Peter[25], Oladiji Femi[114], Olawoye Idowu[14], Olumade Testimony[14], Omoruyi E. Chukwuma[67], Onwe E. Ogah[115], Onwuamah Chika[36], Oshomah Cyril[25], Ousmane Faye[30], Oyewale Tomori[14], Pascale Ondoa[56], Patrice Combe[116], Patrick Semanda[76], Paul E. Oluniyi[14], Paulo Arnaldo[110], Peter K. Quashie[50], Philippe Dussart[44], Phillip A. Bester[53], Placide K. Mbala[18,19],

Reuben Ayivor-Djanie[85], Richard Njouom[117], Richard O. Phillips[118], Richmond Gorman[118], Robert A. Kingsley[26], Rosina A. A. Carr[85], Saâd El Kabbaj[119], Saba Gargouri[17], Saber Masmoudi[32], Safietou Sankhe[30], Salako B. Lawal[36], Samar Kassim[77], Sameh Trabelsi[120], Samar Metha[33], Sami Kammoun[121], Sanaâ Lemriss[122], Sara H. A. Agwa[77], Sébastien Calvignac-Spencer[59], Stephen F. Schaffner[33], Seydou Doumbia[31], Sheila M. Mandanda[18,19], Sherihane Aryeetey[123], Shymaa S. Ahmed[123], Siham Elhamoumi[33], Soafy Andriamandimby[44], Sobajo Tope[14], Sonia Lekana-Douki[82], Sophie Prosolek[26], Soumeya Ouangraoua[124,125], Steve A. Mundeke[18,19], Steven Rudder[26], Sumir Panji[112], Sureshnee Pillay[1], Susan Engelbrecht[41,72], Susan Nabadda[76], Sylvie Behillil[126], Sylvie L. Budiaki[95], Sylvie van der Werf[126], Tapfumanei Mashe[65], Tarik Aanniz[38], Thabo Mohale[35], Thanh Le-Viet[26], Tobias Schindler[49,97], Ugochukwu J. Anyaneji[1], Ugwu Chinedu[14], Upasana Ramphal[1,69,127], Uwanibe Jessica[14], Uwem George[14], Vagner Fonseca[1,4,128], Vincent Enouf[126], Vivianne Gorova[129,130], Wael H. Roshdy[123], William K. Ampofo[50], Wolfgang Preiser[41,72], Wonderful T. Choga[54,131], Yaw Bediako[50], Yeshnee Naidoo[1], Yvan Butera[108,132,133], Zaydah R. de Laurent[33], Amadou A. Sall[30], Ahmed Rebai[32], Anne von Gottberg[35], Bourema Kouriba[12], Carolyn Williamson[7,69,111], Daniel J. Bridges[105], Ihekweazu Chikwe[99], Jinal Bhiman[35], Madisa Mine[134], Matthew Cotten[40,135], Sikhulile Moyo[54,136], Simani Gaseitsiwe[54,136], Ngonda Saasa[55], Pardis C. Sabeti[33], Pontiano Kaleebu[40], Yenew K. Tebeje[21], Sofonias K. Tessema[21], Christian Happi[14], John Nkengasong[21], Tulio de Oliveira[1,2,69,137]*

[1]KwaZulu-Natal Research Innovation and Sequencing Platform (KRISP), Nelson R Mandela School of Medicine, University of KwaZulu-Natal, Durban, South Africa. [2]Centre for Epidemic Response and Innovation (CERI), School of Data Science and Computational Thinking, Stellenbosch University, Stellenbosch, South Africa. [3]Laboratorio de Flavivirus, Fundacao Oswaldo Cruz, Rio de Janeiro, Brazil. [4]Laboratório de Genética Celular e Molecular, Universidade Federal de Minas Gerais, Belo Horizonte, Minas Gerais, Brazil. [5]Department of Geography and GIS, University of Cincinnati, Cincinnati, OH, USA. [6]Institute of Infectious Diseases and Molecular Medicine, Department of Integrative Biomedical Sciences, Computational Biology Division, University of Cape Town, Cape Town, South Africa. [7]Division of Medical Virology, Wellcome Centre for Infectious Diseases in Africa, Institute of Infectious Disease and Molecular Medicine, University of Cape Town, Cape Town, South Africa. [8]Department of Entomology and Plant Pathology, North Carolina State University, Raleigh, NC, USA. [9]Bioinformatics Research Center, North Carolina State University, Raleigh, NC, USA. [10]Cancer Biology Department, Virology and Immunology Unit, National Cancer Institute, Cairo University, Cairo 11796, Egypt. [11]Centre d'Infectiologie Charles Mérieux-Mali (CICM-Mali), Bamako, Mali. [12]Bacteriology and Virology Department Souro Sanou University Hospital, Bobo-Dioulasso, Burkina Faso. [13]MRCG at LSHTM Genomics Lab, Fajara, Gambia. [14]African Centre of Excellence for Genomics of Infectious Diseases (ACEGID), Redeemer's University, Ede, Osun State, Nigeria. [15]Department of Virology, College of Medicine, University of Ibadan, Ibadan, Nigeria. [16]Department of Medical Microbiology and Parasitology, Faculty of Basic Clinical Sciences, College of Health Sciences, University of Ilorin, Ilorin, Kwara State, Nigeria. [17]CHU Habib Bourguiba, Laboratory of Microbiology, Faculty of Medicine of sFax, University of sFax, sFax, Tunisia. [18]Pathogen Sequencing Lab, Institut National de Recherche Biomédicale (INRB), Kinshasa, Democratic Republic of the Congo. [19]Université de Kinshasa (UNIKIN), Kinshasa, Democratic Republic of the Congo. [20]Genomics Research Program, Children's Cancer Hospital, Cairo, Egypt. [21]Institute of Pathogen Genomics, Africa Centres for Disease Control and Prevention (Africa CDC), Addis Ababa, Ethiopia. [22]Laboratory of Transmissible Diseases and Biological Active Substances (LR99ES27), Faculty of Pharmacy of Monastir, Monastir, Tunisia. [23]Laboratory of Microbiology, University Hospital of Monastir, Monastir, Tunisia. [24]Internal Medicine Department, Alex Ekwueme Federal University Teaching Hospital, Abakaliki, Nigeria. [25]Irrua Specialist Teaching Hospital, Irrua, Nigeria. [26]Quadram Institute Bioscience, Norwich, UK. [27]World Health Organization, Africa Region, Brazzaville Congo. [28]Centre de Recherche et de Formation en Infectiologie de Guinée (CERFIG), Université de Conakry, Conakry, Guinea. [29]TransVIHMI, Montpellier University/IRD/INSERM, Montpellier, France. [30]Virology Department, Institut Pasteur de Dakar, Dakar, Senegal. [31]Mali-University Clinical Research Center (UCRC), Bamako, Mali. [32]Laboratory of Molecular and Cellular Screening Processes, Centre of Biotechnology of Sfax, University of Sfax, Sfax, Tunisia. [33]Broad Insitute of Harvard and MIT, Cambridge, MA, USA. [34]KEMRI-Wellcome Trust Research Programme/KEMRI-CGMR-C, Kilifi, Kenya. [35]National Institute for Communicable Diseases (NICD) of the National Health Laboratory Service (NHLS), Johannesburg, South Africa. [36]The Nigerian Institute of Medical Research, Yaba, Lagos, Nigeria [37]Institute of Virology, Charité – Universitätsmedizin, Berlin, Germany. [38]Medical Biotechnology Laboratory, Rabat Medical and Pharmacy School, Mohammed V University, Rabat, Morocco. [39]Department of Immunology, University of Maiduguri Teaching Hospital, P.M.B. 1414, Maiduguri, Nigeria. [40]MRC/UVRI and LSHTM Uganda Research Unit, Entebbe, Uganda. [41]Division of Medical Virology, Faculty of Medicine and Health Sciences, Stellenbosch University, Tygerberg, Cape Town, South Africa. [42]The Biotechnology Center of the University of Yaoundé I, Cameroon and CDC Foundation, Yaounde, Cameroon. [43]Department of Ecology and Evolution, University of Chicago, Chicago, IL, USA. [44]Virology Unit, Institut Pasteur de Madagascar, Antananarivo, Madagascar. [45]University of Nebraska Medical Center (UNMC), Omaha, NE, USA. [46]Antibody Immunity Research Unit, School of Pathology, University of the Witwatersrand, Johannesburg, South Africa. [47]CHU de Bouaké, Laboratoire/Unité de Diagnostic des Virus des Fièvres Hémorragiques et Virus Émergents, Bouaké, Côte d'Ivoire. [48]School of Public Health, Pwani University, Kilifi, Kenya. [49]Swiss Tropical and Public Health Institute, Basel, Switzerland. [50]West African Centre for Cell Biology of Infectious Pathogens (WACCBIP), Department of Biochemistry, Cell and Molecular Biology, University of Ghana, Accra, Ghana. [51]School of Life Sciences and Zeeman Institute for Systems Biology and Infectious Disease Epidemiology Research (SBIDER), University of Warwick, Coventry, UK. [52]Uganda Virus Research Institute, Entebbe, Uganda. [53]Division of Virology, National Health Laboratory Service and University of the Free State, Bloemfontein, South Africa. [54]Botswana Harvard AIDS Institute Partnership and Botswana Harvard HIV Reference Laboratory, Gaborone, Botswana. [55]University of Zambia, School of Veterinary Medicine, Department of Disease Control, Lusaka, Zambia. [56]African Society for Laboratory Medicine, Addis Ababa, Ethiopia. [57]Functional Genomic Platform/National Centre for Scientific and Technical Research (CNRST), Rabat, Morocco. [58]Rwanda National Reference Laboratory, Kigali, Rwanda. [59]Robert Koch-Institute, Berlin, Germany. [60]G5 Evolutionary Genomics of RNA Viruses, Institut Pasteur, Paris, France. [61]Research Unit of Autoimmune Diseases UR17DN02, Military Hospital of Tunis, University of Tunis El Manar, Tunis, Tunisia. [62]Department of Public Health, Ministry of Health, Ilorin, Kwara State, Nigeria. [63]Laboratory of Clinical Virology, Institut Pasteur de Tunis, Tunis, Tunisia. [64]Faculty of Pharmacy of Monastir, Monastir, Tunisia. [65]National Microbiology Reference Laboratory, Harare, Zimbabwe. [66]National Influenza Centre, Viral Respiratory Laboratory, Algiers, Algeria. [67]Medical Microbiology and Parasitology Department, College of Medicine, University of Ibadan, Ibadan, Nigeria. [68]Lincoln International Institute for Rural Health, University of Lincoln, Lincoln, UK. [69]Centre for the AIDS Programme of Research in South Africa (CAPRISA), Durban, South Africa. [70]Africa Health Research Institute, KwaZulu-Natal, Durban, South Africa. [71]Department of Biochemistry and Biotechnology, Pwani University, Kilifi, Kenya. [72]National Health Laboratory Service (NHLS), Tygerberg, Cape Town, South Africa. [73]Institution and Department, Ministry Of Health, COVID-19 Testing Laboratory, Mbabane, Kingdom of Eswatini. [74]Laboratory of Health Sciences and Technologies, High Institute of Health Sciences, Hassan 1st University, Settat, Morocco. [75]Department of Emerging Infectious Diseases, Institute of Tropical

Medicine, Nagasaki University, Nagasaki, Japan. [76]Central Public Health Laboratories (CPHL), Kampala, Uganda. [77]Faculty of Medicine Ain Shams Research institute (MASRI), Ain Shams University, Cairo, Egypt. [78]Charles Nicolle Hospital, Laboratory of Microbiology, National Influenza Center, 1006 Tunis, Tunisia. [79]University of Tunis El Manar, Faculty of Medicine of Tunis, LR99ES09, 1007 Tunis, Tunisia. [80]Department of Biochemistry and Molecular Biology, The Institute for Medical Research Israel-Canada, Hadassah Medical School, The Hebrew University of Jerusalem, Jerusalem, Israel. [81]Namibia Institute of Pathology, Windhoek, Namibia. [82]Centre Interdisciplinaires de Recherches Medicales de Franceville (CIRMF), Franceville, Gabon. [83]Department of Biostatistics and Data Science, School of Public Health and Tropical Medicine, Tulane University, New Orleans, LA, USA. [84]Urban Health Collaborative, Dornsife School of Public Health, Drexel University, Philadelphia, PA, USA. [85]UHAS COVID-19 Testing and Research Centre, University of Health and Allied Sciences, Ho, Ghana. [86]Rollins School of Public Health, Emory University, Atlanta, GA, USA. [87]Anoual Laboratory, Casablanca, Morocco. [88]Botswana Institute for Technology Research and Innovation, Gaborone, Botswana. [89]New York University Grossman School of Medicine, New York City, NY, USA. [90]Centre de Recherches Medicales de Lambarene (CERMEL), Lambarene, Gabon. [91]Virology/Molecular Biology Department, Central Health Laboratory, Ministry of Health and Wellness, Mauritius. [92]Center of Scientific Excellence for Influenza Viruses, National Research Centre (NRC), Cairo Egypt. [93]Ministry of Health and Wellness, Gaborone, Botswana. [94]Next Generation Sequencing Unit and Division of Virology, Faculty of Health Sciences, University of the Free State, Bloemfontein 9300, South Africa. [95]National Reference Laboratory Lesotho, Maseru, Lesotho. [96]Centre for Biotechnology Research and Development, Kenya Medical Research Institute, Nairobi, Kenya. [97]Laboratorio de Investigaciones de Baney, Baney, Equatorial Guinea. [98]Ifakara Health Institute, Dar-es-Salaam, Tanzania. [99]Nigeria Centre for Disease Control, Abuja, Nigeria. [100]Department of Medical Diagnostics, Kumasi Centre for Collaborative Research in Tropical Medicine, Kwame Nkrumah University of Science and Technology, Kumasi, Ghana. [101]Department of Medical Laboratory Science, Niger Delta University, Bayelsa State, Nigeria. [102]Systems and Biomedical Engineering Department, Faculty of Engineering, Cairo University, Cairo 12613, Egypt. [103]King Faisal Specialist Hospital and Research Center, Riyadh, Kingdom of Saudi Arabia. [104]Biological Prevention Department, Main Chemical Laboratories, Egypt Army, Cairo, Egypt. [105]PATH, Lusaka, Zambia. [106]Department of Biotechnology, High Institute of Biotechnology of Sidi Thabet, University of Manouba, BP-66, 2020 Ariana-Tunis, Tunisia. [107]Genomic Center for Human Pathologies (GENOPATH), Faculty of Medicine and Pharmacy, Mohammed V University, Rabat, Morocco. [108]Rwanda National Joint Task Force COVID-19, Rwanda Biomedical Centre, Ministry of Health, Kigali, Rwanda. [109]School of Health Sciences, College of Medicine and Health Sciences, University of Rwanda, Kigali, Rwanda. [110]Instituto Nacional de Saude (INS), Maputo, Mozambique. [111]National Health Laboratory Service (NHLS), Cape Town, South Africa. [112]Computational Biology Division, Department of Integrative Biomedical Sciences, IDM, CIDRI Africa Wellcome Trust Centre, University of Cape Town, Cape Town, South Africa. [113]Virology Laboratory, Alex Ekwueme Federal University Teaching Hospital, Abakaliki, Nigeria. [114]Department of Epidemiology and Community Health, Faculty of Clinical Sciences, College of Health Sciences, University of Ilorin, Ilorin, Kwara State, Nigeria. [115]Alex Ekwueme Federal University Teaching Hospital, Abakaliki, Nigeria. [116]Mayotte Hospital Center, Mayotte, France. [117]Virology Service, Centre Pasteur of Cameroun, Yaounde, Cameroon. [118]Kumasi Centre for Collaborative Research in Tropical Medicine, Kwame Nkrumah University of Science and Technology, Kumasi, Ghana. [119]Laboratoire de Recherche et d'Analyses Médicales de la Gendarmerie Royale, Rabat, Morocco. [120]Clinical and Experimental Pharmacology Lab, LR16SP02, National Center of Pharmacovigilance, University of Tunis El Manar, Tunis, Tunisia. [121]CHU Hedi Chaker Sfax, Service de Pneumologie, Tunis, Tunisia. [122]Laboratoire de Recherche et d'Analyses Médicales de la Gendarmerie Royale, Rabat, Morocco. [123]Central Public Health Laboratories (CPHL), Cairo, Egypt. [124]Centre MURAZ, Ouagadougou, Burkina Faso. [125]National Institute of Public Health of Burkina Faso (INSP/BF), Ouagadougou, Burkina Faso. [126]National Reference Center for Respiratory Viruses, Molecular Genetics of RNA Viruses, UMR 3569 CNRS, University of Paris, Institut Pasteur, Paris, France. [127]Sub-Saharan African Network For TB/HIV Research Excellence (SANTHE), Durban, South Africa. [128]Coordenação Geral de Laboratórios de Saúde Pública/Secretaria de Vigilância em Saúde, Ministério da Saúde, Brasília, Distrito Federal, Brazil. [129]World Health Organization, WHO Lesotho, Maseru, Lesotho. [130]Med24 Medical Centre, Ruwa, Zimbabwe. [131]Division of Human Genetics, Department of Pathology, University of Cape Town, Cape Town, South Africa. [132]Center for Human Genetics, College of Medicine and Health Sciences, University of Rwanda, Kigali, Rwanda. [133]Laboratory of Human Genetics, GIGA Research Institute, Liège, Belgium. [134]National Health Laboratory, Gaborone, Botswana. [135]MRC-University of Glasgow Centre for Virus Research, Glasgow, UK. [136]Harvard T.H. Chan School of Public Health, Boston, MA, USA. [137]Department of Global Health, University of Washington, Seattle, WA, USA.

†These authors contributed equally to this work. *Corresponding author. Email: tulio@sun.ac.za

The progression of the SARS-CoV-2 pandemic in Africa has so far been heterogeneous and the full impact is not yet well understood. Here, we describe the genomic epidemiology using a dataset of 8746 genomes from 33 African countries and two overseas territories. We show that the epidemics in most countries were initiated by importations predominantly from Europe, which diminished following the early introduction of international travel restrictions. As the pandemic progressed, ongoing transmission in many countries and increasing mobility led to the emergence and spread within the continent of many variants of concern and interest, such as B.1.351, B.1.525, A.23.1 and C.1.1. Although distorted by low sampling numbers and blind spots, the findings highlight that Africa must not be left behind in the global pandemic response, otherwise it could become a source for new variants.

Severe acute respiratory syndrome-related coronavirus 2 (SARS-CoV-2) emerged in late 2019 in Wuhan, China (*1*, *2*). Since then, the virus has spread to all corners of the world, causing almost 150 million cases of coronavirus disease 2019 (COVID-19) and over three million deaths by the end of April 2021. Throughout the pandemic, it has been noted that Africa accounts for a relatively low proportion of reported cases and deaths – by the end of April 2021, there had been ~4.5 million cases and ~120000 deaths on the continent, corresponding to less than 4% of the global burden. However, emerging data from seroprevalence surveys and autopsy studies in some African countries suggests that the true number of infections and deaths may be several fold higher than reported (*3*, *4*). In addition, a recent analysis has shown that the second wave of the pandemic was more severe than the first wave in many African countries (*5*).

The first cases of COVID-19 on the African continent were reported in Nigeria, Egypt and South Africa between mid-February and early March 2020, and most countries had reported cases by the end of March 2020 (*6*–*8*). These early cases were concentrated amongst airline travellers returning from regions of the world with high levels of community

transmission. Many African countries introduced early public health and social measures (PHSM), including international travel controls, quarantine for returning travellers, and internal lockdown measures to limit the spread of the virus and give health services time to prepare (*5*, *9*). The initial phase of the epidemic was then heterogeneous with relatively high case numbers reported in North Africa and Southern Africa, and fewer cases reported in other regions.

From the onset of the pandemic, genomic surveillance has been at the forefront of the COVID-19 response in Africa (*10*). Rapid implementation of SARS-CoV-2 sequencing by various laboratories in Africa enabled genomic data to be generated and shared from the early imported cases. In Nigeria, the first genome sequence was released just three days after the announcement of the first case (*6*). Similarly, in Uganda, a sequencing program was set up rapidly to facilitate virus tracing, and the collection of samples for sequencing began immediately upon confirmation of the first case (*11*). In South Africa, the network for genomic surveillance in South Africa (NGS-SA) was established in March 2020 and within weeks genomic analysis was helping to characterize outbreaks and community transmission (*12*).

Genomic surveillance has also been critical for monitoring ongoing SARS-CoV-2 evolution and detection of new SARS-CoV-2 variants in Africa. Intensified sampling by NGS-SA in the Eastern Cape Province of South Africa in November 2020, in response to a rapid resurgence of cases, led to the detection of B.1.351 (501Y.V2) (*13*). This variant was subsequently designated a variant of concern (VOC) by the World Health Organization (WHO), due to evidence of increased transmissibility (*14*) and resistance to neutralizing antibodies elicited by natural infection and vaccines (*15*–*17*).

Here, we perform phylogenetic and phylogeographic analysis of SARS-CoV-2 genomic data from 33 African countries and two overseas territories to help characterize the dynamics of the pandemic in Africa. We show that the early introductions were predominantly from Europe, but that as the pandemic progressed there was increasing spread between African countries. We also describe the emergence and spread of a number of key SARS-CoV-2 variants in Africa, and highlight how the spread of B.1.351 (501Y.V2) and other variants contributed to the more severe second wave of the pandemic in many countries.

SARS-CoV-2 genomic data

By 5 May 2021, 14504 SARS-CoV-2 genomes had been submitted to the GISAID database (*18*) from 38 African countries and two overseas territories (Mayotte and Réunion) (Fig. 1A). Overall, this corresponds to approximately one sequence per ~300 reported cases. Almost half of the sequences were from South Africa (n=5362), consistent with it being responsible for almost half of the reported cases in Africa. Overall, the number of sequences correlates closely with the number of reported cases per country (Fig. 1B). The countries/territories with the highest coverage of sequencing (defined as genomes per reported case) are Kenya (n=856, one sequence per ~203 cases), Mayotte (n=721, one sequence per ~21 cases), and Nigeria (n=660, one sequence per ~250 cases). Although genomic surveillance started early in many countries, few have evidence of consistent sampling across the whole year. Half of all African genomes were deposited in the first ten weeks of 2021, suggesting intensified surveillance in the second wave following the detection of B.1.351/501Y.V2 and other variants (Fig. 1, C and D).

Genetic diversity and lineage dynamics in Africa

Of the 10326 genomes retrieved from GISAID by the end of March 2021, 8,746 genomes passed quality control (QC) and met the minimum metadata requirements. These genomes from Africa were compared in a phylogenetic framework with 11891 representative genomes from around the world. Ancestral location state reconstruction of the dated phylogeny (hereafter referred to as discrete phylogeographic reconstruction) allowed us to infer the number of viral imports and exports between Africa and the rest of the world, and between individual African countries. African genomes in this study spanned the whole global genetic diversity of SARS-CoV-2, a pattern that largely reflects multiple introductions over time from the rest of the world (Fig. 2A).

In total, we detected at least 757 (95% CI: 728 - 786) viral introductions into African countries between the start of 2020 and February 2021, over half of which occurred before the end of May 2020. While the early phase of the pandemic was dominated by importations from outside Africa, predominantly from Europe, there was then a shift in the dynamics, with an increasing number of importations from other African countries as the pandemic progressed (Fig. 2, B and C). A rarefaction analysis in which we systematically subsampled genomes shows that vastly more introductions would have likely been identified with increased sampling in Africa or globally, suggesting that the introductions we identified are really just the "ears of the hippo," or tip of the iceberg (fig. S1).

South Africa, Kenya and Nigeria appear as major sources of importations into other African countries (Fig. 2D), although this is likely to be influenced by these three countries having the greatest number of deposited sequences. Particularly striking is the southern African region, where South Africa is the source for a large proportion (~80%) of the importations to other countries in the region. The North African region demonstrates a different pattern to the rest of the continent, with more viral introductions from Europe and Asia (particularly the Middle East) than from other African countries (fig. S2).

Africa has also contributed to the international spread of the virus with at least 324 (95% CI: 728 - 786) exportation events from Africa to the rest of the world detected in this dataset. Consistent with the source of importations, most exports were to Europe (41%), Asia (26%) and North America (14%). As with the number of importations exports were relatively evenly distributed over the one year period (fig. S3). However, an increase in the number of exportation events occurred between December 2020 and March 2021, which coincided with the second wave of infections in Africa and with some relaxations of travel restrictions around the world.

The early phase of the pandemic was characterized by the predominance of lineage B.1. This was introduced multiple times to African countries and has been detected in all but one of the countries included in this analysis. After its emergence in South Africa, B.1.351 became the most frequently detected SARS-CoV-2 lineage found in Africa (n=1,769, ~20%) (Fig. 1C). It was first sampled on 8 October 2020 in South Africa (*13*) and has since spread to 20 other African countries.

As air travel came to an almost complete halt in March/April 2020, the number(s) of detectable viral imports into Africa decreased and the pandemic entered a phase that was characterized in sub-Saharan Africa by sustained low levels of within-country movements and occasional international viral movements between neighboring countries, presumably via road and rail links between these. Though some border posts between countries were closed during the initial lockdown period (table S1), others remained open to allow trade to continue. Regional trade in southern Africa was only slightly impacted by lockdown restrictions and quickly rebounded to pre-pandemic levels (fig. S4) following the relaxation of restrictions between June 2020 and December 2020.

Although lineage A viruses were imported into several African countries, they only account for 1.3% of genomes sampled in Africa. Despite lineage A viruses initially causing many localized clustered outbreaks, each the result of independent introductions to several countries (e.g., Burkina Faso, Cote d'Ivoire and Nigeria), they were later largely replaced by lineage B viruses as the pandemic evolved. This is possibly due to the increased transmissibility of B lineage viruses by virtue of the D614G mutation in spike (*19*, *20*). However, there is evidence of an increasing prevalence of lineage A viruses in some African countries (*11*). In particular, A.23.1 emerged in East Africa and appears to be increasing rapidly in prevalence in Uganda and Rwanda (*11*). Furthermore, a highly divergent variant from lineage A was recently identified in Angola from individuals arriving from Tanzania (*21*).

Emergence and spread of new SARS-CoV-2 variants

In order to determine how some of the key SARS-CoV-2 variants are spreading within Africa, we performed phylogeographic analyses on the VOC B.1.351, the variant of interest (VOI) B.1.525, and on two additional variants that emerged and that we designated as VOIs for this analysis (A.23.1 and C.1.1). These African VOCs and VOIs have multiple mutations on Spike glycoprotein and molecular clock analysis of these four datasets provided strong evidence that these four lineages are evolving in a clocklike manner (Fig. 3, A and B).

B.1.351 was first sampled in South Africa in October 2020, but phylogeographic analysis suggests that it emerged earlier, around August 2020. It is defined by ten mutations in the spike protein, including K417N, E484K and N501Y in the receptor-binding domain (Fig. 3B). Following its emergence in the Eastern Cape, it spread extensively within South Africa (Fig. 4A). By November 2020, the variant had spread into neighboring Botswana and Mozambique and by December 2020 it had reached Zambia and Mayotte. Within the first three months of 2021, further exports from South Africa into Botswana, Zimbabwe, Mozambique and Zambia occurred. By March 2021, B.1.351 had become the dominant lineage within most Southern African countries as well as the overseas territories of Mayotte and Réunion (fig. S5). Our phylogeographic reconstruction also demonstrates movement of B.1.351 into East and Central Africa directly from southern Africa. Our discrete phylogeographic analysis of a wider sample of B.1.351 isolates demonstrate the spread of the lineage into West Africa. This patient from West Africa had a known travel history to Europe so it possible the patient acquired the infection while in Europe or in transit and not from other African sources (fig. S6).

B.1.525 is a VOI defined by six substitutions in the spike protein (Q52R, A67V, E484K, D614G, O677H and F888L), and two deletions in the N-terminal domain (HV69-70△ and Y144△). This was first sampled in the United Kingdom in mid-December 2020, but our phylogeographic reconstruction suggests that the variant originated in Nigeria in November 2020 [95% highest posterior density (HPD) 2020-11-01 to 2020-12-03] (Fig. 4B). Since then it has spread throughout much of Nigeria and neighboring Ghana. Given sparse sampling from other neighboring countries within West and Central Africa (Fig. 1, A and C), the extent of the spread of this VOI in the region is not clear. Beyond Africa, this VOI has spread to Europe and the US (fig. S6).

We designated A.23.1 and C.1.1 as VOIs for the purposes of this analysis, as they present good examples of the continued evolution of the virus within Africa (*11*, *13*). Lineage A.23, characterized by three spike mutations (F157L, V367F and Q613H), was first detected in a Ugandan prison in Amuru in July 2020 (95% HPD: 2020-07-15 to 2020-08-02). From there, the lineage was transmitted to Kitgum prison, possibly facilitated by the transfer of prisoners. Subsequently, the A.23 lineage spilled into the general population and spread to

Kampala, adding other spike mutations (R102I, L141F, E484K, P681R) along with additional mutations in nsp3, nsp6, ORF8 and ORF9, prompting a new lineage classification, A.23.1 (Fig. 3, A and B). Since the emergence of A.23.1 in September 2020 (95% HPD: 2020-09-02 to 2020-09-28), it has spread regionally into neighboring Rwanda and Kenya and has now also reached South Africa and Botswana in the south and Ghana in the west (Fig. 4C). However, our phylogeographic reconstruction of A.23.1 suggests that the introduction into Ghana may have occurred via Europe (fig. S6), whereas the introductions into southern Africa likely occurred directly from East Africa. This is consistent with epidemiological data suggesting that the case detected in South Africa was a contact of an individual who had recently travelled to Kenya.

Lineage C.1 emerged in South Africa in March 2020 (95% HPD: 2020-03-13 to 2020-04-17) during a cluster outbreak prior to the first wave of the epidemic (*13*). C.1.1 is defined by the spike mutations S477N, A688S, M1237I and also contains the Q52R and A67V mutations similar to B.1.525 (Fig. 3B). A continuous trait phylogeographic reconstruction of the movement dynamics of these lineages suggests that C.1 emerged in the city of Johannesburg and spread within South Africa during the first wave (Fig. 4D). Independent exports of C.1 from South Africa led to regional spread to Zambia (June-July, 2020) and Mozambique (July-August 2020), and the evolution to C.1.1 seems to have occurred in Mozambique around mid-September 2020 (95% HPD: 2020-09-07 to 2020-10-05). In depth analysis of SARS-CoV-2 genotypes from Mozambique suggest that the C.1.1. lineage was the most prevalent in the country until the introduction of B.1.351, which has dominated the epidemic since (fig. S5).

The VOC B.1.1.7, which was first sampled in Kent, England in September 2020 (*22*), has also increased in prevalence in several African countries (fig. S5) To date, this VOC has been detected in eleven African countries, as well as the Indian Ocean islands of Mauritius and Mayotte (fig. S7). The time-resolved phylogeny suggests that this lineage was introduced into Africa on at least 16 occasions between November 2020 and February 2021 with evidence of local transmission in Nigeria and Ghana.

Conclusions

Our phylogeographic reconstruction of past viral dissemination patterns suggests a strong epidemiological linkage between Europe and Africa, with 64% of detectable viral imports into Africa originating in Europe and 41% of detectable viral exports from Africa landing in Europe (Fig. 1C). This phylogeographic analysis also suggests a changing pattern of viral diffusion into and within Africa over the course of 2020. In almost all instances the earliest introductions of SARS-CoV-2 into individual African countries were from countries outside Africa.

High rates of COVID-19 testing and consistent genomic surveillance in the south of the continent have led to the early identification of VOCs such as B.1.351 and VOIs such as C.1.1 (*13*). Since the discovery of these southern African variants, several other SARS-CoV-2 VOIs have emerged in different parts of the world, including elsewhere on the African continent, such as B.1.525 in West Africa and A.23.1 in East Africa. There is strong evidence that both of these VOIs are rising in frequency in the regions where they have been detected, which suggests that they may possess higher fitness than other variants in these regions. Although more focused research on the biological properties of these VOIs is needed to confirm whether they should be considered VOCs, it would be prudent to assume the worst and focus on limiting their spread. It will be important to investigate how these different variants compete against one another if they occupy the same region.

Our focused phylogenetic analysis of the B.1.351 lineage revealed that in the final months of 2020 this variant spread from South Africa into neighboring countries, reaching as far north as the DRC by February 2021. This spread may have been facilitated through rail and road networks that form major transport arteries linking South Africa's ocean ports to commercial and industrial centres in Botswana, Zimbabwe, Zambia and the southern parts of the DRC. The rapid, apparently unimpeded spread of B.1.351 into these countries suggests that current land-border controls that are intended to curb the international spread of the virus are ineffective. Perhaps targeted testing of cross-border travellers, genotyping of positive cases and the focused tracking of frequent cross-border travellers such as long distance truckers, would more effectively contain the spread of future VOCs and VOIs that emerge within this region.

The dominance of VOIs and VOCs in Africa has important implications for vaccine rollouts on the continent. For one, slow rollout of vaccines in most African countries creates an environment in which the virus can replicate and evolve: this will almost certainly produce additional VOCs, any of which could derail the global fight against COVID-19. On the other hand, with the already widespread presence of known variants, difficult decisions balancing reduced efficacy and availability of vaccines have to be made. This also highlights how crucial it is that trials are done. From a public health perspective, genomic surveillance is only one item in the toolkit of pandemic preparedness. It is important that such work is closely followed by genotype to phenotype research to determine the actual significance of continued evolution of SARS-CoV-2 and other emerging pathogens.

The rollout of vaccines across Africa has been painfully slow (figs. S8 and S9). There have, however, been notable successes that suggest the situation is not hopeless. The small

island nation of the Seychelles had vaccinated 70% of its population by May 2021. Morocco has kept pace with many developed nations and by mid-March had vaccinated ~16% of its population. Rwanda, one of Africa's most resource constrained countries, had, within three weeks of obtaining its first vaccine doses in early March, managed to provide first doses to ~2.5% of its population. For all other African countries, at the time of writing, vaccine coverage (first dose) was <1.0% of the general population.

The effectiveness of molecular surveillance as a tool for monitoring pandemics is largely dependent on continuous and consistent sampling through time, rapid virus genome sequencing and rapid reporting. When this is achieved, molecular surveillance can ensure the early detection of changing pandemic characteristics. Further, when such changes are discovered, molecular surveillance data can also guide public health responses. In this regard, the molecular surveillance data that are being gathered by most African countries are less useful than they could be. For example, the time-lag between when virus samples are taken and when sequences for these samples are deposited in sequence repositories is so great in some cases that the primary utility of genomic surveillance data is lost (fig. S10). This lag is driven by several factors depending on the laboratory or country in question: (*i*) lack of reagents due to disruptions in global supply chains, (*ii*) lack of equipment and infrastructure within the originating country, (*iii*) scarcity of technical skills in laboratory methods or bioinformatic support, and (*iv*) hesitancy by some health officials to release data. More recent sampling and prompt reporting is crucial to reveal the genetic characteristics of currently circulating viruses in these countries.

The patchiness of African genomic surveillance data is therefore the main weakness of our study. However, there is evidence that the situation is improving, with ~50% of African SARS-CoV-2 genome sequences having been submitted to the GISAID database within the first 10-weeks of 2021. While the precise factors underlying this surge in sequencing effort are unclear, important drivers are almost certainly both increased global interest in genomic surveillance following the discovery of multiple VOCs and VOIs since December 2020. We cannot reject that the observed increase in exports from Africa may be due to intensified sequencing activity following the detection of variants around the world. It is important to note here that phylogeographic reconstruction of viral spread is highly dependent on sampling where there is the caveat that the exact routes of viral movements between countries cannot be inferred if there is no sampling in connecting countries. Furthermore, our efforts to reconstruct the movement dynamics of SARS-CoV-2 across the continent are almost certainly biased by uneven sampling between different African countries. It is not a coincidence that we identified South Africa, Kenya and Nigeria, which have sampled and sequenced the most SARS-CoV-2 genomes, as major sources of viral transmissions between sub-Saharan African countries. However, these countries had also the highest number of infections, which may decrease the sampling biases (Fig. 1A).

The reliability of genomic surveillance as a tool to prevent the emergence and spread of dangerous variants is dependent on the intensity with which it is embraced by national public health programs. As with most other parts of the world, the success of genomic surveillance in Africa requires more samples being tested for COVID-19, higher proportions of positive samples being sequenced within days of sampling, and persistent analyses of these sequences for concerning signals such as (*i*) the presence of novel non-synonymous mutations at genomic sites associated with pathogenicity and immunogenicity, (*ii*) evidence of positive selection at codon sites where non-synonymous mutations are observed, and (*iii*) evidence of lineage expansions. In spite of limited sampling, Africa has identified many of the VOCs and VOIs that are being transmitted across the world. Detailed characterization of the variants and their impact on vaccine induced immunity is of extreme importance. If the pandemic is not controlled in Africa, we may see the production of vaccine escape variants that may profoundly affect the population in Africa and across the world.

REFERENCES AND NOTES

1. C. Wang, P. W. Horby, F. G. Hayden, G. F. Gao, A novel coronavirus outbreak of global health concern. *Lancet* **395**, 470–473 (2020). doi:10.1016/S0140-6736(20)30185-9 Medline
2. Q. Li, X. Guan, P. Wu, X. Wang, L. Zhou, Y. Tong, R. Ren, K. S. M. Leung, E. H. Y. Lau, J. Y. Wong, X. Xing, N. Xiang, Y. Wu, C. Li, Q. Chen, D. Li, T. Liu, J. Zhao, M. Liu, W. Tu, C. Chen, L. Jin, R. Yang, Q. Wang, S. Zhou, R. Wang, H. Liu, Y. Luo, Y. Liu, G. Shao, H. Li, Z. Tao, Y. Yang, Z. Deng, B. Liu, Z. Ma, Y. Zhang, G. Shi, T. T. Y. Lam, J. T. Wu, G. F. Gao, B. J. Cowling, B. Yang, G. M. Leung, Z. Feng, Early transmission dynamics in Wuhan, China, of novel coronavirus-infected pneumonia. *N. Engl. J. Med.* **382**, 1199–1207 (2020). doi:10.1056/NEJMoa2001316 Medline
3. S. Uyoga, I. M. O. Adetifa, H. K. Karanja, J. Nyagwange, J. Tuju, P. Wanjiku, R. Aman, M. Mwangangi, P. Amoth, K. Kasera, W. Ng'ang'a, C. Rombo, C. Yegon, K. Kithi, E. Odhiambo, T. Rotich, I. Orgut, S. Kihara, M. Otiende, C. Bottomley, Z. N. Mupe, E. W. Kagucia, K. E. Gallagher, A. Etyang, S. Voller, J. N. Gitonga, D. Mugo, C. N. Agoti, E. Otieno, L. Ndwiga, T. Lambe, D. Wright, E. Barasa, B. Tsofa, P. Bejon, L. I. Ochola-Oyier, A. Agweyu, J. A. G. Scott, G. M. Warimwe, Seroprevalence of anti-SARS-CoV-2 IgG antibodies in Kenyan blood donors. *Science* **371**, 79–82 (2021). doi:10.1126/science.abe1916 Medline
4. L. Mwananyanda, C. J. Gill, W. MacLeod, G. Kwenda, R. Pieciak, Z. Mupila, R. Lapidot, F. Mupeta, L. Forman, L. Ziko, L. Etter, D. Thea, Covid-19 deaths in Africa: Prospective systematic postmortem surveillance study. *BMJ* **372**, n334 (2021). Medline
5. S. J. Salyer, J. Maeda, S. Sembuche, Y. Kebede, A. Tshangela, M. Moussif, C. Ihekweazu, N. Mayet, E. Abate, A. O. Ouma, J. Nkengasong, The first and second waves of the COVID-19 pandemic in Africa: A cross-sectional study. *Lancet* **397**, 1265–1275 (2021). doi:10.1016/S0140-6736(21)00632-2 Medline
6. P. Oluniyi, First African SARS-CoV-2 genome sequence from Nigerian COVID-19 case. Virological (2020); https://virological.org/t/first-african-sars-cov-2-genome-sequence-from-nigerian-covid-19-case/421.
7. M. A. Medhat, M. El Kassas, COVID-19 in Egypt: Uncovered figures or a different situation? *J. Glob. Health* **10**, 010368 (2020). doi:10.7189/jogh.10.010368 Medline
8. M. Allam, A. Ismail, Z. T. H. Khumalo, S. Kwenda, P. van Heusden, R. Cloete, C. K. Wibmer, P. S. Mtshali, F. Mnyameni, T. Mohale, K. Subramoney, S. Walaza, W.

Ngubane, N. Govender, N. V. Motaze, J. N. Bhiman, SA-COVID-19 Response Team, Genome sequencing of a severe acute respiratory syndrome coronavirus 2 isolate obtained from a South African Patient with coronavirus disease 2019. *Microbiol. Resour. Announc.* **9**, e00572–e20 (2020). doi:10.1128/MRA.00572-20 Medline
9. N. Haider, A. Y. Osman, A. Gadzekpo, G. O. Akipede, D. Asogun, R. Ansumana, R. J. Lessells, P. Khan, M. M. A. Hamid, D. Yeboah-Manu, L. Mboera, E. H. Shayo, B. T. Mmbaga, M. Urassa, D. Musoke, N. Kapata, R. A. Ferrand, P.-C. Kapata, F. Stigler, T. Czypionka, A. Zumla, R. Kock, D. McCoy, Lockdown measures in response to COVID-19 in nine sub-Saharan African countries. *BMJ Glob. Health* **5**, e003319 (2020). doi:10.1136/bmjgh-2020-003319 Medline
10. S. C. Inzaule, S. K. Tessema, Y. Kebede, A. E. Ogwell Ouma, J. N. Nkengasong, Genomic-informed pathogen surveillance in Africa: Opportunities and challenges. *Lancet Infect. Dis.* **21**, e281–e289 (2021). doi:10.1016/S1473-3099(20)30939-7 Medline
11. D. L. Bugembe, M. V. T. Phan, I. Ssewanyana, P. Semanda, H. Nansumba, B. Dhaala, S. Nabadda, Á. N. O'Toole, A. Rambaut, P. Kaleebu, M. Cotten, A SARS-CoV-2 lineage A variant (A.23.1) with altered spike has emerged and is dominating the current Uganda epidemic. medRxiv 2021.02.08.21251393 (2021); https://doi.org/10.1101/2021.02.08.21251393.
12. J. Giandhari, S. Pillay, E. Wilkinson, H. Tegally, I. Sinayskiy, M. Schuld, J. Lourenço, B. Chimukangara, R. Lessells, Y. Moosa, I. Gazy, M. Fish, L. Singh, K. Sedwell Khanyile, V. Fonseca, M. Giovanetti, L. Carlos Junior Alcantara, F. Petruccione, T. de Oliveira, Early transmission of SARS-CoV-2 in South Africa: An epidemiological and phylogenetic report. *Int. J. Infect. Dis.* **103**, 234–241 (2021). doi:10.1016/j.ijid.2020.11.128 Medline
13. H. Tegally, E. Wilkinson, R. J. Lessells, J. Giandhari, S. Pillay, N. Msomi, K. Mlisana, J. N. Bhiman, A. von Gottberg, S. Walaza, V. Fonseca, M. Allam, A. Ismail, A. J. Glass, S. Engelbrecht, G. Van Zyl, W. Preiser, C. Williamson, F. Petruccione, A. Sigal, I. Gazy, D. Hardie, N. Hsiao, D. Martin, D. York, D. Goedhals, E. J. San, M. Giovanetti, J. Lourenço, L. C. J. Alcantara, T. de Oliveira, Sixteen novel lineages of SARS-CoV-2 in South Africa. *Nat. Med.* **27**, 440–446 (2021). doi:10.1038/s41591-021-01255-3 Medline
14. C. A. Pearson, T. W. Russell, N. Davies, A. J. Kucharski, Estimates of severity and transmissibility of novel SARS-CoV-2 variant 501Y.V2 in South Africa. CMMID Repository (2021); https://cmmid.github.io/topics/covid19/sa-novel-variant.html.
15. S. Cele, I. Gazy, L. Jackson, S.-H. Hwa, H. Tegally, G. Lustig, J. Giandhari, S. Pillay, E. Wilkinson, Y. Naidoo, F. Karim, Y. Ganga, K. Khan, M. Bernstein, A. B. Balazs, B. I. Gosnell, W. Hanekom, M.-Y. S. Moosa, R. J. Lessells, T. Oliveira, A. Sigal, Escape of SARS-CoV-2 501Y.V2 variants from neutralization by convalescent plasma. medRxiv 2021.01.26.21250224 (2021); https://doi.org/10.1101/2021.01.26.21250224.
16. S. A. Madhi, V. Baillie, C. L. Cutland, M. Voysey, A. L. Koen, L. Fairlie, S. D. Padayachee, K. Dheda, S. L. Barnabas, Q. E. Bhorat, C. Briner, G. Kwatra, K. Ahmed, P. Aley, S. Bhikha, J. N. Bhiman, A. E. Bhorat, J. du Plessis, A. Esmail, M. Groenewald, E. Horne, S.-H. Hwa, A. Jose, T. Lambe, M. Laubscher, M. Malahleha, M. Masenya, M. Masilela, S. McKenzie, K. Molapo, A. Moultrie, S. Oelofse, F. Patel, S. Pillay, S. Rhead, H. Rodel, L. Rossouw, C. Taoushanis, H. Tegally, A. Thombrayil, S. van Eck, C. K. Wibmer, N. M. Durham, E. J. Kelly, T. L. Villafana, S. Gilbert, A. J. Pollard, T. de Oliveira, P. L. Moore, A. Sigal, A. Izu, NGS-SA Group, Wits-VIDA COVID Group, Efficacy of the ChAdOx1 nCoV-19 Covid-19 vaccine against the B.1.351 variant. *N. Engl. J. Med.* **384**, 1885–1898 (2021). doi:10.1056/NEJMoa2102214 Medline
17. C. K. Wibmer, F. Ayres, T. Hermanus, M. Madzivhandila, P. Kgagudi, B. Oosthuysen, B. E. Lambson, T. de Oliveira, M. Vermeulen, K. van der Berg, T. Rossouw, M. Boswell, V. Ueckermann, S. Meiring, A. von Gottberg, C. Cohen, L. Morris, J. N. Bhiman, P. L. Moore, SARS-CoV-2 501Y.V2 escapes neutralization by South African COVID-19 donor plasma. *Nat. Med.* **27**, 622–625 (2021). doi:10.1038/s41591-021-01285-x Medline
18. Y. Shu, J. McCauley, GISAID: Global initiative on sharing all influenza data - from vision to reality. *Euro Surveill.* **22**, 30494 (2017). doi:10.2807/1560-7917.ES.2017.22.13.30494 Medline
19. E. Volz, V. Hill, J. T. McCrone, A. Price, D. Jorgensen, Á. O'Toole, J. Southgate, R. Johnson, B. Jackson, F. F. Nascimento, S. M. Rey, S. M. Nicholls, R. M. Colquhoun, A. da Silva Filipe, J. Shepherd, D. J. Pascall, R. Shah, N. Jesudason, K. Li, R. Jarrett, N. Pacchiarini, M. Bull, L. Geidelberg, I. Siveroni, I. Goodfellow, N. J. Loman, O. G. Pybus, D. L. Robertson, E. C. Thomson, A. Rambaut, T. R. Connor, COG-UK Consortium, Evaluating the effects of SARS-CoV-2 spike mutation D614G on transmissibility and pathogenicity. *Cell* **184**, 64–75.e11 (2021). doi:10.1016/j.cell.2020.11.020 Medline
20. B. Korber, W. M. Fischer, S. Gnanakaran, H. Yoon, J. Theiler, W. Abfalterer, N. Hengartner, E. E. Giorgi, T. Bhattacharya, B. Foley, K. M. Hastie, M. D. Parker, D. G. Partridge, C. M. Evans, T. M. Freeman, T. I. de Silva, C. McDanal, L. G. Perez, H. Tang, A. Moon-Walker, S. P. Whelan, C. C. LaBranche, E. O. Saphire, D. C. Montefiori, A. Angyal, R. L. Brown, L. Carrilero, L. R. Green, D. C. Groves, K. J. Johnson, A. J. Keeley, B. B. Lindsey, P. J. Parsons, M. Raza, S. Rowland-Jones, N. Smith, R. M. Tucker, D. Wang, M. D. Wyles, Sheffield COVID-19 Genomics Group, Tracking changes in SARS-CoV-2 spike: Evidence that D614G increases infectivity of the COVID-19 virus. *Cell* **182**, 812–827.e19 (2020). doi:10.1016/j.cell.2020.06.043 Medline
21. T. de Oliveira, S. Lutucuta, J. Nkengasong, J. Morais, J. P. Paixão, Z. Neto, P. Afonso, J. Miranda, K. David, L. Inglês, A. P. A. P. Raisa Rivas Carralero, H. R. Freitas, F. Mufinda, S. K. Tessema, H. Tegally, E. J. San, E. Wilkinson, J. Giandhari, S. Pillay, M. Giovanetti, Y. Naidoo, A. Katzourakis, M. Ghafari, L. Singh, D. Tshiabuila, D. Martin, R. J. Lessells, A novel variant of interest of SARS-CoV-2 with multiple spike mutations is identified from travel surveillance in Africa. medRxiv 2021.03.30.21254323 (2021); https://doi.org/10.1101/2021.03.30.21254323.
22. B. Meng, S. A. Kemp, G. Papa, R. Datir, I. A. T. M. Ferreira, S. Marelli, W. T. Harvey, S. Lytras, A. Mohamed, G. Gallo, N. Thakur, D. A. Collier, P. Mlcochova, L. M. Duncan, A. M. Carabelli, J. C. Kenyon, A. M. Lever, A. De Marco, C. Saliba, K. Culap, E. Cameroni, N. J. Matheson, L. Piccoli, D. Corti, L. C. James, D. L. Robertson, D. Bailey, R. K. Gupta, COVID-19 Genomics UK (COG-UK) Consortium, Recurrent emergence of SARS-CoV-2 spike deletion H69/V70 and its role in the Alpha variant B.1.1.7. *Cell Rep.* **35**, 109292 (2021). doi:10.1016/j.celrep.2021.109292 Medline
23. S. E. James, T. Houriiyah, krisp-kwazulu-natal/africa-covid19-genomics: A year of genomic surveillance reveals how the SARS-CoV-2 pandemic unfolded in Africa - Code and scripts. Zenodo (2021); https://doi.org/10.5281/zenodo.5386379.
24. K. Katoh, D. M. Standley, MAFFT multiple sequence alignment software version 7: Improvements in performance and usability. *Mol. Biol. Evol.* **30**, 772–780 (2013). doi:10.1093/molbev/mst010 Medline
25. F. Wu, S. Zhao, B. Yu, Y.-M. Chen, W. Wang, Z.-G. Song, Y. Hu, Z.-W. Tao, J.-H. Tian, Y.-Y. Pei, M.-L. Yuan, Y.-L. Zhang, F.-H. Dai, Y. Liu, Q.-M. Wang, J.-J. Zheng, L. Xu, E. C. Holmes, Y.-Z. Zhang, A new coronavirus associated with human respiratory disease in China. *Nature* **579**, 265–269 (2020). doi:10.1038/s41586-020-2008-3 Medline
26. L.-T. Nguyen, H. A. Schmidt, A. von Haeseler, B. Q. Minh, IQ-TREE: A fast and effective stochastic algorithm for estimating maximum-likelihood phylogenies. *Mol. Biol. Evol.* **32**, 268–274 (2015). doi:10.1093/molbev/msu300 Medline
27. F. Lemoine, J. B. Domelevo Entfellner, E. Wilkinson, D. Correia, M. Dávila Felipe, T. De Oliveira, O. Gascuel, Renewing Felsenstein's phylogenetic bootstrap in the era of big data. *Nature* **556**, 452–456 (2018). doi:10.1038/s41586-018-0043-0 Medline
28. P. Sagulenko, V. Puller, R. A. Neher, TreeTime: Maximum-likelihood phylodynamic analysis. *Virus Evol.* **4**, vex042 (2018). doi:10.1093/ve/vex042 Medline
29. A. Rambaut, E. C. Holmes, Á. O'Toole, V. Hill, J. T. McCrone, C. Ruis, L. du Plessis, O. G. Pybus, A dynamic nomenclature proposal for SARS-CoV-2 lineages to assist genomic epidemiology. *Nat. Microbiol.* **5**, 1403–1407 (2020). doi:10.1038/s41564-020-0770-5 Medline
30. A. J. Drummond, M. A. Suchard, D. Xie, A. Rambaut, Bayesian phylogenetics with BEAUti and the BEAST 1.7. *Mol. Biol. Evol.* **29**, 1969–1973 (2012). doi:10.1093/molbev/mss075 Medline
31. P. Lemey, A. Rambaut, J. J. Welch, M. A. Suchard, Phylogeography takes a relaxed random walk in continuous space and time. *Mol. Biol. Evol.* **27**, 1877–1885 (2010). doi:10.1093/molbev/msq067 Medline
32. A. Rambaut, T. T. Lam, L. Max Carvalho, O. G. Pybus, Exploring the temporal structure of heterochronous sequences using TempEst (formerly Path-O-Gen). *Virus Evol.* **2**, vew007 (2016). doi:10.1093/ve/vew007 Medline
33. S. Dellicour, R. Rose, N. R. Faria, P. Lemey, O. G. Pybus, SERAPHIM: Studying

environmental rasters and phylogenetically informed movements. *Bioinformatics* **32**, 3204–3206 (2016). doi:10.1093/bioinformatics/btw384 Medline

34. E. Wilkinson, D. Rasmussen, O. Ratmann, T. Stadler, S. Engelbrecht, T. de Oliveira, Origin, imports and exports of HIV-1 subtype C in South Africa: A historical perspective. *Infect. Genet. Evol.* **46**, 200–208 (2016). doi:10.1016/j.meegid.2016.07.008 Medline
35. J. Sukumaran, M. T. Holder, DendroPy: A Python library for phylogenetic computing. *Bioinformatics* **26**, 1569–1571 (2010). doi:10.1093/bioinformatics/btq228 Medline
36. D. Sankoff, Minimal mutation trees of sequences. *SIAM J. Appl. Math.* **28**, 35–42 (1975). doi:10.1137/0128004
37. International Trade Statistics Database, UN Comtrade Database; https://comtrade.un.org/.
38. Johns Hopkins University (JHU) Center for Systems Science and Engineering (CSSE), CSSEGISandData/COVID-19, Novel coronavirus (COVID-19) cases, GitHub; https://github.com/CSSEGISandData/COVID-19.

ACKNOWLEDGMENTS

We gratefully acknowledge the authors from the originating laboratories and the submitting laboratories, who generated and shared via GISAID genetic sequence data on which this research is based (table S4). We also wish to acknowledge the contribution of Kruger Maria from the National Genomics Surveillance of South Africa (NGS-SA) platform for their contribution toward the sequencing effort in Cape Town, South Africa. Similarly, we wish to thank Aya M Elsaame, Shimaa M, Elsayed and Reham M. Darwish from the Faculty of Medicine Ain Shams Research institute (MASRI), for their efforts toward sequencing in Egypt. Sidy Bane, Moumine Sanogo, Dramane Diallo, Antieme Combo Georges Togo and Aminatou Coulibaly from the University Clinical Research Centre (UCRC), at the University of Sciences, Techniques and Technologies of Bamako we wish to extend our thanks for the contribution they have made toward sequencing efforts in Mali. Finally, we wish to acknowledge the contribution of Dr Matshidiso Moeti and Dr Abdou Salam Gueye from the World Health Organization for their contribution toward combating SARS-CoV-2 on the African continent. **Funding:** The University of Ghana (WACCBIP) team was funded by a Wellcome/African Academy of Sciences Developing Excellence in Leadership Training and Science (DELTAS) grant (DEL-15-007 and 107755/Z/15/Z: Awandare), National Institute of Health Research (NIHR) (17.63.91) grants using UK aid from the UK Government for a global health research group for Genomic surveillance of malaria in West Africa (Wellcome Sanger Institute, UK) and global research unit for Tackling Infections to Benefit Africa (TIBA partnership, University of Edinburgh), and the World Bank African Centres of Excellent grant (WACCBIP-NCDs: Awandare). Project ADAGE PRFCOV19-GP2 (2020-2022), includes 40 researchers from the Center of Biotechnology of Sfax, the University of Sfax, the University of Monastir, the University Hospital Hédi Chaker of Sfax, the Military Hospital of Tunis, and Dacima Consulting. Ministry of Higher Education and Scientific Research and Ministry of Health of the Republic of Tunisia. The Uganda contributions were funded by the UK Medical Research Council (MRC/UKRI) and the UK Department for International Development (DFID) under the MRC/DFID Concordat agreement (grant agreement number NC_PC_19060) and by the Wellcome, DFID - Wellcome Epidemic Preparedness – Coronavirus (grant agreement number 220977/Z/20/Z) awarded to MC. Work from Quadram Institute Bioscience was funded by The Biotechnology and Biological Sciences Research Council Institute Strategic Programme Microbes in the Food Chain BB/R012504/1 and its constituent projects BBS/E/F/000PR10348, BBS/E/F/000PR10349, BBS/E/F/000PR10351, and BBS/E/F/000PR10352 and by the Quadram Institute Bioscience BBSRC funded Core Capability Grant (project number BB/CCG1860/1). The Africa Pathogen Genomics Initiative (Africa PGI) at the Africa CDC is supported by the Bill and Melinda Gates Foundation (INV018978 and INV018278), Illumina Inc, Center for Disease Control and Prevention (CDC), and Oxford Nanopore Technologies. Sequences generated in Zambia through PATH were funded by the Bill & Melinda Gates Foundation. The findings and conclusions contained within are those of the authors and do not necessarily reflect positions or policies of the Bill & Melinda Gates Foundation. Funding for sequencing in Côte d'Ivoire, Burkina Faso and part of the sequencing in the Democratic Republic of the Congo was granted by the German Federal Ministry of Education and Research (BMBF). Sequencing efforts from Morocco have been supported by Academie Hassan II of Science and Technology, Morocco. Funding for surveillance, sampling and testing in Madagasar: World Health Organization (WHO), the US Centers for Disease Control and Prevention (US CDC: Grant#U5/IP000812-05), the United States Agency for International Development (USAID: Cooperation Agreement 72068719CA00001), the Office of the Assistant Secretary for Preparedness and Response in the U.S. Department of Health and Human Services (DHHS: grant number IDSEP190051-01-0200). Finding for sequencing: Bill & Melinda Gates Foundation (GCE/ID OPP1211841), Chan Zuckerberg Biohub, and the Innovative Genomics Institute at UC Berkeley. Botswana Harvard AIDS Institute was supported by the following funding: H3ABioNet through funding from the National Institutes of Health Common Fund [U41HG006941]. H3ABioNet is an initiative of the Human Health and Heredity in Africa Consortium (H3Africa) program of the African Academy of Science (AAS). HHS/NIH/National Institute of Allergy and Infectious Diseases (NIAID) (5K24AI131928-04, 5K24AI131924-04), Sub-Saharan African Network for TB/HIV Research Excellence (SANTHE), a DELTAS Africa Initiative [grant # DEL-15-006]. The DELTAS Africa Initiative is an independent funding scheme of the African Academy of Sciences (AAS)'s Alliance for Accelerating Excellence in Science in Africa (AESA) and supported by the New Partnership for Africa's Development Planning and Coordinating Agency (NEPADAgency) with funding from the Wellcome Trust [grant #107752/Z/15/Z] and the United Kingdom (UK) government. South African Medical Research Council (SAMRC) and the Department of Technology and Innovation as part of the Network for Genomic Surveillance in South Africa (NGS-SA) and the Stellenbosch University Faculty of Medicine & Health Sciences, Strategic Equipment Fund". Darren P. Martin is funded by the Wellcome Trust (Wellcome Trust grant number 222574/Z/21/Z). Cathrine Scheepers at the NICD is supported by the National Institute of Allergy and Infectious Diseases of the National Institutes of Health under the Award Number U01AI136677. Furthermore, pandemic surveillance in South Africa and Senegal was supported in part through National Institutes of Health USA grant U01 AI151698 for the United World Antiviral Research Network (UWARN). **Support for pandemic surveillance from Prof. Tulio de Oliveira group to other African countries is funded by the Rockefeller Foundation.** Sequencing efforts in the Democratic Republic of the Congo were funded by the Bill & Melinda Gates Foundation under grant INV-018030 awarded to CBP and further supported by funding from the Africa CDC through the ASLM (African Society of Laboratory Medicine) for Accelerating SARS-CoV-2 Genomic Surveillance in Africa. Sequencing efforts in Rwanda were commissioned by the National Institute of Health Research (NIHR) Global Health Research program (16/136/33) using UK aid from the UK government (funding to EM and NR through TIBA partnership) and additional funds from the Government of Rwanda through RBC/National Reference Laboratory in collaboration with the Belgian Development Agency (ENABEL) for additional genomic sequencing at GIGA Research Institute-Liege/Belgium. The sequencing effort in Equatorial Guinea was supported by a public–private partnership, the Bioko Island Malaria Elimination Project, composed of the government of Equatorial Guinea Ministries of Mines and Hydrocarbons, and Health and Social Welfare, Marathon EG Production Limited, Noble Energy, Atlantic Methanol Production Company, and EG LNG. Samples collection and typing in Mali were supported by Fondation Merieux-France and Sequence efforts has been supported by the by the Enable and Enhance Initiative of the German Federal Government's Security Cooperation against Biological Threats in the G5 Sahel Region. The Nigeria work was made possible by support from Flu Lab and a cohort of generous donors through TED's Audacious Project, including the ELMA Foundation, MacKenzie Scott, the Skoll Foundation, and Open Philanthropy. Further Nigeria funding were supported by grants from the National Institute of Allergy and Infectious Diseases (https://www.niaid.nih.gov), NIH-H3Africa (https://h3africa.org) (U01HG007480 and U54HG007480), the World Bank grant (worldbank.org) (ACE IMPACT project) to Christian Happi. **Author contributions:** Conceptualization: EW, HT†, JGS, JO, JOG, KOD, RAD, RAK, RL, SKT, SM±, TdO. Methodology: ANZ, AR, CA, DPM, DR, EW, HT†, JAE, JG†, JGS, KHY, KOD, LdOM, MAB, MC, MG, MMN, MVTP, PA, TdO, VF. Investigation: AE, AI†, ANZ, AR, AS†, AvG, CAK, CW, DC, DN, DPM, DR, DS, EM, EN, EW, FL, GG, HT†, JAE, JES, JG†,

JGS, JJT, JL, JN‡, JQM, KHY, M-MD, MAB, MC, MG, MM†, MM±, MS, MVTP, NA, NHR, NK, PK, RAAC, RAD, RG, SAM, SFA, SM±, SO, TLV, VF, WP. Sampling: A-SK, AA, AAA, AAS, AD, AE, AEOO, AF, AG, AH, AI, AK†, AKS, AKS†, AL, AMO, AO, AP, AR†, AS‡, AV, AVG, AvG, BB, BH, BK, BK†, BM, BN, BO, BT, CA, CD, CP, CS, CW, DB, DC, DD, DG, DGA, DN, DS, EKL, EM, EMO, EN, EP, ES, FA, FA‡, FE, FM, FM†, FO, FT, FT†, GAA, GG, GM, GPM, GT, GvZ, HC, HE, HN, IC, IG, IG†, IK, IM, IO, IS, JA, JCM, JD, JES, JG, JG†, JJL, JK, JL, JMH, JMM, JN, JN†, JTK, KA, KMS, LB, M-MD, MA†, MC, MD, MeH, MGS, MKK, MM§, MM†, MM±, MMD, MN, MO†, MOA, MR, MS, MWM, MY, NA, NG, NH, NHR, NI, NK, NM, NN, NS, NS†, OC†, OEC, OF, OF†, OI, OJ, OK, OO, OP, OS, OT†, P, PB, PC, PCS, PD, PK, PKQ, PO, PS, RAD, RG, RN, SA, SA†, SB, SBL, SD, SE, SeK, SFA, SG†, SK, SL, SLD, SM, SM†, SMM, SN, SP†, SS, ST, TLV, TM, TS, UC, UG, UJ, UR, VG, WA, WC, WP, WR, YB, YKT, YN, ZRD. Sequencing: A-SK, AA, AAA, AAS, AC, AD, AEOO, AF, AI, AI‡, AK, AK†, AKK, AKS, AKS†, AL, ANZ, AP, AS, AS†, AS‡, ASO, AT, AV, AVG, AvG, AY, BB, BD, BH, BK, BK†, BM†, BN, BT, CA, CB, CBP, CD, CMM, CP, CS, DB, DD, DG, DGA, DJB, DLB, DM, DOO, DP, DSYA, DT, EF, EFN, EKL, EL, EMO, EP, ES, ES†, ESL, FA, FA†, FAD, FD, FM, FM†, FO, FT†, FW, GAA, GG, GPM, GT, GvZ, HA, HA†, HC, HCR, HE, HG†, HK, HN, IB, IC, IG, IG†, IK, IM, IS, JA, JB, JCM, JD, JF, JG, JG†, JJL, JK, JMH, JMM, JMN, JN, JN†, JQM, JTK, JY, KA, KMS, KOD, KS, KT, LB, LF, LS, LT, M-MD, MA†, MAB, MC, MD, MeH, MGS, MIM, MKK, MM, MM§, MM‡, MMD, MMN, MO, MO†, MOA, MVTP, MWM, MY, ND, NG, NH, NI, NI†, NM, NN, NN†, NS, NS†, NT, OC, OC†, OEC, OF, OI, OJ, OT†, PA, PB, PCS, PD, PEO, PK, PKQ, PM, PO, PS, RAAC, RG, RN, ROP, SA, SA†, SB, SBL, SCS, SD, SE, SE, SeK, SG, SG†, SHA, SK†, SL, SLD, SM, SM†, SM±, SMM, SN, SP†, SP‡, SR, SS, ST, ST†, SvdW, TA, TM, TM†, TS, UC, UG, UJ, UJA, UR, VG, WA, WC, WR, YB, YB†, YKT, YN, ZRD. Visualization: AC, AI‡, AK, AKK, AS, AS†, AY, BT, CB, CMM, DB†, DOO, DP, DR, DSYA, EA, EB, ESL, EW, FAD, FB, FD, FW, GS, HA, HA†, HG†, HL, HT†, IA, IS, JAE, JB, JF, JG†, JMN, JY, KHY, KS, LF, LS, LT, MA, MA†, MG, MT, MVTP, MW, ND, NI†, NK, NN†, NT, OC, OT, PA, PCS, PEO, RAAC, SB, SFS, SHA, SK†, SM±, TA, TM†, VE, YB†. Funding acquisition: AJP, AR, AvG, BK, CA, CAK, CBP, CW, DC, DJB, DN, FL, GAA, GG, GPM, HC, JES, JJT, JL, JMH, JN‡, JO, KOD, M-MD, MC, MIM, MM±, MVTP, NA, PCS, PK, PM, RAK, SAM, SE, SM†, SvdW, TdO, WP. Project administration: AJP, AR, AV†, AvG, BK, CW, DJB, DN, EW, FA†, FT, GAA, GPM, GS, GT, HC, JCO, JJT, JMH, JO, JOG, JY, KOD, MC, MK, MM†, MP, MVTP, MW, NR, OT, PCS, PK, PM, RAK, SAM, SE, SFS, SG†, SM†, TdO. Supervision: AJP, AR, BK, CW, DN, EN, EW, FT, GAA, GK, HC, JB, JMH, JN‡, JO, JOG, KOD, MA†, MC, MIM, MM†, MMN, MS, NM†, NR, PCS, PK, PM, RAK, SE, SeK, SG†, SM, SM†, SP, TdO. Writing – original draft: AKS, ANZ, BK, DPM, EW, FT, GK, HT†, JB, JCM, MA†, MAB, MC, MG, MM, NM†, RL. Writing – review and editing: ANZ, BK, CMM, DN, DPM, DR, DSYA, DT, EKL, EL, ESL, EW, HT†, JES, JGS, LdOM, MAB, MC, MeH, PKQ, PM, RL, SKT, TdO, UJA. *Author's contributions listed alphabetically. A full list of author abbreviations is included on the GitHub deposit (https://github.com/krisp-kwazulu-natal/africa-covid19-genomics) (*23*). **Competing interests:** Dr. Pardis Sabeti is a founder and shareholder of Sherlock biosciences, and is both on the Board and serves as shareholder of the Danaher Corporation. The authors declare no other conflicts of interest. **Data and materials availability:** All sequences that were used in the present study are listed in table S4 (accessible on the GitHub repository) along with their GISAID sequence IDs, dates of sampling, the originating and submitting laboratories and main authors. All input files (e.g., alignments or XML files), all resulting output files and scripts used in the study are shared publicly on GitHub (https://github.com/krisp-kwazulu-natal/africa-covid19-genomics) (*23*). This work is licensed under a Creative Commons Attribution 4.0 International (CC BY 4.0) license, which permits unrestricted use, distribution, and reproduction in any medium, provided the original work is properly cited. To view a copy of this license, visit https://creativecommons.org/licenses/by/4.0/. This license does not apply to figures/photos/artwork or other content included in the article that is credited to a third party, obtain authorization from the rights holder before using such material.

SUPPLEMENTARY MATERIALS

https://science.org/doi/10.1126/science.abj4336
Materials and Methods
Figs. S1 to S10
Tables S1 to S4
References (*24*–*38*)
MDAR Reproducibility Checklist

12 May 2021, accepted 3 September 2021
Published online 9 September 2021
10.1126/science.abj4336

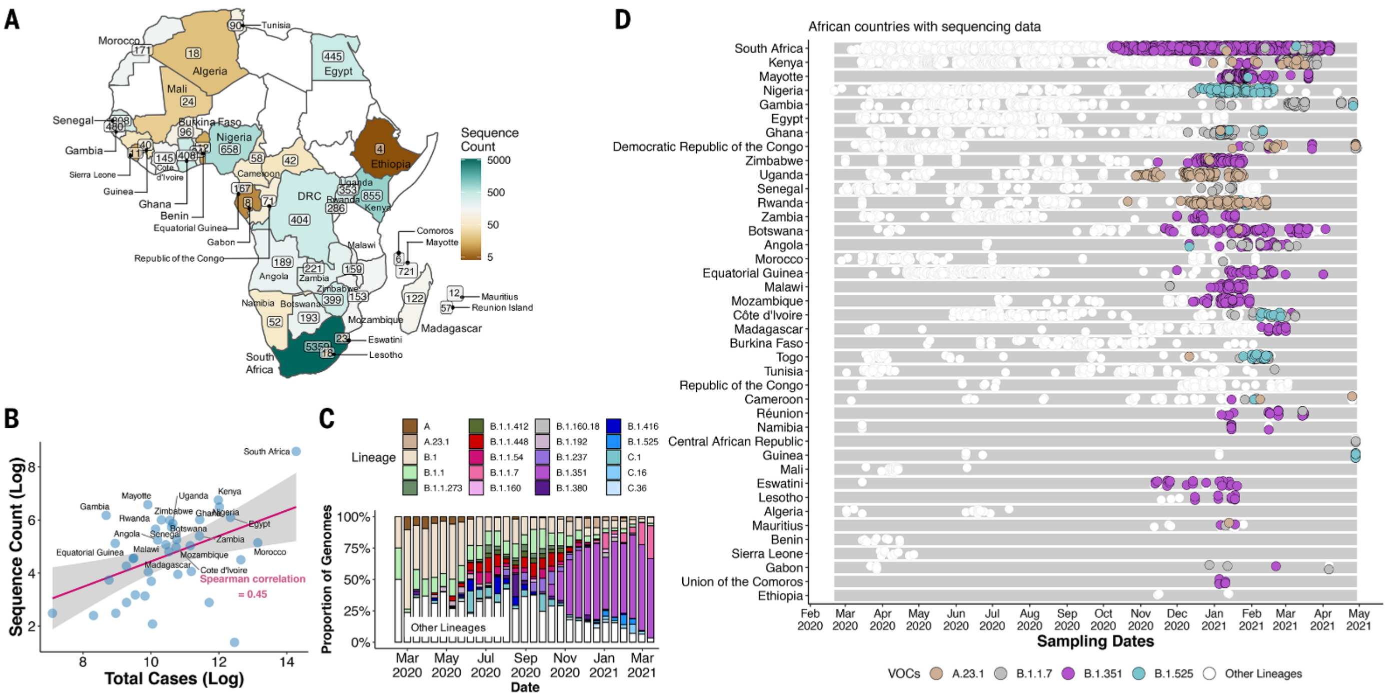

Fig. 1. SARS-CoV-2 sequences in Africa. (**A**) Map of the African continent with the number of SARS-CoV-2 sequences reflected in GISAID as of 5 May 2021. (**B**) Regression plot of the number of viral sequences vs. the number of reported COVID-19 cases in various African countries as of 5 May 2021. Countries with >500 sequences are labeled. (**C**) Progressive distribution of the top 20 PANGO lineages on the African continent. (**D**) Temporal sampling of SARS-CoV-2 sequences in African countries (ordered by total number of sequences) through time with VOCs of note highlighted and annotated according to their PANGO lineage assignment.

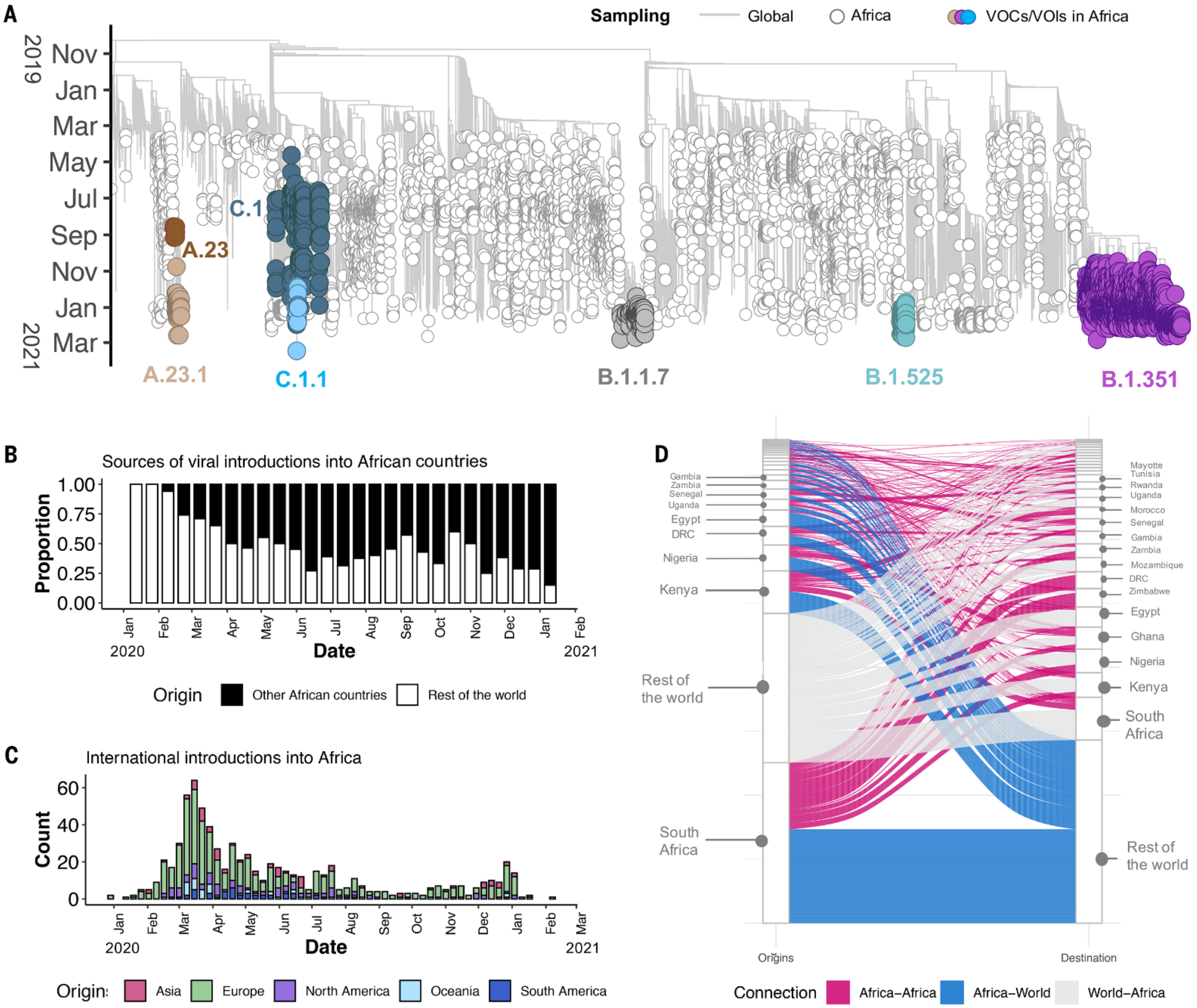

Fig. 2. Phylogenetic reconstruction of the SARS-CoV-2 pandemic on the Africa continent. (**A**) Time resolved Maximum Likelihood tree containing 8,746 high quality African SARS-CoV-2 near-full-genome sequences analyzed against a backdrop of global reference sequences. Variants of interest (VOI) and concern (VOC) are highlighted on the phylogeny. (**B**) Sources of viral introductions into African countries characterized as external introductions from the rest of the world vs internal introductions from other African countries. (**C**) Total external viral introductions over time into Africa. (**D**) The number of viral imports and exports into and out of various African countries depicted as internal (between African countries in pink) or external (between African and non-African countries in blue and grey).

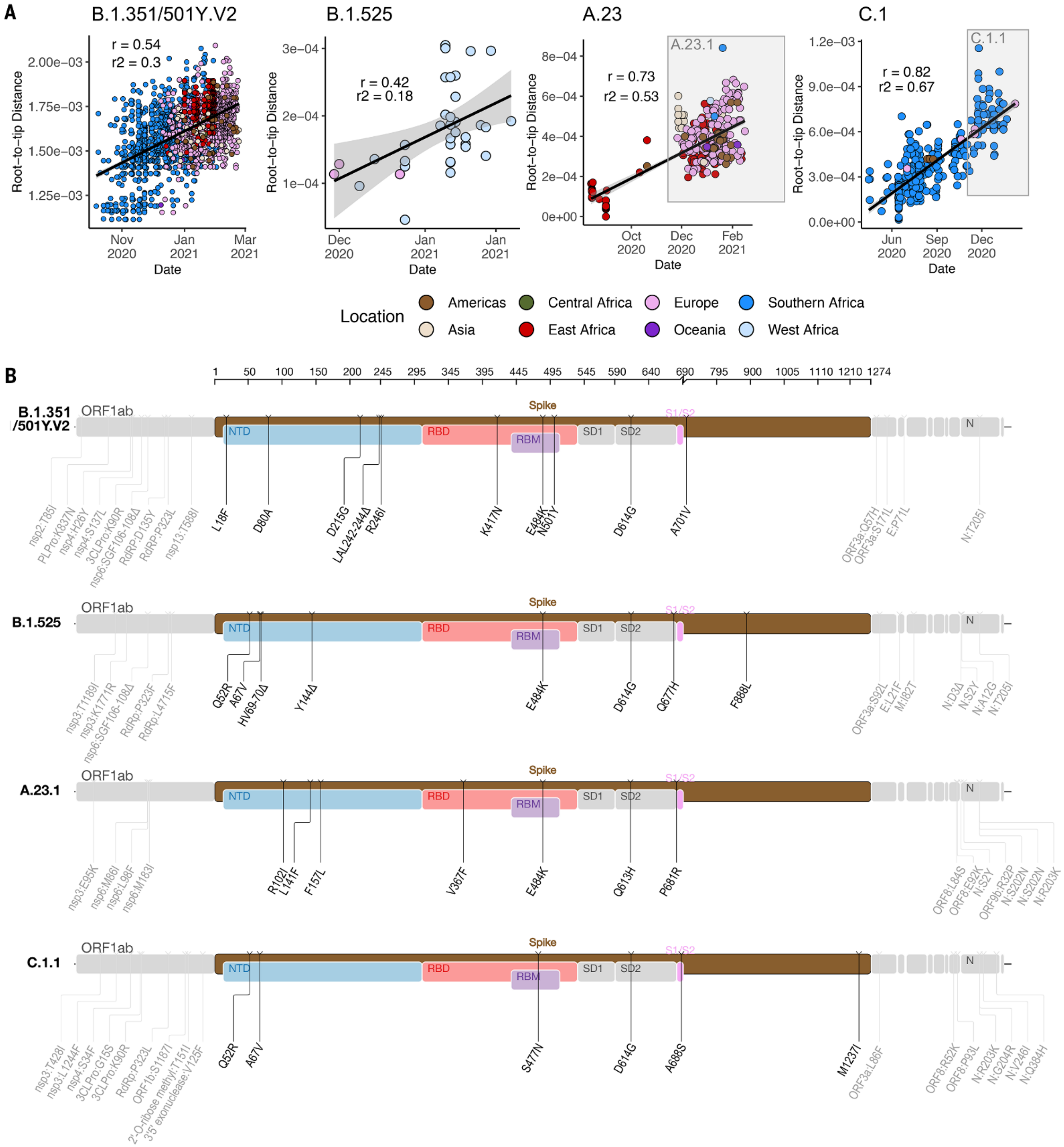

Fig. 3. Genetic profile of VOCs and VOIs under investigation. (**A**) Root-to-tip regression plots for four lineages of interest. C.1 and A.23 show continued evolution into VOIs C.1.1 and A.23.1 respectively. (**B**) Genome maps of four VOCs/VOIs where the spike region is shown in detail and in color and the rest of the genome is shown in grey. ORF: open reading frame, NTD: N-terminal domain, RBD: receptor binding domain, RBM: receptor binding motif, SD1: subdomain 1 and SD2: subdomain 2.

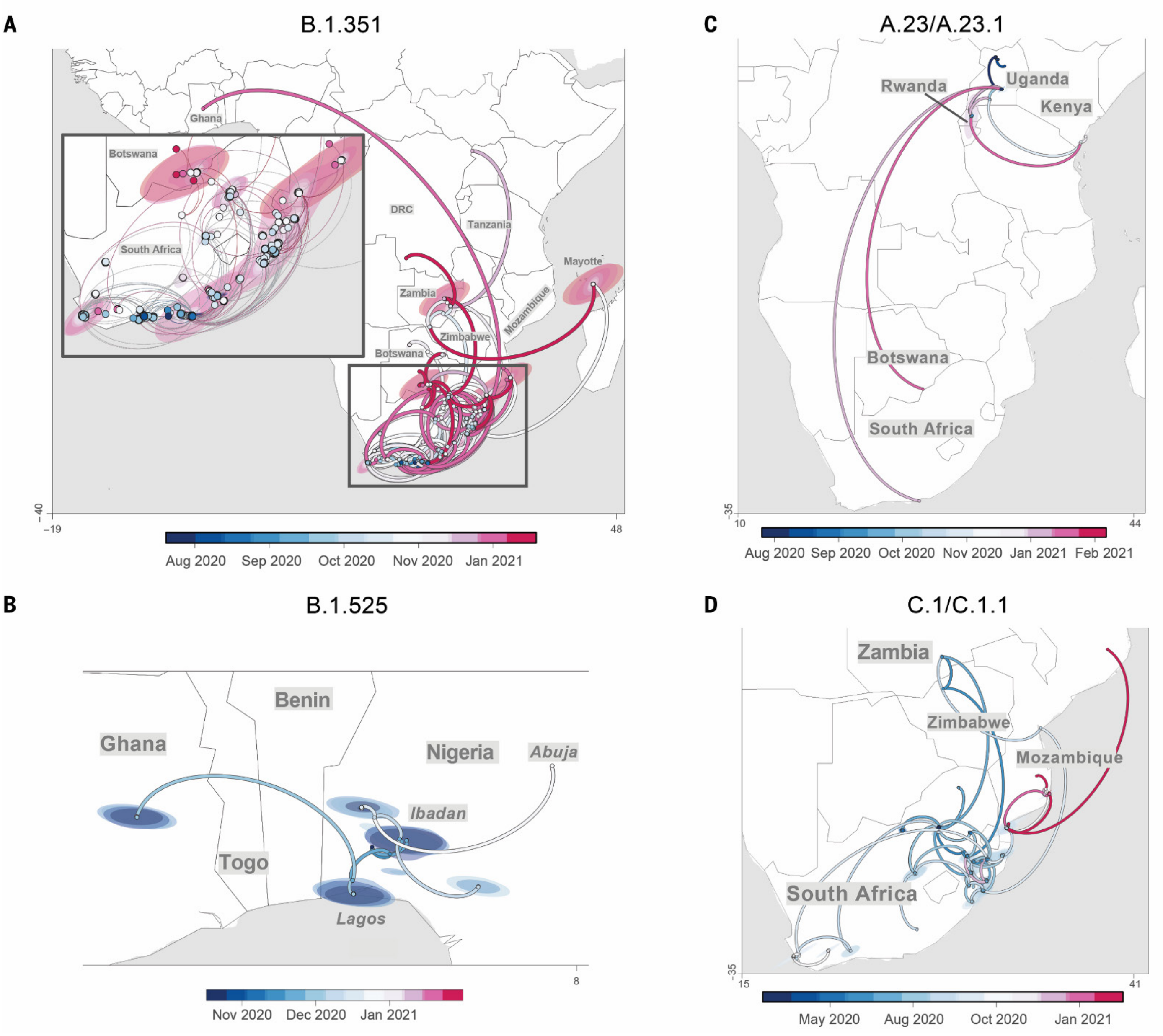

Fig. 4. Phylogeographic reconstruction of the spread of four VOCs/VOIs across the African continent using sequences showing strict continuous transmission across geographical regions. (**A** to **D**) Curved lines denote the direction of transmission in the anti-clockwise direction. Solid lines show transmission paths as inferred by phylogeographic reconstruction and colored by date, whereas dashed lines show known travel history of the particular case considered.
