# Supplementary material for: A year of genomic surveillance reveals how the SARS-CoV-2 pandemic unfolded in Africa
Source: Science. 2021 Sep 9;374(6566):423–31. doi: 10.1126/science.abj4336 (PMC7613315; doi:10.1126/science.abj4336)
Supplement: Supplementary file 2 — Materials and Methods Figs. S1 to S10 Tables S1 to S3 References (24–38) [file science.abj4336_sm.pdf]

Science
AAAS

Supplementary Materials for

A year of genomic surveillance reveals how the SARS-CoV-2 pandemic unfolded in Africa

Eduan Wilkinson *et al.*

Corresponding author: Tulio de Oliveira, tulio@sun.ac.za

Science **374**, 423 (2021)
DOI: 10.1126/science.abj4336

The PDF file includes:

Materials and Methods
Figs. S1 to S10
Tables S1 to S3
References

Other Supplementary Material for this manuscript includes the following:

Table S4
MDAR Reproducibility Checklist

Materials and Methods

Ethics statement

This project relied on sequence data and associated metadata publicly shared by the GISAID data repository and adhere to the term and conditions laid out by GISAID. The African samples processed in this study were obtained anonymously from material exceeding the routine diagnosis of SARS-CoV-2 in African public health laboratories that belong to the public network within the Africa CDC. Individual institutional review board (IRB) references or material transfer agreements (MTAs) for countries are list below.

Angola - (MTA - CON8260), Botswana - Genomic surveillance in Botswana was approved by the Health Research and Development Committee (Protocol HPDME 13/18/1), Nigeria – (NHREC/01/01/2007), Mali - study of the sequence of SARS-CoV-2 isolates in Mali - Letter of Ethical Committee (N0-2020 /201/CE/FMPOS/FAPH of 09/17/2020), Mozambique - (MTA - CON7800), Malawi - (MTA - CON8265), South Africa - The use of South African samples for sequencing and genomic surveillance were approved by University of KwaZulu-Natal Biomedical Research Ethics Committee (ref. BREC/00001510/2020); the University of the Witwatersrand Human Research Ethics Committee (HREC) (ref. M180832); Stellenbosch University HREC (ref. N20/04/008_COVID-19); and the University of Cape Town HREC (ref. 383/2020), Tunisia - For sequences derived from sampling in Tunisia, all patients provided their informed consent to use their samples for sequencing of the viral genomes. The ethical agreement was provided to the research project ADAGE (PRFCOVID19GP2) by the Committee of protection of persons (Tunisian Ministry of Health) under the reference (CPP SUD N 0265/2020), Uganda - The use of samples and sequences from Uganda were approved by the Uganda Virus Research Institute - Research and Ethics Committee UVRI-REC Federalwide Assurance [FWA] FWA No. 00001354, study reference - GC/127/20/04/771 and by the Uganda National Council for Science and Technology, reference number - HS936ES) and Zimbabwe (MTA - CON8271).

Data quality control

10326 African complete and near-complete genome sequences were retrieved from GISAID on 16 March 2021 (2pm SAST). Sampling strategies in various participating countries are outlined in Supplementary Table S3. Prior to phylogenetic reconstruction we removed low quality sequences, which included those identified as being of low quality by NextClade (n=18; https://clades.nextstrain.org), those with missing sampling dates (n = 189), those with <90% coverage (n = 1017), those with > 40 SNPs (n = 39), those with >10 ambiguous base-calls per genome (n = 128), and those with clustered SNPs (n = 189).

High quality African near-complete genome sequences (n=8,746) were aligned against an extensive reference dataset of 11891 SARS-CoV-2 sequences from around the world that included sequences sampled since the start of the outbreak, including all those sampled up until the end of February 2020.

Phylogenetic reconstruction

The African sequences were aligned against the reference panel using MAFFT v7.471(*24*). The first 100 and last 50 bases as well as positions 13402, 24389 and 24390 relative to the reference strain Wuhan-Hu-1 (*18,(25)* were masked as these three sites are known for primer contamination resulting in ambiguity. The subsequent alignment was used to infer a maximum likelihood (ML) phylogenetic tree in IQTREE v1.6.9(*26*). The tree was inferred with the general time reversible (GTR) model of nucleotide substitution and a proportion of invariable sites (+I). To infer some confidence measures of branches in the phylogeny and for subsequent downstream analyses we performed 100 bootstrap replicates using Booster(*27*).

The raw ML tree topology was used to estimate the number of viral transmission events between various Africa countries and the rest of the world. TreeTime(*28*) was used to transform this ML tree topology into a dated tree using a constant rate of 8.0 x 10^{-4} nucleotide substitutions per site per year , after the exclusion of

outlier sequences. A migration model was fitted to the resulting time-scaled phylogenetic tree in TreeTime, mapping country and regional locations to tips and internal nodes. Using the resulting annotated tree topology we could count the number of transitions between Africa and the rest of the world.

Lineage classification

We used the dynamic lineage classification method called Phylogenetic Assignment of Named Global Outbreak LINeages (PANGOLIN)(*29*). This was aimed at identifying the most epidemiologically important lineages of SARS-CoV-2 circulating within the African continent and to identify the lineage dynamics within African regions and across the continent. For the purpose of clarity, we define a lineage as a linear chain of viruses in a phylogenetic tree showing connection from the ancestor to the most recent descendant. A unique variant refers to a genetically distinct virus with different mutations to other viruses of the same lineage. Variants of concern (VOC) and variants of interest (VOI) were designated based on the World Health Organization framework as of 13 April 2021. We included two other lineages, namely A.23.1 and C.1.1, and designated them as VOI for the purposes of this analysis. We included these two as they demonstrated continued evolution of African lineages into potentially more transmissible variants with the acquisition of mutations in the spike glycoprotein.

Phylogeographic reconstruction

VOCs and VOIs that emerged on the African continent (B.1.351, B.1.525, A.23.1 and C.1.1) were marked on the time-resolved phylogenetic tree constructed above. Genome sequences from these four lineages were extracted for phylogeographic reconstruction. First, we investigated the dynamics of SARS-CoV-2 infection and virus lineage movements over longer distances (through Europe or East to West Africa) using a sampled set of time-scaled phylogenies and the sampling location of each geo-referenced SARS-CoV-2 sequence. We discretized sequence sampling locations by considering distinct geographic areas and/or regions (in and outside Africa) as shown in Supplementary Figure S6.

Initially, discrete phylogeographic reconstructions were conducted for all VOC and VOI using the asymmetric discrete trait model implemented in BEASTv1.10.4(*30*). From those estimates we then modelled the phylogenetic diffusion and spread of the lineages on the African continent by analysing localized transmission (between neighbouring countries) using a flexible relaxed random walk (RRW) diffusion model(*31*) that accommodates branch-specific variation in rates of dispersal with a Cauchy distribution. For each sequence, latitude and longitude coordinates were attributed to the lowest administrative level locator in GISAID.

Multiple sequence alignments were performed for each lineage with MAFFT v7.471. Maximum likelihood trees for each of the alignments were inferred in IQTREE v1.6.9 (GTR+I). Prior to phylogeographic reconstruction each cluster/lineage was assessed for molecular clock signal in TempEst v1.5.3(*32*) following the removal of potential outliers that may violate the molecular clock assumption. Markov Chain Monte Carlo (MCMC) analyses were set up in BEAST v1.10.4 in duplicate for 100 million interactions and sampling every 10000 steps in the chain. Convergence for each run was assessed in Tracer v1.7.1 (ESS for all relevant model parameters >200). Maximum clade credibility trees for each run were summarized using TreeAnnotator after discarding the initial 10% as burn-in. We used the R package “seraphim”(*33*) to extract and map spatiotemporal information embedded in the posterior trees. Note that a transmission link on the phylogeographic map can denote one or more transmission events depending on the phylogeographic inference.

Sensitivity of introduction analysis to sampling biases

Three sensitivity analyses were performed to examine how robust the main results of our introduction analysis were to known biases in sampling across space and time. For our first analysis, we randomly selected 10 of the bootstrap tree topologies that was inferred using Booster for discrete state ancestral state reconstruction

as described earlier. The average number of imports and exports between Africa and the rest of the world per week were then plotted overtime along with the standard error for each discrete time point.

In the second, we performed a rarefaction analysis to determine how the number of introductions into Africa varies depending on the extent of sampling in African (internal) and non-African (external) countries. Rarefaction was performed by starting with the full set of samples and subsampling a random subset of samples from the full set at sampling fractions varying from 0.1 to 1.0. Subsampling was performed 10 times at each sampling fraction to create replicate datasets, which were used to place confidence internals on the number of introductions identified at each subsampling fraction.

Because it would have been too computationally intensive to reconstruct phylogenies *de novo* from each subsampled dataset, we adopted a subsample-then-prune approach(*34*). For each subsampled dataset, samples not included in the subsampled set were pruned from the full ML phylogeny using the *extract_tree_with_taxa* function in DendroPy version 4.5.1(*35*). Ancestral locations were then reconstructed for internal nodes in each subsampled or pruned tree using maximum parsimony(*36*). The total number of introductions into Africa was then computed based on the number of branches in the tree in which the parent node was reconstructed to be external and the child node was reconstructed to be in Africa.

The second analysis was performed to determine how sensitive the temporal distribution of introduction events was to uneven sampling through time. Perhaps most importantly, we sought to determine if the increasing proportion of introductions estimated to be from other African countries through time was an artefact of increased sampling effort during late 2020 and early 2021. To obtain a more uniform temporal distribution of sampling times, we capped the number of samples from Africa each month at a maximum threshold (n=400) and then randomly down-sampled to this threshold count in months that exceeded the threshold. As in the rarefaction analysis, samples excluded after subsampling were pruned from the ML tree after which ancestral states were reconstructed by maximum parsimony.

Epidemiological modelling
Data on regional trade of all imported and exported goods between South Africa and other Eastern and Southern African countries during 2020 was extracted from the United Nations Comtrade Database(*37*), which records trade statistics for more than 5,000 commodity groups by the Harmonized System. Data for cumulative COVID-19 cases and related deaths, vaccinated people, and cumulative numbers of COVID-19 tests performed by March 30, 2021 were obtained from the Johns Hopkins University database(*38*). Country level maps of each variable were created using ArcGIS® by ESRI version 10.5 (http://www.esri.com).

Supplementary Figures & Tables

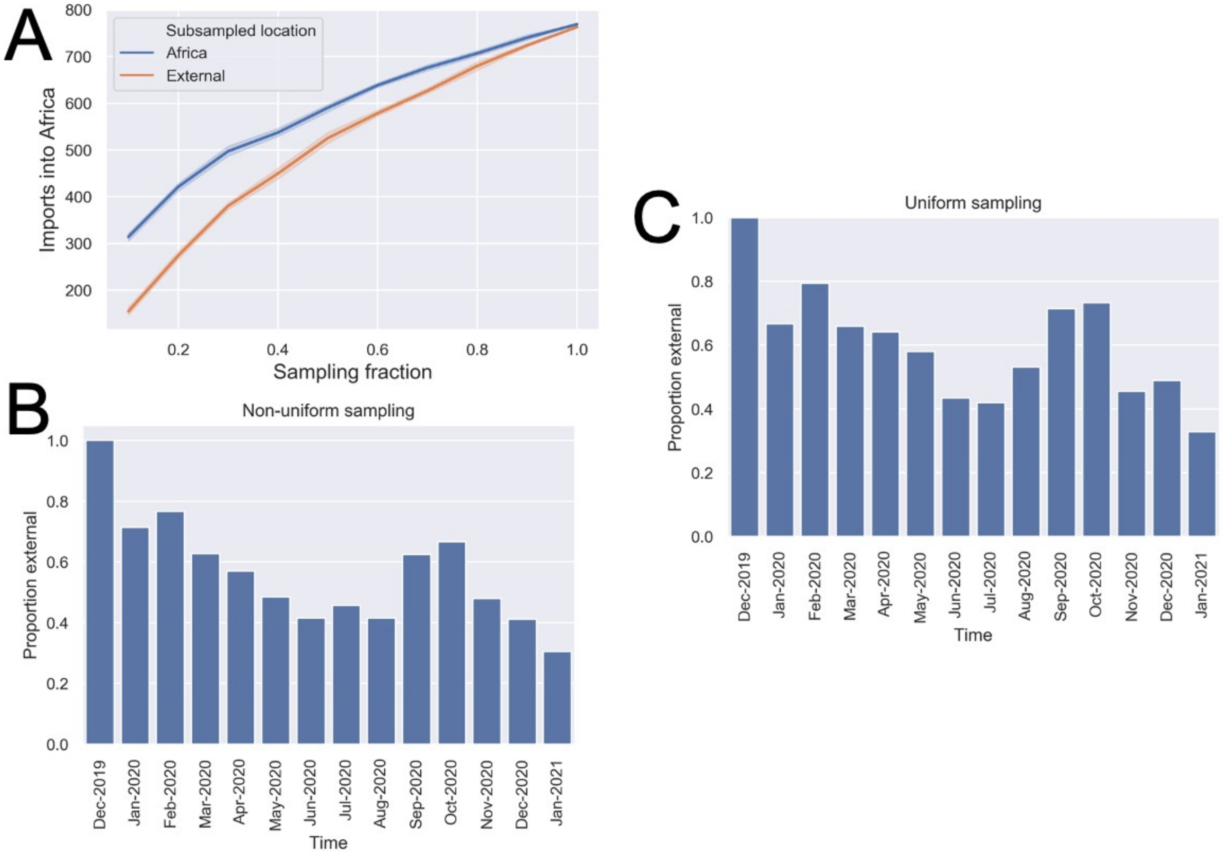

Supplementary Figure S1: *Sensitivity of the viral introduction analysis to geographic sampling biases. (A) A rarefaction analysis showing how the number of imports into Africa depends on the extent of sampling in Africa (blue) and the extent of external sampling in the rest of the world (orange). At each sampling fraction, a random set of samples was subsampled from the full dataset 10 times to create bootstrap replicates from which confidence intervals (shaded intervals) on the number of imports were computed. (B-C) Sensitivity analysis showing how the proportion of imports into African countries from external locations outside of Africa varied depending on the temporal distribution of samples in Africa. This analysis was performed twice with either non-uniform sampling through time using the same dataset as in Figure 2B-C of the main text (B) or uniform sampling through time in which we capped the number of samples from Africa at a maximum threshold of 400 each month.*

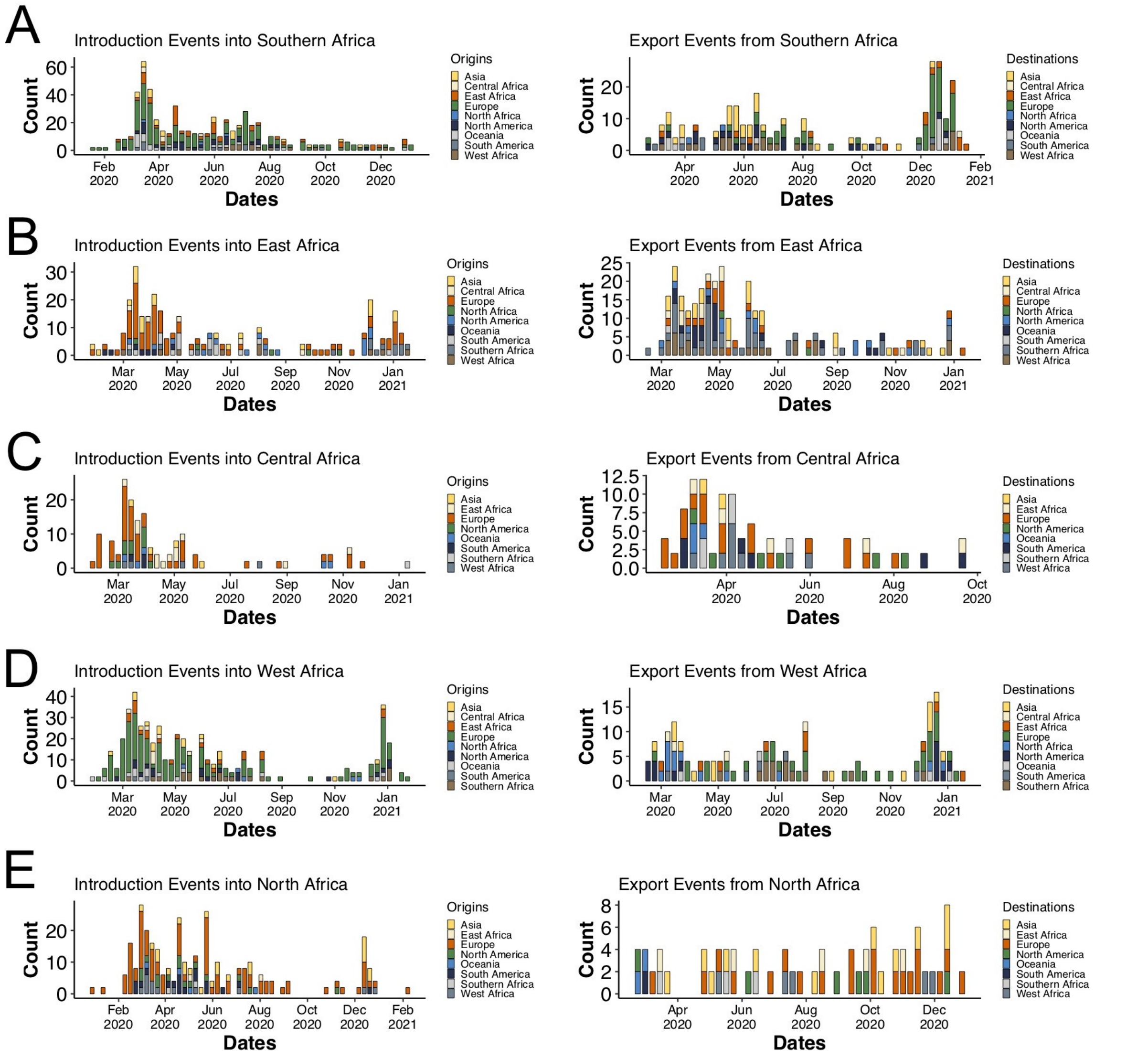

Supplementary Figure S2: *Number of importation and exportation events for various subregions on the African continent. African subregions are defined based on the African Union classification scheme.*

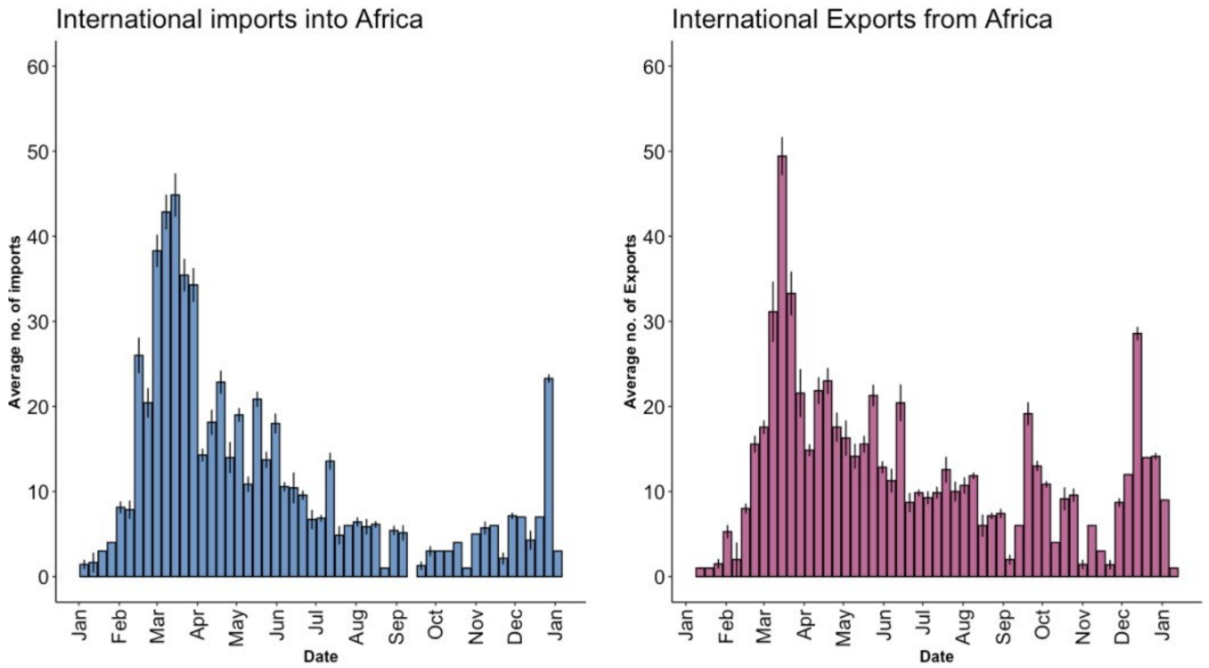

Supplementary Figure S3: *Numbers of importation and exportation events between Africa and the rest of the world over the first year of the SARS-CoV-2 pandemic.*

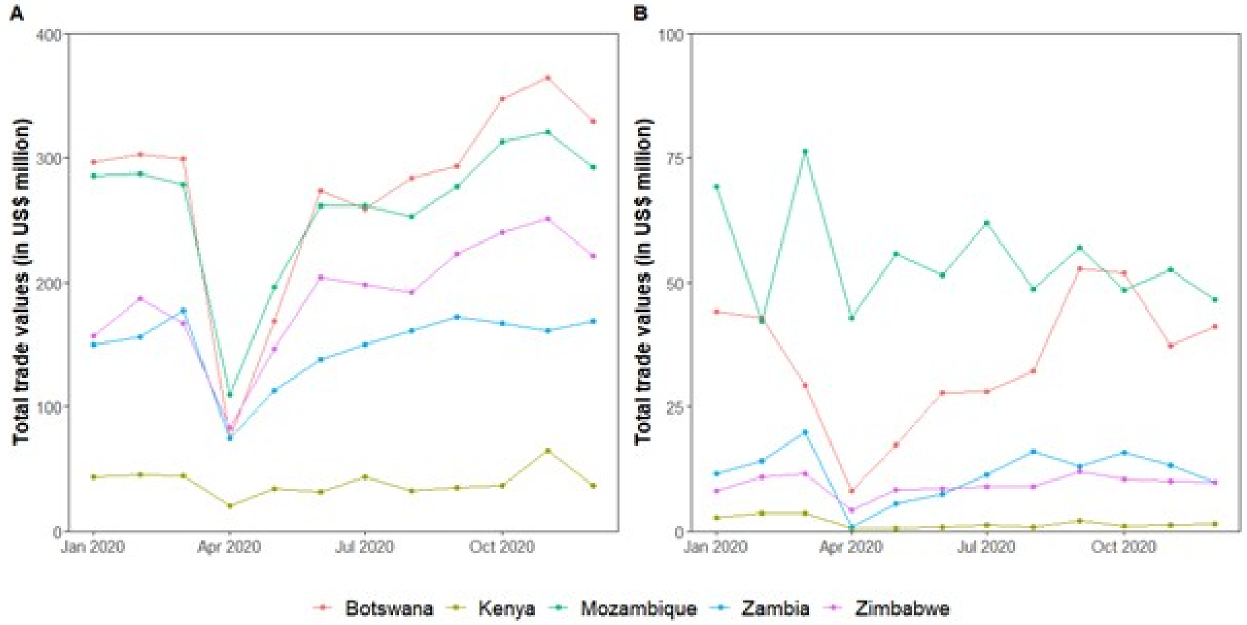

Supplementary Figure S4: *Total monthly international trade values in US million dollars in 2020 for A) exported goods from South Africa; and B) imported goods to South Africa with the following neighbouring countries: Botswana, Democratic Republic of the Congo, Eswatini, Lesotho, Malawi, Mozambique, Namibia, Zambia, and Zimbabwe. Source: UN Comtrade Database.*

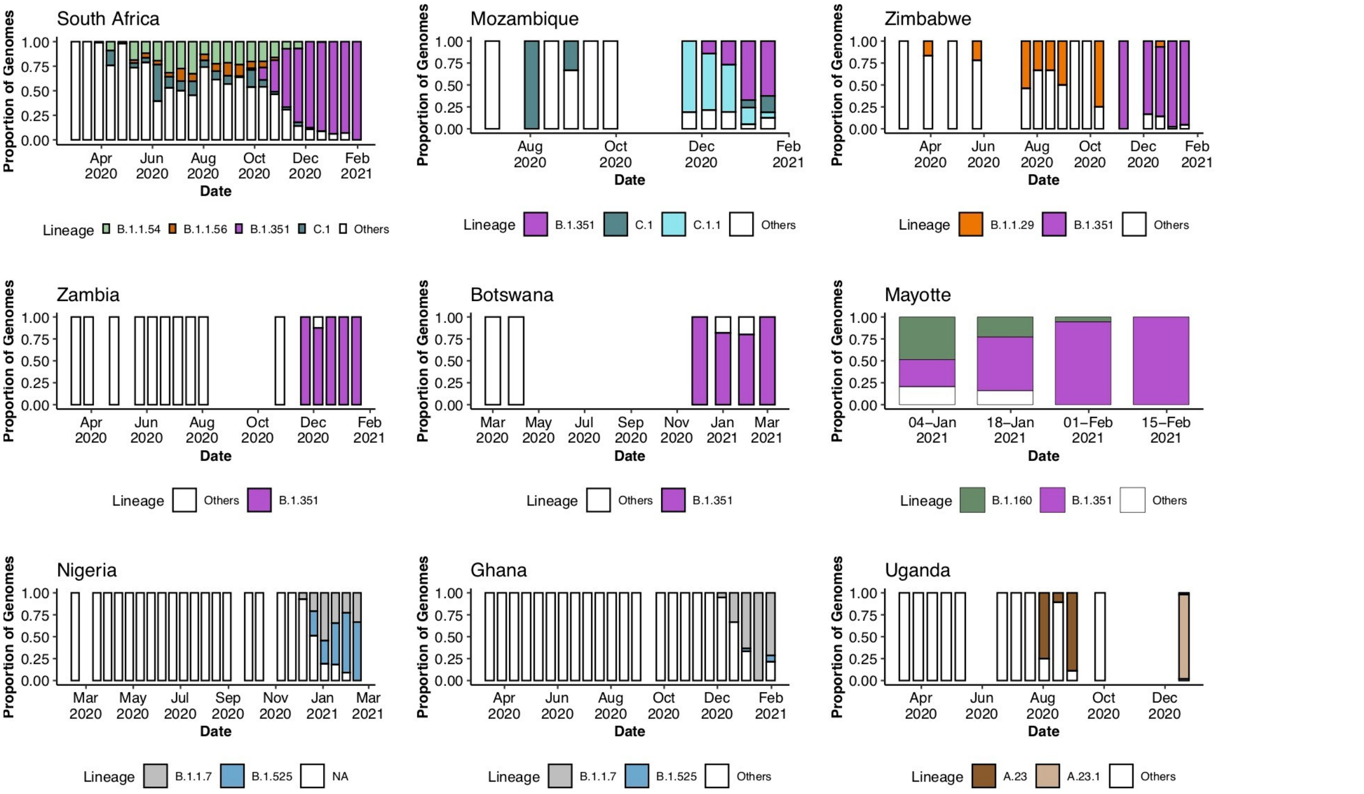

Supplementary Figure S5: *PANGO lineages through time for a select number of African countries.*

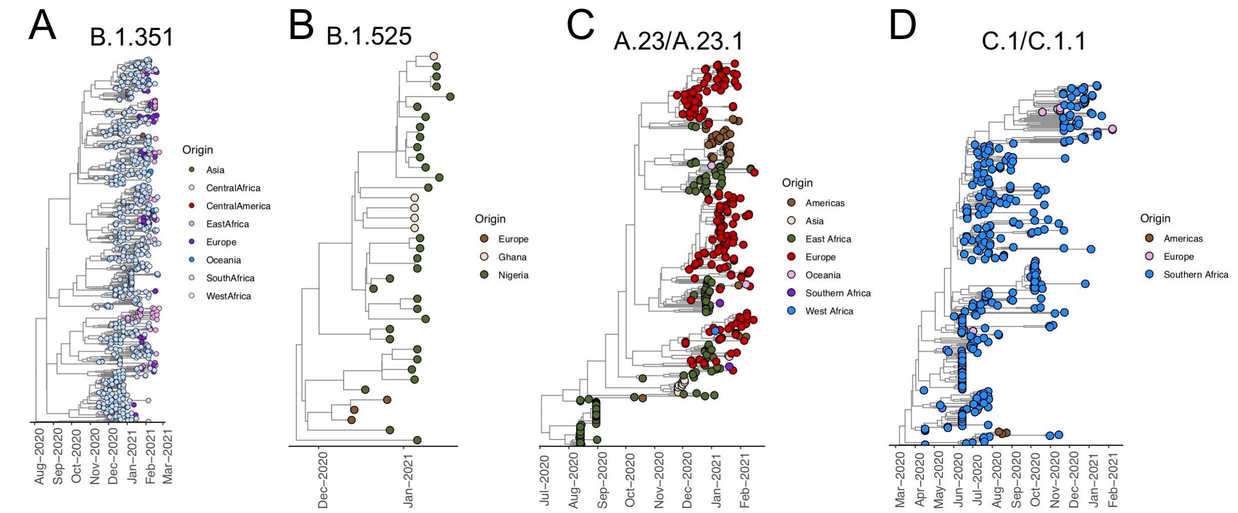

Supplementary Figure S6: *Maximum clade credibility phylogeographic trees including all global VOC or VOI samples. Branch colours represent most probable inferred locations of ancestral viruses. Numbers at internal nodes represent clade posterior probabilities.*

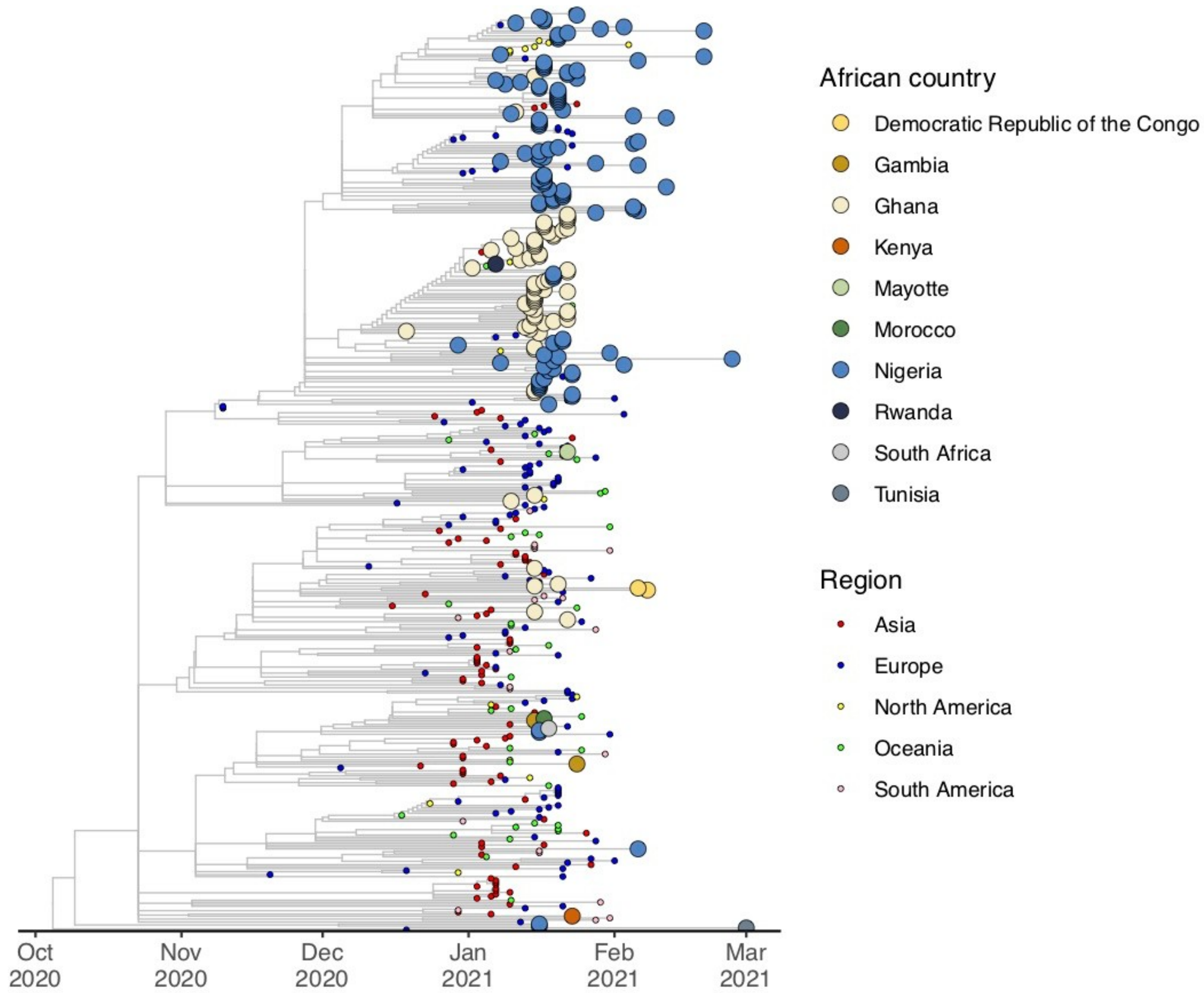

Supplementary Figure S7: *Time scaled phylogeny of the B.1.1.7 lineage. This phylogenetic cluster was extracted from the large dated phylogeny in Figure 2A. African sequences are highlighted by large circles, while non-African sequences appear as smaller dots. The branches are scaled in calendar time.*

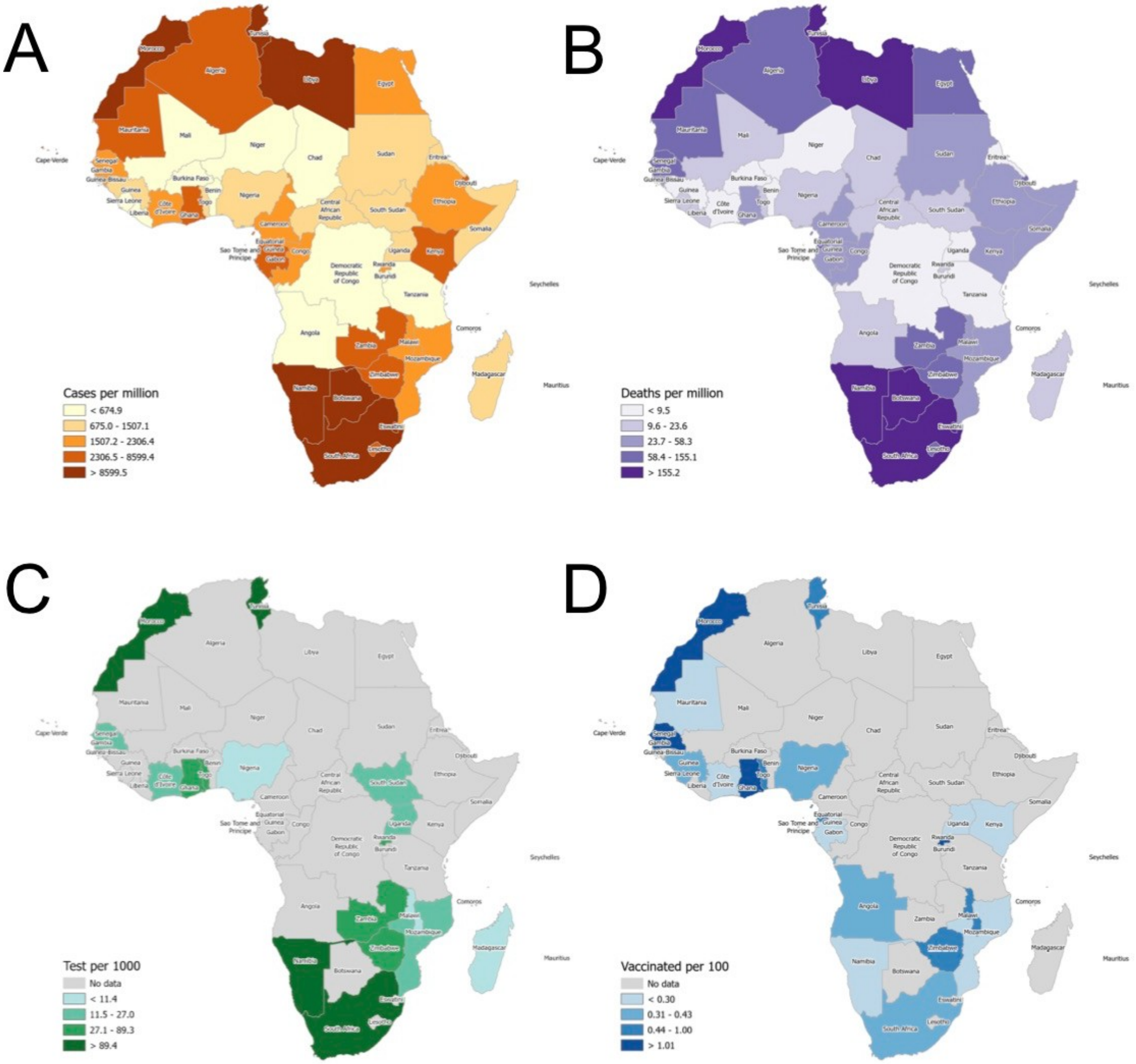

Supplementary Figure S8: *Epidemiological metricises of COVID-19 on the African continent. Clockwise from top left: reported COVID-19 cases per million individuals; reported COVID-19 attributed mortalities per million individuals; numbers of COVID-19 tests performed per 1,000 individuals; and numbers vaccinated per 100 individuals.*

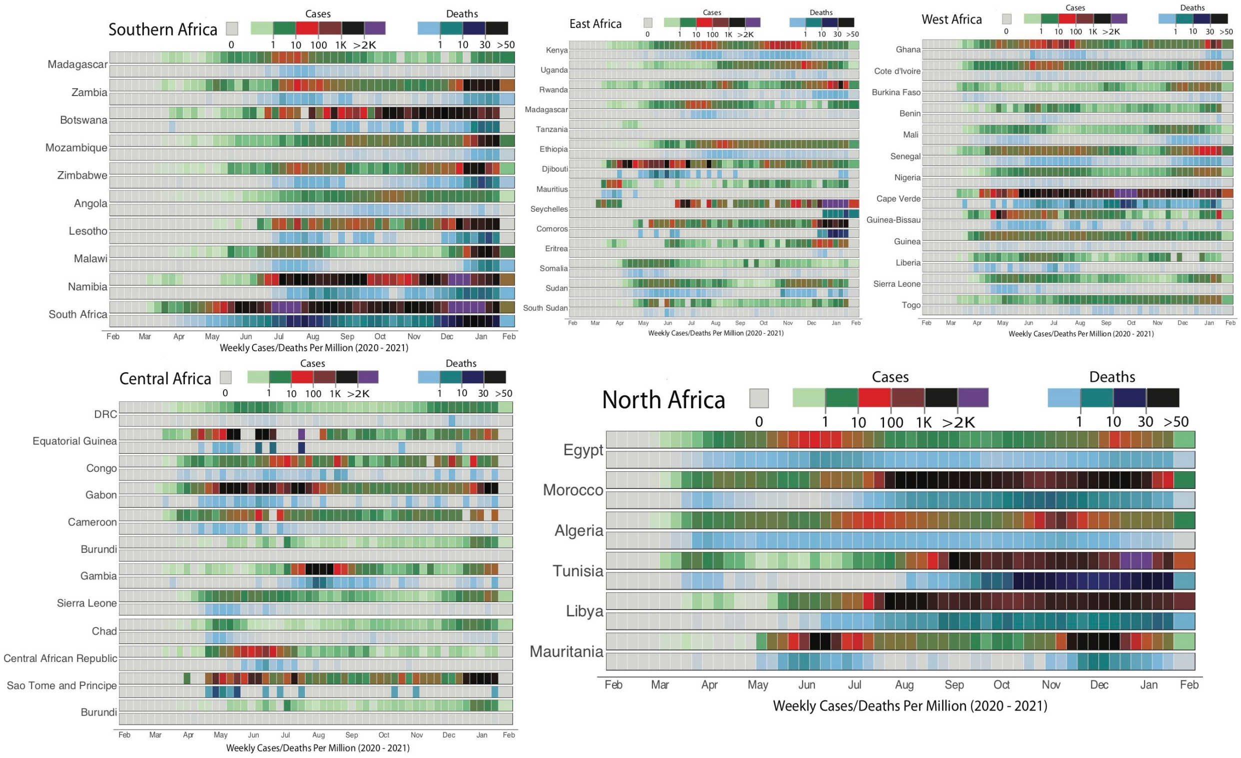

Supplementary Figure S9: *Epidemiological heatmaps of cases and deaths for various subregions on the African continent. African subregions are defined based on the African Union classification scheme.*

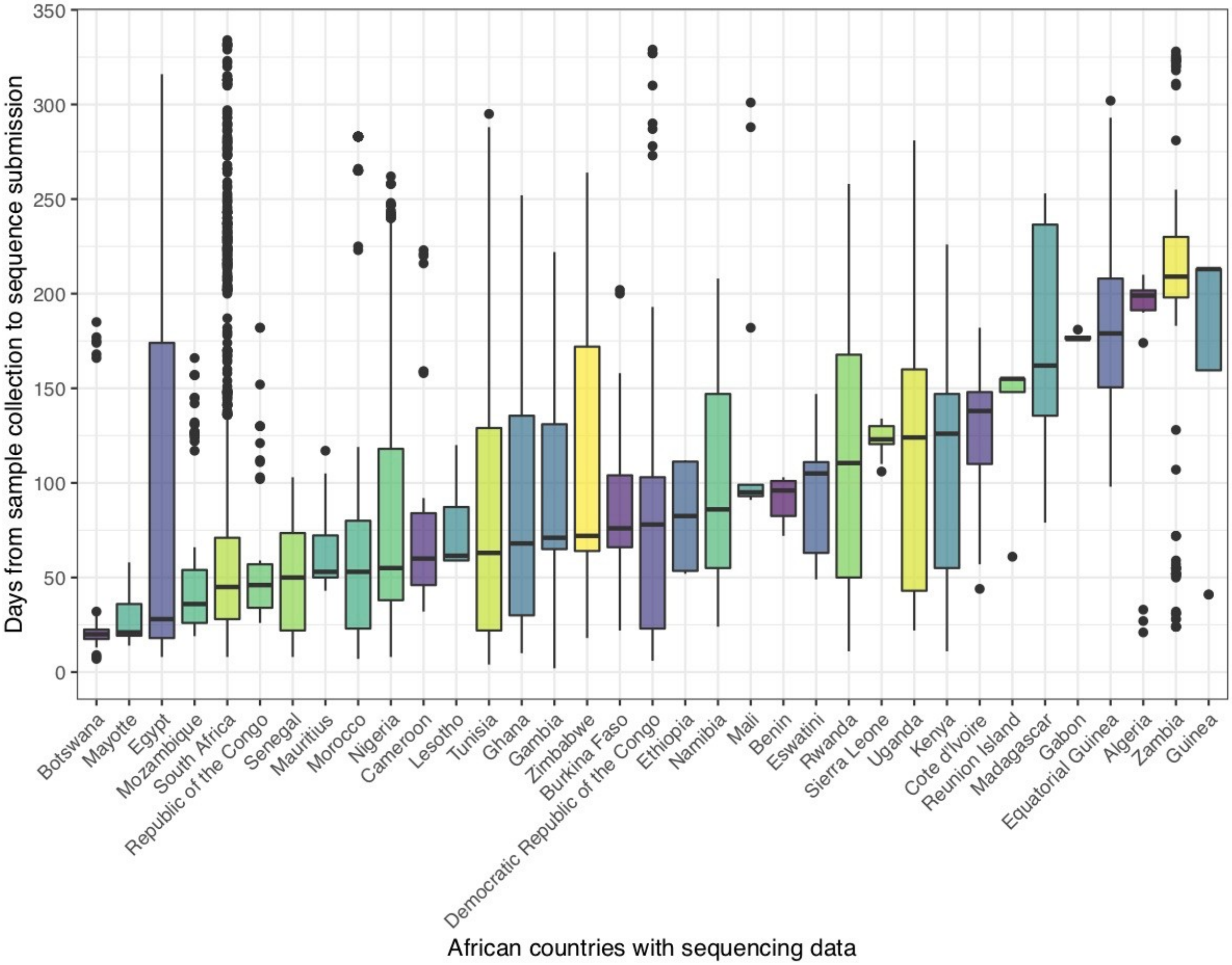

Supplementary Figure S10: *Graph of days from sampling to submission in various African countries.*

Supplementary Table S1. *Status and restrictions of land border posts in South Africa as of Feb 19, 2021.*

Country route	Number of land border posts		Restrictions
	Closed (n/N)	Open (n/N)	
South Africa - Botswana	13/17	4/17	· All passengers passing through the border posts are required to present a medical certificate with a negative COVID-19 test result issued within 72 hours or get tested upon arrival and subject to quarantine in a government holding facility. The entry to Zimbabwe requires a negative COVID-19 test result that is within 48 hours.<br>· Rail, ocean, air and road transport is permitted for the movement of cargo to and from other countries, subject to national legislation and any directions.<br>· All borders were closed on Jan 11, 2021 then reopened on February 15, 2021.
South Africa - eSwatini	6/11	5/11	
South Africa - Lesotho	7/13	6/13	
South Africa - Mozambique	2/4	2/4	
South Africa - Namibia	4/6	2/6	
South Africa - Zimbabwe	0/1	1/1	

Supplementary Table S2: *Variants of Concern/Note (VoC/Ns) in Africa.*

Variant Name	Lineage	Date Range	Spike Mutations of Biological Significance (all mutations)	Impact	Countries
N501Y.V2	B.1.351	Oct. 2020 – Feb. 2021	K417N, E484K, N501Y	Transmissibility, Escape Neutralization, ACE binding Affinity	South Africa, DRC, Mayotte, La Reunion, Zambia, Botswana, Congo, Kenya, Rwanda,
A.23, A.23.1	A.23.1	Dec. 2020 – Feb 2021	V367F, Q613H	Infectivity	Uganda, Rwanda, Ghana, South Africa, Zambia, Botswana
C.1.1	C.1.		S477N		Mozambique,
B.1.525	B.1.525	Dec. 2020 - Feb 2021	E484K, Q677H, F888L	Escape Neutralization, ACE binding Affinity	Nigeria, Ghana, Mayotte, Côte d'Ivoire/Bouaké Algeria
A.27/N501Y.V4	A.27	Jan 2021 - Feb 2021	L18F, L452R, N501Y, A653V, H655Y, Q677H, D796Y, G1219V	under investigation (VUI not VOC)	Mayotte, Europe, Ghana, Côte d'Ivoire/Bouaké
N501Y.V3					Brazil
B.1.160	B.1.160		D614G, S477N	confirmed reinfection (**under investigation**)	**Tunisia** (reinfection), Large European lineage Ghana
N501Y	B.1.1.7	Jan - Mash2021	D614G, N501Y, del69-70,	Transmissibility	**Ghana, Morocco Algeria,** Côte d'Ivoire/Bouaké, DRC

Supplementary Table S3: *Sampling or surveillance strategies in various participating institutions.*

Country	Proportion of cases sequenced	Sampling strategies				Other (details)
		**Regular surveillance (random sampling)**	**Cluster/outbreak investigations**	**Surveillance of imported cases (linked to border testing)**	**Investigation of re-infections**	
South Africa	0.20%	Yes	Yes	No	Yes	Sequencing of infections in vaccine trials Sequencing for health facility-based and community-based research projects
Zambia	0.27% (0.42%)	Yes	Yes	Yes	Yes	Not all investigations are being performed at all times. When cases exceed a particular threshold cluster, random and imported case surveillance reduces or stops. Total cases 8/2/21 = 63.573, 8/3/21 = 82,421.

Democratic Republic of Congo (DRC)	1.4 % (2.87%)	Yes	No	Yes	No	Regular surveillance is based on samples availability; the surveillance of imported cases is based on samples of travellers coming in DRC. there are also "sequencing based on a research project focused on respiratory infections (Andemia)
South Africa (FS)		Yes	No	No	No	All samples with Cts lower that 30 are stored (with storage record). From 5 districts samples are selected randomly on a week basis (10 - 30) per district. From the ~15 000 stored samples no repeat testing has been identified within less than 90 days.
Ghana (Uhas)	0.36% (0.12%)	Yes	Yes	No	No	Random surveillance based on clusters of cases. During periods of

						suspected widespread infections, cases are randomly selected and sequenced.
Tunisia	0.04%	Yes	No	No	Yes	Random surveillance. Cases are randomly selected and sequenced. Some suspected reinfection cases are now tested in Sfax (Tunisia).
Morocco		Yes	Yes	Yes	Yes	Sequencing of 10% of Sample that are positif for S drop real time PCR test using (taqPath kit from thermo) . Sanger Sequencing of the entire S gene for the confirmation of mutation related to new varriants. WGS for the genomic surveillance over time et geographical localtion.
Equatorial Guinea	3.10%	Yes	YES	Yes	No	During the first wave from March to August, all positive samples were stored and a

						random selection of these samples were sequenced.
Côte d'Ivoire (Bouaké)	24.30%	Yes	No	No	No	Data set includes all CoV-2 RT-PCR samples tested positive from surveillance in regions of Côte d'Ivoire other than Abidjan; testing at CHU Bouaké; sampling period May-November 2020. Currently generating sequences from samples collected between Dec 2020 and March 2021. Calculation of cases (collumn C): suspected cases: 1199; of those tested: 100%; of those tested positive: 268 (22.36%); of those sequenced: 65

Algeria	0,08%	Yes	Yes	Yes	No	Sequencing of Sample that are negatif for S by rRTPCR test using (taqPath kit from thermo) . Sanger Sequencing of the entire S gene for the confirmation of mutation related to new varriants. WGS for the genomic surveillance using MinION nanopore is in progress.
Mayotte		Yes	Yes	No	No	Random surveillance, with extra samples collections in case of

Supplementary Table S4: GISAID Acknowledgements Table supplied as an Excel attachment

References and Notes

1. C. Wang, P. W. Horby, F. G. Hayden, G. F. Gao, A novel coronavirus outbreak of global health concern. *Lancet* **395**, 470–473 (2020). doi:10.1016/S0140-6736(20)30185-9 Medline

2. Q. Li, X. Guan, P. Wu, X. Wang, L. Zhou, Y. Tong, R. Ren, K. S. M. Leung, E. H. Y. Lau, J. Y. Wong, X. Xing, N. Xiang, Y. Wu, C. Li, Q. Chen, D. Li, T. Liu, J. Zhao, M. Liu, W. Tu, C. Chen, L. Jin, R. Yang, Q. Wang, S. Zhou, R. Wang, H. Liu, Y. Luo, Y. Liu, G. Shao, H. Li, Z. Tao, Y. Yang, Z. Deng, B. Liu, Z. Ma, Y. Zhang, G. Shi, T. T. Y. Lam, J. T. Wu, G. F. Gao, B. J. Cowling, B. Yang, G. M. Leung, Z. Feng, Early transmission dynamics in Wuhan, China, of novel coronavirus-infected pneumonia. *N. Engl. J. Med.* **382**, 1199–1207 (2020). doi:10.1056/NEJMoa2001316 Medline

3. S. Uyoga, I. M. O. Adetifa, H. K. Karanja, J. Nyagwange, J. Tuju, P. Wanjiku, R. Aman, M. Mwangangi, P. Amoth, K. Kasera, W. Ng'ang'a, C. Rombo, C. Yegon, K. Kithi, E. Odhiambo, T. Rotich, I. Orgut, S. Kihara, M. Otiende, C. Bottomley, Z. N. Mupe, E. W. Kagucia, K. E. Gallagher, A. Etyang, S. Voller, J. N. Gitonga, D. Mugo, C. N. Agoti, E. Otieno, L. Ndwiga, T. Lambe, D. Wright, E. Barasa, B. Tsofa, P. Bejon, L. I. Ochola-Oyier, A. Agweyu, J. A. G. Scott, G. M. Warimwe, Seroprevalence of anti-SARS-CoV-2 IgG antibodies in Kenyan blood donors. *Science* **371**, 79–82 (2021). doi:10.1126/science.abe1916 Medline

4. L. Mwananyanda, C. J. Gill, W. MacLeod, G. Kwenda, R. Pieciak, Z. Mupila, R. Lapidot, F. Mupeta, L. Forman, L. Ziko, L. Etter, D. Thea, Covid-19 deaths in Africa: Prospective systematic postmortem surveillance study. *BMJ* **372**, n334 (2021). Medline

5. S. J. Salyer, J. Maeda, S. Sembuche, Y. Kebede, A. Tshangela, M. Moussif, C. Ihekweazu, N. Mayet, E. Abate, A. O. Ouma, J. Nkengasong, The first and second waves of the COVID-19 pandemic in Africa: A cross-sectional study. *Lancet* **397**, 1265–1275 (2021). doi:10.1016/S0140-6736(21)00632-2 Medline

6. P. Oluniyi, First African SARS-CoV-2 genome sequence from Nigerian COVID-19 case. Virological (2020); https://virological.org/t/first-african-sars-cov-2-genome-sequence-from-nigerian-covid-19-case/421.

7. M. A. Medhat, M. El Kassas, COVID-19 in Egypt: Uncovered figures or a different situation? *J. Glob. Health* **10**, 010368 (2020). doi:10.7189/jogh.10.010368 Medline

8. M. Allam, A. Ismail, Z. T. H. Khumalo, S. Kwenda, P. van Heusden, R. Cloete, C. K. Wibmer, P. S. Mtshali, F. Mnyameni, T. Mohale, K. Subramoney, S. Walaza, W. Ngubane, N. Govender, N. V. Motaze, J. N. Bhiman, SA-COVID-19 Response Team, Genome sequencing of a severe acute respiratory syndrome coronavirus 2 isolate obtained from a South African Patient with coronavirus disease 2019. *Microbiol. Resour. Announc.* **9**, e00572-20 (2020). doi:10.1128/MRA.00572-20 Medline

9. N. Haider, A. Y. Osman, A. Gadzekpo, G. O. Akipede, D. Asogun, R. Ansumana, R. J. Lessells, P. Khan, M. M. A. Hamid, D. Yeboah-Manu, L. Mboera, E. H. Shayo, B. T. Mmbaga, M. Urassa, D. Musoke, N. Kapata, R. A. Ferrand, P.-C. Kapata, F. Stigler, T. Czypionka, A. Zumla, R. Kock, D. McCoy, Lockdown measures in response to COVID-19 in nine sub-Saharan African countries. *BMJ Glob. Health* **5**, e003319 (2020).

doi:10.1136/bmjgh-2020-003319 Medline

10. S. C. Inzaule, S. K. Tessema, Y. Kebede, A. E. Ogwell Ouma, J. N. Nkengasong, Genomic-informed pathogen surveillance in Africa: Opportunities and challenges. *Lancet Infect. Dis.* **21**, e281–e289 (2021). doi:10.1016/S1473-3099(20)30939-7 Medline

11. D. L. Bugembe, M. V. T. Phan, I. Ssewanyana, P. Semanda, H. Nansumba, B. Dhaala, S. Nabadda, Á. N. O'Toole, A. Rambaut, P. Kaleebu, M. Cotten, A SARS-CoV-2 lineage A variant (A.23.1) with altered spike has emerged and is dominating the current Uganda epidemic. medRxiv 2021.02.08.21251393 (2021); https://doi.org/10.1101/2021.02.08.21251393.

12. J. Giandhari, S. Pillay, E. Wilkinson, H. Tegally, I. Sinayskiy, M. Schuld, J. Lourenço, B. Chimukangara, R. Lessells, Y. Moosa, I. Gazy, M. Fish, L. Singh, K. Sedwell Khanyile, V. Fonseca, M. Giovanetti, L. Carlos Junior Alcantara, F. Petruccione, T. de Oliveira, Early transmission of SARS-CoV-2 in South Africa: An epidemiological and phylogenetic report. *Int. J. Infect. Dis.* **103**, 234–241 (2021). doi:10.1016/j.ijid.2020.11.128 Medline

13. H. Tegally, E. Wilkinson, R. J. Lessells, J. Giandhari, S. Pillay, N. Msomi, K. Mlisana, J. N. Bhiman, A. von Gottberg, S. Walaza, V. Fonseca, M. Allam, A. Ismail, A. J. Glass, S. Engelbrecht, G. Van Zyl, W. Preiser, C. Williamson, F. Petruccione, A. Sigal, I. Gazy, D. Hardie, N. Hsiao, D. Martin, D. York, D. Goedhals, E. J. San, M. Giovanetti, J. Lourenço, L. C. J. Alcantara, T. de Oliveira, Sixteen novel lineages of SARS-CoV-2 in South Africa. *Nat. Med.* **27**, 440–446 (2021). doi:10.1038/s41591-021-01255-3 Medline

14. C. A. Pearson, T. W. Russell, N. Davies, A. J. Kucharski, Estimates of severity and transmissibility of novel SARS-CoV-2 variant 501Y.V2 in South Africa. CMMID Repository (2021); https://cmmid.github.io/topics/covid19/sa-novel-variant.html.

15. S. Cele, I. Gazy, L. Jackson, S.-H. Hwa, H. Tegally, G. Lustig, J. Giandhari, S. Pillay, E. Wilkinson, Y. Naidoo, F. Karim, Y. Ganga, K. Khan, M. Bernstein, A. B. Balazs, B. I. Gosnell, W. Hanekom, M.-Y. S. Moosa, R. J. Lessells, T. Oliveira, A. Sigal, Escape of SARS-CoV-2 501Y.V2 variants from neutralization by convalescent plasma. medRxiv 2021.01.26.21250224 (2021); https://doi.org/10.1101/2021.01.26.21250224.

16. S. A. Madhi, V. Baillie, C. L. Cutland, M. Voysey, A. L. Koen, L. Fairlie, S. D. Padayachee, K. Dheda, S. L. Barnabas, Q. E. Bhorat, C. Briner, G. Kwatra, K. Ahmed, P. Aley, S. Bhikha, J. N. Bhiman, A. E. Bhorat, J. du Plessis, A. Esmail, M. Groenewald, E. Horne, S.-H. Hwa, A. Jose, T. Lambe, M. Laubscher, M. Malahleha, M. Masenya, M. Masilela, S. McKenzie, K. Molapo, A. Moultrie, S. Oelofse, F. Patel, S. Pillay, S. Rhead, H. Rodel, L. Rossouw, C. Taoushanis, H. Tegally, A. Thombrayil, S. van Eck, C. K. Wibmer, N. M. Durham, E. J. Kelly, T. L. Villafana, S. Gilbert, A. J. Pollard, T. de Oliveira, P. L. Moore, A. Sigal, A. Izu, NGS-SA Group, Wits-VIDA COVID Group, Efficacy of the ChAdOx1 nCoV-19 Covid-19 vaccine against the B.1.351 variant. *N. Engl. J. Med.* **384**, 1885–1898 (2021). doi:10.1056/NEJMoa2102214 Medline

17. C. K. Wibmer, F. Ayres, T. Hermanus, M. Madzivhandila, P. Kgagudi, B. Oosthuysen, B. E. Lambson, T. de Oliveira, M. Vermeulen, K. van der Berg, T. Rossouw, M. Boswell, V. Ueckermann, S. Meiring, A. von Gottberg, C. Cohen, L. Morris, J. N. Bhiman, P. L. Moore, SARS-CoV-2 501Y.V2 escapes neutralization by South African COVID-19

donor plasma. *Nat. Med.* **27**, 622–625 (2021). doi:10.1038/s41591-021-01285-x Medline

18. Y. Shu, J. McCauley, GISAID: Global initiative on sharing all influenza data - from vision to reality. *Euro Surveill.* **22**, 30494 (2017). doi:10.2807/1560-7917.ES.2017.22.13.30494 Medline

19. E. Volz, V. Hill, J. T. McCrone, A. Price, D. Jorgensen, Á. O'Toole, J. Southgate, R. Johnson, B. Jackson, F. F. Nascimento, S. M. Rey, S. M. Nicholls, R. M. Colquhoun, A. da Silva Filipe, J. Shepherd, D. J. Pascall, R. Shah, N. Jesudason, K. Li, R. Jarrett, N. Pacchiarini, M. Bull, L. Geidelberg, I. Siveroni, I. Goodfellow, N. J. Loman, O. G. Pybus, D. L. Robertson, E. C. Thomson, A. Rambaut, T. R. Connor, COG-UK Consortium, Evaluating the effects of SARS-CoV-2 spike mutation D614G on transmissibility and pathogenicity. *Cell* **184**, 64–75.e11 (2021). doi:10.1016/j.cell.2020.11.020 Medline

20. B. Korber, W. M. Fischer, S. Gnanakaran, H. Yoon, J. Theiler, W. Abfalterer, N. Hengartner, E. E. Giorgi, T. Bhattacharya, B. Foley, K. M. Hastie, M. D. Parker, D. G. Partridge, C. M. Evans, T. M. Freeman, T. I. de Silva, C. McDanal, L. G. Perez, H. Tang, A. Moon-Walker, S. P. Whelan, C. C. LaBranche, E. O. Saphire, D. C. Montefiori, A. Angyal, R. L. Brown, L. Carrilero, L. R. Green, D. C. Groves, K. J. Johnson, A. J. Keeley, B. B. Lindsey, P. J. Parsons, M. Raza, S. Rowland-Jones, N. Smith, R. M. Tucker, D. Wang, M. D. Wyles, Sheffield COVID-19 Genomics Group, Tracking changes in SARS-CoV-2 spike: Evidence that D614G increases infectivity of the COVID-19 virus. *Cell* **182**, 812–827.e19 (2020). doi:10.1016/j.cell.2020.06.043 Medline

21. T. de Oliveira, S. Lutucuta, J. Nkengasong, J. Morais, J. P. Paixão, Z. Neto, P. Afonso, J. Miranda, K. David, L. Inglês, A. P. A. P. Raisa Rivas Carralero, H. R. Freitas, F. Mufinda, S. K. Tessema, H. Tegally, E. J. San, E. Wilkinson, J. Giandhari, S. Pillay, M. Giovanetti, Y. Naidoo, A. Katzourakis, M. Ghafari, L. Singh, D. Tshiabuila, D. Martin, R. J. Lessells, A novel variant of interest of SARS-CoV-2 with multiple spike mutations is identified from travel surveillance in Africa. medRxiv 2021.03.30.21254323 (2021); https://doi.org/10.1101/2021.03.30.21254323.

22. B. Meng, S. A. Kemp, G. Papa, R. Datir, I. A. T. M. Ferreira, S. Marelli, W. T. Harvey, S. Lytras, A. Mohamed, G. Gallo, N. Thakur, D. A. Collier, P. Mlcochova, L. M. Duncan, A. M. Carabelli, J. C. Kenyon, A. M. Lever, A. De Marco, C. Saliba, K. Culap, E. Cameroni, N. J. Matheson, L. Piccoli, D. Corti, L. C. James, D. L. Robertson, D. Bailey, R. K. Gupta, COVID-19 Genomics UK (COG-UK) Consortium, Recurrent emergence of SARS-CoV-2 spike deletion H69/V70 and its role in the Alpha variant B.1.1.7. *Cell Rep.* **35**, 109292 (2021). doi:10.1016/j.celrep.2021.109292 Medline

23. S. E. James, T. Houriiyah, krisp-kwazulu-natal/africa-covid19-genomics: A year of genomic surveillance reveals how the SARS-CoV-2 pandemic unfolded in Africa - Code and scripts. Zenodo (2021); https://doi.org/10.5281/zenodo.5386379.

24. K. Katoh, D. M. Standley, MAFFT multiple sequence alignment software version 7: Improvements in performance and usability. *Mol. Biol. Evol.* **30**, 772–780 (2013). doi:10.1093/molbev/mst010 Medline

25. F. Wu, S. Zhao, B. Yu, Y.-M. Chen, W. Wang, Z.-G. Song, Y. Hu, Z.-W. Tao, J.-H. Tian, Y.-Y. Pei, M.-L. Yuan, Y.-L. Zhang, F.-H. Dai, Y. Liu, Q.-M. Wang, J.-J. Zheng, L. Xu,

E. C. Holmes, Y.-Z. Zhang, A new coronavirus associated with human respiratory disease in China. *Nature* **579**, 265–269 (2020). doi:10.1038/s41586-020-2008-3 Medline

26. L.-T. Nguyen, H. A. Schmidt, A. von Haeseler, B. Q. Minh, IQ-TREE: A fast and effective stochastic algorithm for estimating maximum-likelihood phylogenies. *Mol. Biol. Evol.* **32**, 268–274 (2015). doi:10.1093/molbev/msu300 Medline
27. F. Lemoine, J. B. Domelevo Entfellner, E. Wilkinson, D. Correia, M. Dávila Felipe, T. De Oliveira, O. Gascuel, Renewing Felsenstein's phylogenetic bootstrap in the era of big data. *Nature* **556**, 452–456 (2018). doi:10.1038/s41586-018-0043-0 Medline
28. P. Sagulenko, V. Puller, R. A. Neher, TreeTime: Maximum-likelihood phylodynamic analysis. *Virus Evol.* **4**, vex042 (2018). doi:10.1093/ve/vex042 Medline
29. A. Rambaut, E. C. Holmes, Á. O'Toole, V. Hill, J. T. McCrone, C. Ruis, L. du Plessis, O. G. Pybus, A dynamic nomenclature proposal for SARS-CoV-2 lineages to assist genomic epidemiology. *Nat. Microbiol.* **5**, 1403–1407 (2020). doi:10.1038/s41564-020-0770-5 Medline
30. A. J. Drummond, M. A. Suchard, D. Xie, A. Rambaut, Bayesian phylogenetics with BEAUti and the BEAST 1.7. *Mol. Biol. Evol.* **29**, 1969–1973 (2012). doi:10.1093/molbev/mss075 Medline
31. P. Lemey, A. Rambaut, J. J. Welch, M. A. Suchard, Phylogeography takes a relaxed random walk in continuous space and time. *Mol. Biol. Evol.* **27**, 1877–1885 (2010). doi:10.1093/molbev/msq067 Medline
32. A. Rambaut, T. T. Lam, L. Max Carvalho, O. G. Pybus, Exploring the temporal structure of heterochronous sequences using TempEst (formerly Path-O-Gen). *Virus Evol.* **2**, vew007 (2016). doi:10.1093/ve/vew007 Medline
33. S. Dellicour, R. Rose, N. R. Faria, P. Lemey, O. G. Pybus, SERAPHIM: Studying environmental rasters and phylogenetically informed movements. *Bioinformatics* **32**, 3204–3206 (2016). doi:10.1093/bioinformatics/btw384 Medline
34. E. Wilkinson, D. Rasmussen, O. Ratmann, T. Stadler, S. Engelbrecht, T. de Oliveira, Origin, imports and exports of HIV-1 subtype C in South Africa: A historical perspective. *Infect. Genet. Evol.* **46**, 200–208 (2016). doi:10.1016/j.meegid.2016.07.008 Medline
35. J. Sukumaran, M. T. Holder, DendroPy: A Python library for phylogenetic computing. *Bioinformatics* **26**, 1569–1571 (2010). doi:10.1093/bioinformatics/btq228 Medline
36. D. Sankoff, Minimal mutation trees of sequences. *SIAM J. Appl. Math.* **28**, 35–42 (1975). doi:10.1137/0128004
37. International Trade Statistics Database, UN Comtrade Database; https://comtrade.un.org/.
38. Johns Hopkins University (JHU) Center for Systems Science and Engineering (CSSE), CSSEGISandData/COVID-19, Novel coronavirus (COVID-19) cases, GitHub; https://github.com/CSSEGISandData/COVID-19.
