# Supplementary material for: A year of genomic surveillance reveals how the SARS-CoV-2 pandemic unfolded in Africa
Source: Science. 2021 Sep 9;374(6566):423–31. doi: 10.1126/science.abj4336 (PMC7613315; doi:10.1126/science.abj4336)
Supplement: Supplementary file 3 — Table S4 [file science.abj4336_table_s4.zip › science.abj4336_caption_table_s4.docx]

**Supplementary Table S4:** GISAID Acknowledgements Table supplied as an Excel attachment
