# Supplementary material for: A year of genomic surveillance reveals how the SARS-CoV-2 pandemic unfolded in Africa
Source: Science. 2021 Sep 9;374(6566):423–31. doi: 10.1126/science.abj4336 (PMC7613315; doi:10.1126/science.abj4336)
Supplement: Supplementary file 4 — MDAR Reproducibility Checklist [file science.abj4336_mdar_reproducibility_checklist.pdf]

# Materials Design Analysis Reporting (MDAR) Checklist for Authors

The MDAR framework establishes a minimum set of requirements in transparent reporting applicable to studies in the life sciences (see Statement of Task: doi:10.31222/osf.io/9sm4x.). The MDAR checklist is a tool for authors, editors and others seeking to adopt the MDAR framework for transparent reporting in manuscripts and other outputs.   Please refer to the MDAR Elaboration Document for additional context for the MDAR framework.

## Materials

| Antibodies | Yes (indicate where provided: page no/section/legend) | n/a |
|---|---|---|
| For commercial reagents, provide supplier name, catalogue number and RRID, if available. | | n/a |

| Cell materials | Yes (indicate where provided: page no/section/legend) | n/a |
|---|---|---|
| **Cell lines:** Provide species information, strain. Provide accession number in repository **OR** supplier name, catalog number, clone number, **OR** RRID | | n/a |
| **Primary cultures:** Provide species, strain, sex of origin, genetic modification status. | | n/a |

| Experimental animals | Yes (indicate where provided: page no/section/legend) | n/a |
|---|---|---|
| **Laboratory animals:** Provide species, strain, sex, age, genetic modification status. Provide accession number in repository **OR** supplier name, catalog number, clone number, **OR** RRID | | n/a |
| **Animal observed in or captured from the field:** Provide species, sex and age where possible | | n/a |
| **Model organisms:** Provide Accession number in repository (where relevant) **OR** RRID | | n/a |

| Plants and microbes | Yes (indicate where provided: page no/section/legend) | n/a |
|---|---|---|
| **Plants:** provide species and strain, unique accession number if available, and source (including location for collected wild specimens) | | n/a |
| **Microbes:** provide species and strain, unique accession number if available, and source | | n/a |

| Human research participants | Yes (indicate where provided: page no/section/legend) | n/a |
|---|---|---|
| Identify authority granting ethics approval (IRB or equivalent committee(s), provide reference number for approval. | Provided in "Ethics Statement" section on Page 1 of the Supplementary Materials | |
| Provide statement confirming informed consent obtained from study participants. | | **n/a** |
| Report on age and sex for all study participants. | | **n/a** |

## Design

| Study protocol | Yes (indicate where provided: page no/section/legend) | n/a |
|---|---|---|
| For clinical trials, provide the trial registration number **OR** cite DOI in manuscript. | | n/a |

| Laboratory protocol | Yes (indicate where provided: page no/section/legend) | n/a |
|---|---|---|
| Provide DOI or other citation details if detailed step-by-step protocols are available. | | n/a |

| Experimental study design (statistics details) | Yes (indicate where provided: page no/section/legend) | n/a |
|---|---|---|
| State whether and how the following have been done**, or** if they were not carried out. | | |
| Sample size determination | At the time of writing, 10326 African complete and near-complete genome sequences were available in GISAID, and 8,746 genomes that passed quality control were used in this analysis. We believe this sample size was sufficient because the genomes come from 35 countries across the continent. | |
| Randomisation | Samples for SARS-CoV-2 sequencing in different African countries were randomly selected as part of various sampling strategies outlined in Supplementary Table S3. | |
| Blinding | Geographical blinding of data was not necessary for the study as it involves phylogeographical analysis, however the exact name of the health facilities associated with the genomic samples were anonymized. Data identification from the samples were also anonymized as this was not necessary for the analysis. | |
| Inclusion/exclusion criteria | Prior to phylogenetic reconstruction we removed low quality sequences, which included those identified as being of low quality by NextClade (n=18; https://clades.nextstrain.org), those with missing sampling dates (n = 189), those with <90% coverage (n = 1017), those with > 40 SNPs (n = 39), those with >10 ambiguous base-calls per genome (n = 128), and those with clustered SNPs (n = 189). | |

| Sample definition and in-laboratory replication | Yes (indicate where provided: page no/section/legend) | n/a |
|---|---|---|
| State number of times the experiment was replicated in laboratory | | n/a |
| Define whether data describe technical or biological replicates | Replication was performed for maximum likelihood and bayesian MCMC phylogenetic tree reconstructions. For Maximum Likelihood reconstruction of the phylogeny we performed 100 bootstrap replicates. We computed MCMC (Markov chain Monte Carlo) triplicate runs of 100 million states each, sampling every 10,000 steps for the clusters of interest. All attempts at replication were successful and the MCC tree for the clusters were of high support. | |

| Ethics | Yes (indicate where provided: page no/section/legend) | n/a |
|---|---|---|
| Studies involving human participants: State details of authority granting ethics approval (IRB or equivalent committee(s), provide reference number for approval. | Provided in "Ethics Statement" section on Page 1 of the Supplementary Materials | |

| | | |
|---|---|---|
| Studies involving experimental animals: State details of authority granting ethics approval (IRB or equivalent committee(s), provide reference number for approval. | | n/a |
| Studies involving specimen and field samples: State if relevant permits obtained, provide details of authority approving study; if none were required, explain why. | | n/a |

| **Dual Use Research of Concern (DURC)** | **Yes (indicate where provided: page no/section/legend)** | **n/a** |
|---|---|---|
| If study is subject to dual use research of concern, state the authority granting approval and reference number for the regulatory approval | | n/a |

## Analysis

| Attrition | Yes (indicate where provided: page no/section/legend) | n/a |
|---|---|---|
| State if sample or data point from the analysis is excluded, and whether the criteria for exclusion were determined and specified in advance. | Prior to phylogenetic reconstruction we removed low quality sequences, which included those identified as being of low quality by NextClade (n=18; https://clades.nextstrain.org), those with missing sampling dates (n = 189), those with <90% coverage (n = 1017), those with > 40 SNPs (n = 39), those with >10 ambiguous base-calls per genome (n = 128), and those with clustered SNPs (n = 189). | |

| Statistics | Yes (indicate where provided: page no/section/legend) | n/a |
|---|---|---|
| Describe statistical tests used and justify choice of tests. | Replication was performed for maximum likelihood and bayesian MCMC phylogenetic tree reconstructions. For Maximum Likelihood reconstruction of the phylogeny we performed 100 bootstrap replicates. We computed MCMC (Markov chain Monte Carlo) triplicate runs of 100 million states each, sampling every 10,000 steps for the clusters of interest. All attempts at replication were successful and the MCC tree for the clusters were of high support. | |

| Data Availability | Yes (indicate where provided: page no/section/legend) | n/a |
|---|---|---|
| State whether newly created datasets are available, including protocols for access or restriction on access. | All sequences that were used in the present study, both newly generated and publicly available, are listed in Supplementary Table S4 (accessible on the GitHub repository) along with their GISAID sequence IDs, dates of sampling, the originating and submitting laboratories and main authors. | |
| If data are publicly available, provide accession number in repository or DOI or URL. | - Supplementary Table S4 (accessible on the GitHub repository) contains GISAID sequence IDs, dates of sampling, the originating and submitting laboratories and main authors.<br>- All other materials are shared publicly on GitHub (https://github.com/krisp-kwazulu-natal/africa-covid19-genomics). | |
| If publicly available data are reused, provide accession number in repository or DOI or URL, where possible. | - https://github.com/krisp-kwazulu-natal/africa-covid19-genomics | |

| Code Availability | Yes (indicate where provided: page no/section/legend) | n/a |
|---|---|---|
| For all newly generated code and software essential for replicating the main findings of the study: | | |
| State whether the code or software is available. | All input files (e.g. alignments or XML files), all resulting output files and scripts used in the study are shared publicly on GitHub (https://github.com/krisp-kwazulu-natal/africa-covid19-genomics). | |
| If code is publicly available, provide accession number in repository, or DOI or URL. | https://github.com/krisp-kwazulu-natal/africa-covid19-genomics | |

## Reporting

| Adherence to community standards | Yes (indicate where provided: page no/section/legend) | n/a |
|---|---|---|
| MDAR framework recommends adoption of discipline-specific guidelines, established and endorsed through community initiatives. Journals have their own policy about requiring specific guidelines and recommendations to complement MDAR. | | n/a |
| State if relevant guidelines (eg., ICMJE, MIBBI, ARRIVE) have been followed, and whether a checklist (eg., CONSORT, PRISMA, ARRIVE) is provided with the manuscript. | | n/a |
